# Syntheses and Applications of (Thio)Urea-Containing Chiral Quaternary Ammonium Salt Catalysts

**DOI:** 10.1002/ejoc.201301594

**Published:** 2014-02

**Authors:** Johanna Novacek, Mario Waser

**Affiliations:** [a] Institute of Organic Chemistry, Johannes Kepler University Linz, Altenbergerstraße 69, 4040 Linz, Austria

**Keywords:** Phase-transfer catalysis, Hydrogen bonds, Chirality, Enantioselectivity, Fluorination

## Abstract

We herein report our efforts to obtain a new class of systematically modified bifunctional (thio)urea-containing quaternary ammonium salts based on easily obtainable chiral backbones. Among the different classes of catalysts that were successfully synthesized, those based on *trans*-1,2-cyclohexane diamine were found to be the most powerful for the asymmetric α-fluorination of β-keto esters. Selectivities up to 93:7 could be obtained by using only 2 mol-% of the optimized catalyst. The importance of the bifunctional nature of these catalysts was demonstrated by control experiments using simplified monofunctional catalyst analogues, which gave almost racemic product only.

## Introduction

The use of chiral ammonium salt phase-transfer catalysts (PTCs) has contributed significantly to the field of asymmetric catalysis.^[1]^ Compared to others, this catalytic strategy is outstanding because it represents the only generally applicable catalytic principle that is capable of stereoselectively activating such important nucleophiles as, for example, enolates, cyanides, or peroxides in a noncovalent and easy to handle fashion.^[1]^ Besides monofunctional chiral ammonium salt catalysts, the use of bifunctional derivatives has proven to be a powerful strategy to facilitate reactions where other methods clearly fail.^[2]^ It is noteworthy that the vast majority of such catalysts contain an additional hydroxyl group (phenolic or aliphatic) as the second coordination site,^[2-4]^ whereas the incorporation of alternative catalytically active motifs has so far been less exhaustively investigated.^[2,5-10]^

**Figure 1:**
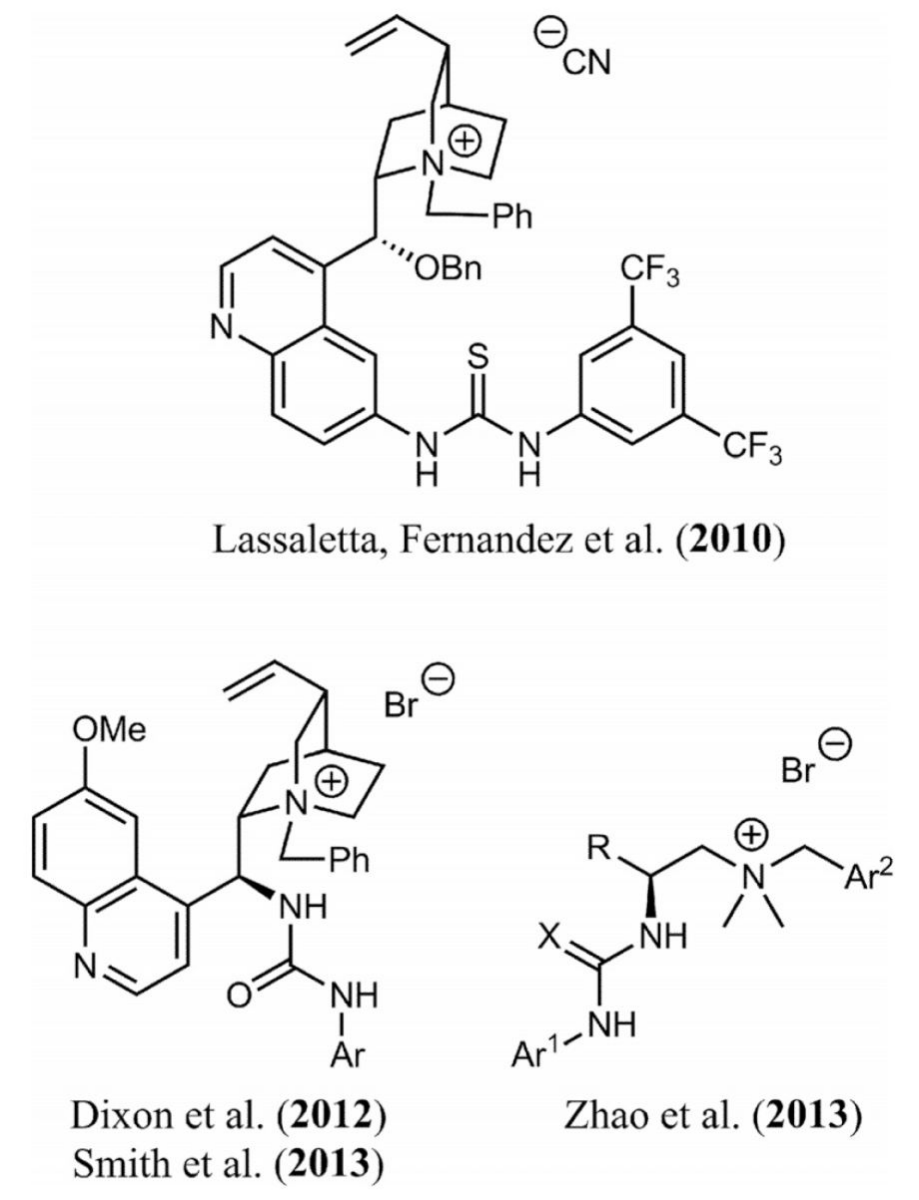
Known (thio)urea-containing bifunctional ammonium salt catalysts.^[7-9]^

Among the commonly employed hydrogen-bonding donors, (thio)ureas have been shown to be excellent catalysts in numerous case studies.^[11,12]^ The successful combination of their high potential to activate and control the reactivity of electrophiles combined with the unique nucleophile activation potential of quaternary ammonium salts should therefore result in a remarkable bifunctional catalytic system. Surprisingly, investigations concerning syntheses and applications of such catalysts are not common.^[7-9]^ To the best of our knowledge, only one example describing the synthesis and application of a thiourea-containing cinchona alkaloid PTC^[7]^ and two reports concerning urea-containing cinchona PTCs^[8]^ have been published (Figure 1). As illustrated in these reports, one of the major challenges is the synthesis of these catalysts,^[7,8]^ thus explaining why only a limited number of different catalysts have been described. During the preparation of this manuscript, the Zhao group reported an elegant strategy with which to access a diversified library of α-amino acid based (thio)urea-containing quaternary ammonium salts that were found to be powerful catalysts for asymmetric aza-Henry reactions and the addition of thiols to imines (Figure 1).^[9]^

**Figure 2:**
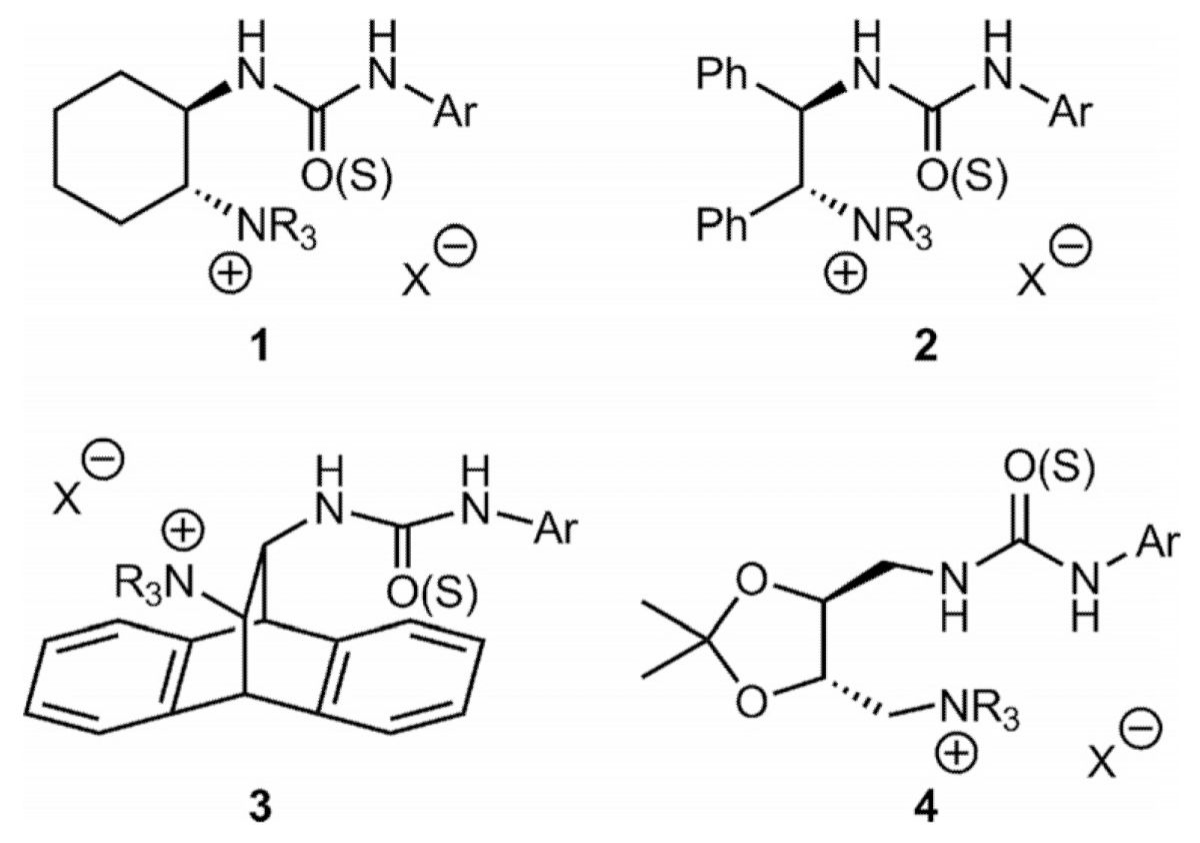
Targeted bifunctional ammonium salt catalysts.

Spurred on by the potential of such bifunctional catalysts for asymmetric applications and the limited number of different catalyst systems available so far, we carried out a systematic feasibility study focusing on the development of flexible and functional-group tolerant synthetic routes to access a carefully diversified library of (thio)urea-containing catalysts based on different, easily available chiral scaffolds as shown in Figure 2. Hereby, access to both antipodes of the catalysts will be possible, thus overcoming some of the major limitations that are apparent when using the pseudoenantiomeric cinchona alkaloid catalysts.

## Results and Discussion

### Catalyst Syntheses

Recent reports described incorporation of the H-bonding donor first, followed by a final quaternarization step.^[7-9]^ However, this strategy only gave access to a limited number of modified catalysts, especially when using cinchona alkaloids, because of possible side reactions of the nucleophilic heteroatoms of the H-bonding donor with the alkylating agent.^[13]^ We have therefore focused on an alternative sequence proceeding through an early quaternarization, followed by late-stage incorporation of the H-bonding donor.

**Scheme 1:**
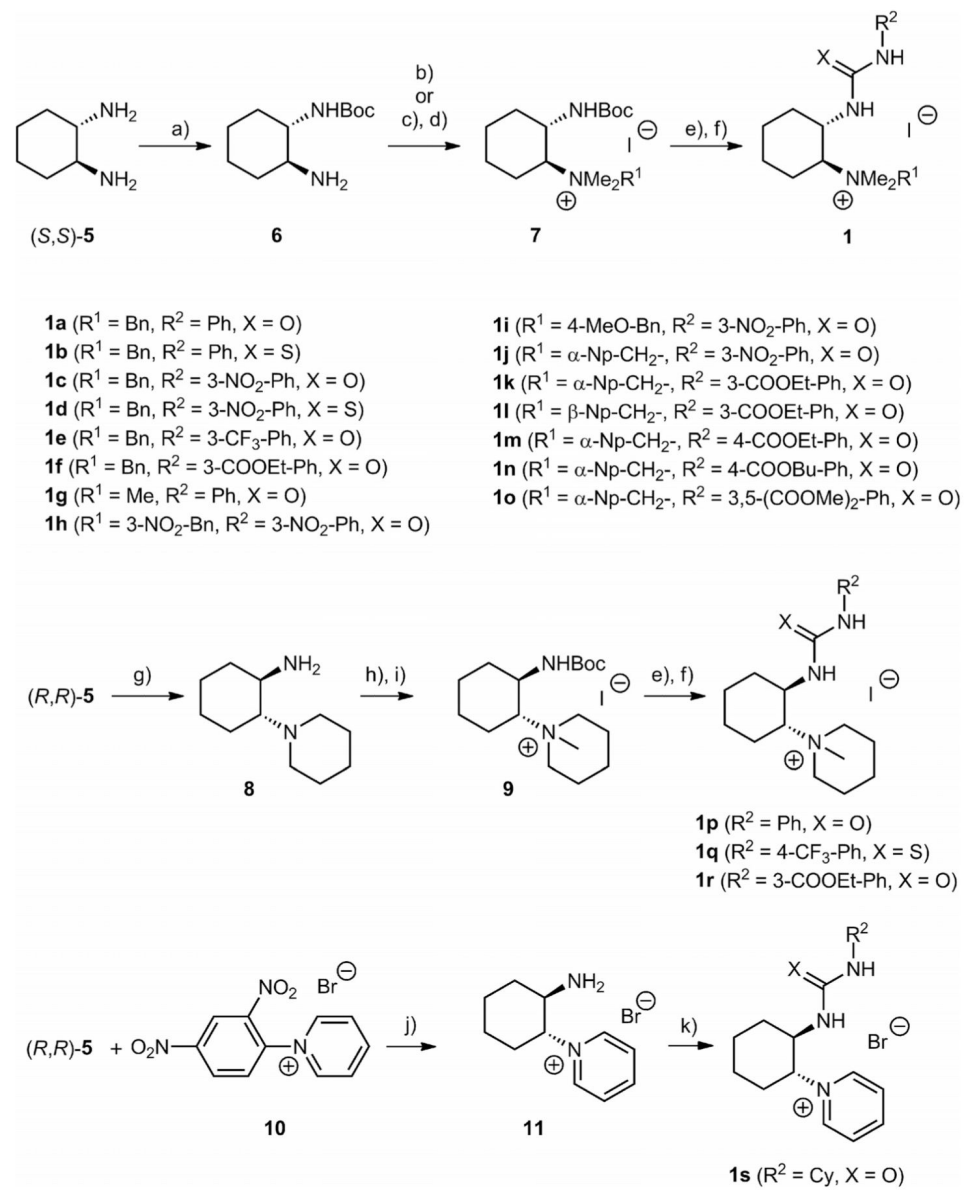
Syntheses of cyclohexanediamine-based catalysts **1** tested in asymmetric applications.^[16]^ *Reagents and conditions:*^[18]^ (a) According to ref.^[19]^ (b) MeI (5 equiv.), K_2_CO_3_ (2 equiv.), CH_3_CN, room temp., 3 d, quant.; (c) R^1^CHO (1 equiv.), NaBH_4_ (1.5 equiv.), MeOH/THF (1:1), room temp., 4 h; (d) MeI (excess), K_2_CO_3_ (2 equiv.), reflux, 3–5 d, 24–64% (two steps); (e) CF_3_COOH (10 equiv.) or HI (57% in H_2_O, 10 equiv.), CH_2_Cl_2_, room temp., 2–16 h; (f) R^2^NCX (1–1.5 equiv.), K_2_CO_3_ (3 equiv.), CH_2_Cl_2_ or CH_3_CN, room temp., 8–18 h, 31–71% (two steps); (g) According to ref.^[20]^ (h) Boc_2_O (1.2 equiv.), CH_2_Cl_2_, room temp., 20 h, 72%; (i) MeI (excess), K_2_CO_3_ (1 equiv.), reflux, 3 d, 45%; (j) DMSO, 90 °C, 1 d; (k) CyNCO (1.5 equiv.), K_2_CO_3_ (1 equiv.), CH_2_Cl_2_, room temp., 18 h, 41% (two steps).

The main chiral backbone used for these studies was 1,2-*trans*-cyclohexanediamine (**5**) (Scheme 1).^[14]^ After mono-Boc-protection,^[15]^ quaternarization could be achieved either by treating **6** with an excess of methyliodide (to obtain trimethylammonium salt-based intermediates **7**) or by carrying out a reductive amination with different arylcarbaldehydes first, followed by methylation. It is worth noting that, whereas direct exhaustive methylation of **6** could be achieved easily, alkylation of the benzylated *sec*-amine intermediates was found to be significantly slower (usually resulting in around 30% of recovered *tert*-amine intermediate, which could be resubmitted to the methylation). Even more interestingly, when methylating the naphthyl-methyl-containing intermediates en route to catalysts **1j**-**o**, the yield dropped to around 30% (50-60% based on recovered *tert*-amine intermediate, which could be methylated again) because the corresponding intermediates **7** were found to decompose to some extent under prolonged exposure to base under the reaction conditions. In addition, dibenzylated *tert*-amines could not be quaternated any more. Nevertheless, we were able to obtain sufficient quantities of intermediates **7** and, after Boc-deprotection, the final coupling with different isocyanates or isothiocyanates was found to be easily possible, thus giving a series of substituted bifunctional ammonium salts **1a**-**o** in a straightforward manner.^[16]^ Alternatively, it was also possible to introduce a piperidine moiety first to give intermediate **8**, which could then be used to access catalysts **1p**-**r** analogously. In addition, we were also able to synthesize the pyridinium-containing catalyst **1s** by employing the Zincke salt **10** to introduce a pyridinium moiety in the first step.^[17]^

**Scheme 2:**
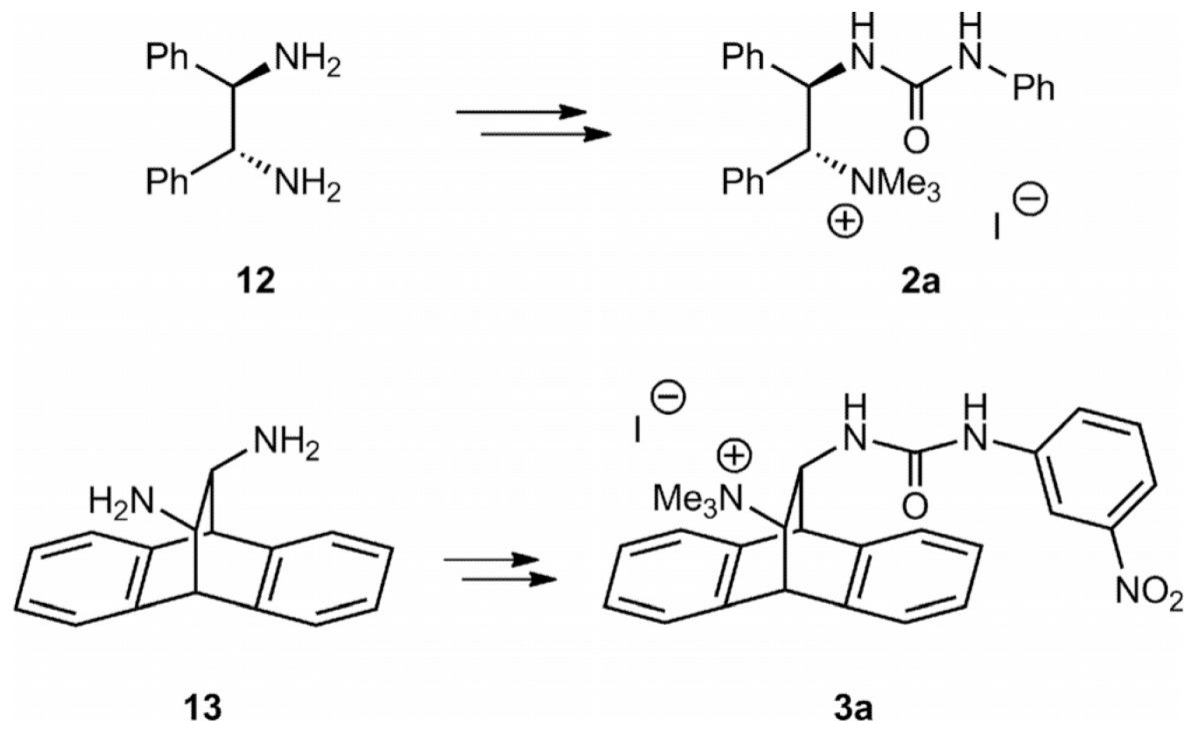
Catalysts **2** and **3** synthesized and tested herein.

With a robust route to access a carefully diversified library of cyclohexanediamine-based catalysts **1** at hand, we next employed the same strategy to access catalysts **2** and **3** (Scheme 2). It should be pointed out that the developed strategy allows for the synthesis of a larger variety of different catalysts of type **2** and **3**. However, for the first screening in our asymmetric test reaction to determine the best-suited chiral backbone, we initially just synthesized the derivatives **2a** and **3a** shown in Scheme 2, because these compounds allowed for a direct comparison with the analogous cyclohexanediamine-based catalysts.

**Scheme 3:**
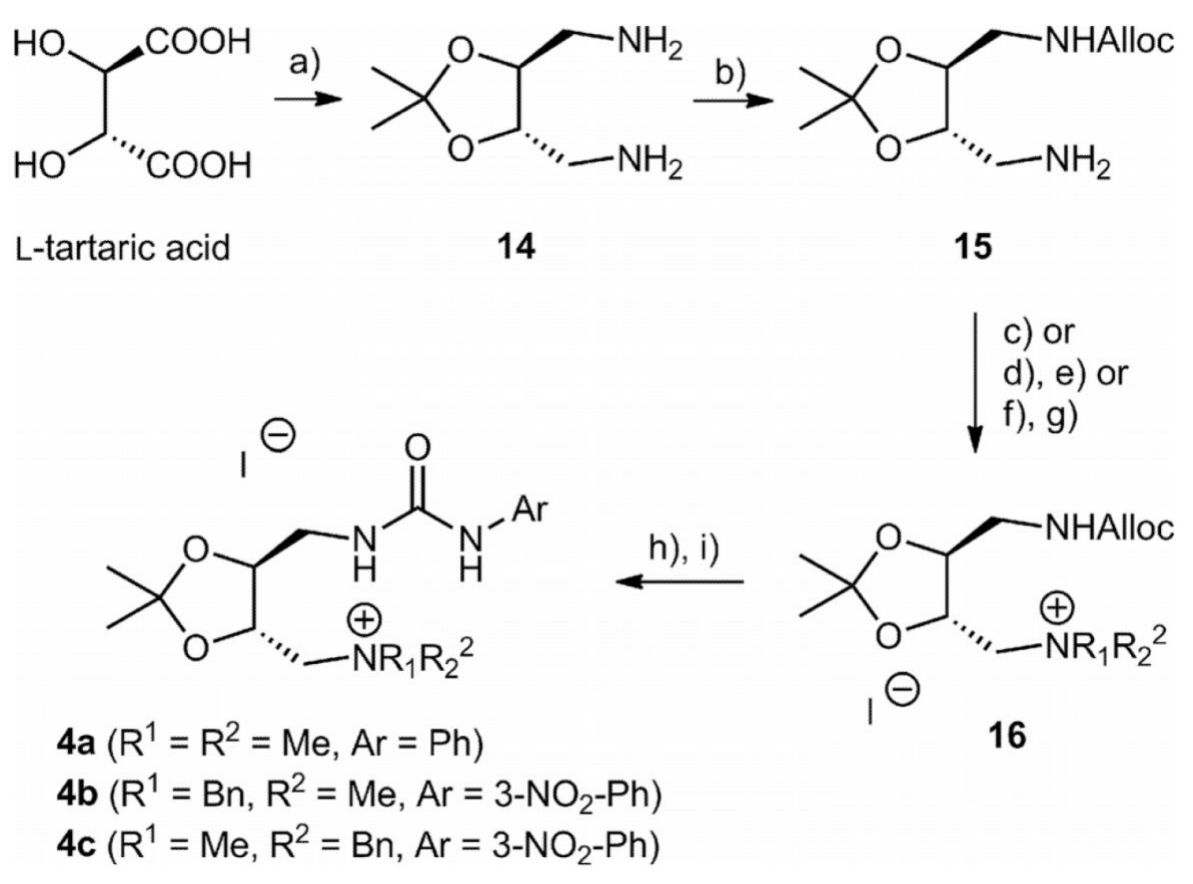
Synthesis of catalysts **4** tested herein. *Reagents and conditions:*^[18]^ (a) According to Ref.^[23]^ (b) allyl chloroformate (0.4 equiv.), CH_2_Cl_2_, 0 °C, 16 h; (c) MeI (5 equiv.), K_2_CO_3_ (2 equiv.), CH_3_CN, reflux, 3 d, 23% (two steps); (d) PhCHO (1 equiv.), NaBH_4_ (1.5 equiv.), MeOH/THF (1:1), room temp., 16 h; (e) MeI (excess), K_2_CO_3_ (2 equiv.), reflux, 3 d, 11% (three steps); (f) BnBr (2.5 equiv.), K_2_CO_3_ (2 equiv.), CH_3_CN, reflux, 16 h; (g) MeI (excess), K_2_CO_3_ (1 equiv.), reflux, 2 d, 22% (three steps); (h) [Pd(PPh_3_)_4_] (5%), NaBH_4_ (3 equiv.), CH_2_Cl_2_/MeOH (1:1), room temp., 2 h; (i) ArNCO (1 equiv.), K_2_CO_3_ (1.5 equiv.), CH_2_Cl_2_, room temp., 20 h, 40–69% (two steps).

In addition to catalysts **1**-**3**, which are based on a chiral C2-linker, we were also interested in obtaining catalysts with a chiral C4-linker. Based on our recent experience in developing tartaric acid-derived organocatalysts,^[21,22]^ we investigated the synthesis of catalysts **4** starting from known diamine **14**^[23]^ (Scheme 3). Due to the presence of an acid-labile acetonide moiety, the Boc-protecting group was not tolerated herein. After some experimentation, the Alloc-group was found to be best-suited for our approach. The mono-protection step was found to be less selective than in the case of the Boc-procedure employed for the synthesis of **6** (see Scheme 1) and even when only 0.4 equiv. allyl chloroformate was used, still around 15% di-alloc-protected diamine was formed. Nevertheless, we were able to obtain intermediate **15** in reasonable quantities under these unoptimized conditions. Quaternarization was found to be significantly faster than in the syntheses of catalysts **1**-**3**, giving also access to dibenzylated ammonium salts, which can be attributed to the lower steric hindrance in the tartaric acid-derived system. After deprotection with NaBH_4_ under Pd-catalysis, coupling with different isocyanates to obtain the urea-containing catalysts **4** was easily accomplished. Because it was found that urea-containing catalysts **1** were, in general, more powerful than thioureas in our test reaction (see Table 1), we initially only synthesized and screened the urea-containing ammonium salts **4** shown in Scheme 3.

### Catalytic Potential

To evaluate the catalytic potential of the new bifunctional catalysts, the asymmetric α-fluorination of β-keto esters **17** was chosen as a test reaction.^[24]^ This reaction has been carried out in the past by using chiral PTCs with good to excellent selectivities.^[25]^ Noteworthy, Maruoka et al. reported the beneficial effect of bifunctional free-hydroxyl-containing binaphthyl-based catalysts for this reaction,^[25b]^ whereas the groups of Lu and Meng observed a detrimental effect of free-hydroxyl-groups when using cinchona alkaloid based PTCs.^[25d]^ On the other hand, the use of chiral (thio)-ureas has so far only been briefly investigated for this important reaction,^[26]^ thus making it a challenging test reaction for our bifunctional catalysts.

An overview of the most significant results obtained in a very detailed screening of different catalysts and reaction conditions for the fluorination of *tert*-butyl ester **17a** using NFSI (**18**) as the fluorinating agent is summarized in Table 1.^[27]^ Initial screening was carried out by using cyclohexanediamine-based catalysts **1**. We first found that, for this specific reaction, the urea-containing catalysts (such as **1a**) performed better than the corresponding thioureas (e.g., **1b**) (entries 1 and 2). In addition, an electron-withdrawing group on the urea moiety was found to be beneficial, leading to a promising initial enantiomeric ratio (*er*) of 80:20 under unoptimized conditions (entry 3). In all these initial experiments, full conversion of starting material within five hours at 0 °C was observed (isolated yields of these small-scale experiments were typically around 80%). It is note-worthy that using just catalytic amounts of base (entry 4), the selectivity dropped significantly, accompanied with a slightly slower conversion rate. It also should be noted that the racemic background reaction proceeds reasonably fast without any catalyst under liquid/liquid and liquid/solid biphasic conditions, thus illustrating the need for highly potent catalysts to control this reaction.

The use of a solid base gave **19a** with slightly lower enantioselectivity (Table 1, entry 5). Testing the alternative catalyst moieties **2** and **3**, we found that neither could compete with catalysts **1** (entries 6 and 7). Thus, no further attempts to optimize and fine-tune these ammonium salts were undertaken. Moreover, the pyridinium-containing catalyst **1s** (entry 8) did not meet the potential of **1c** (we also synthesized a pyridinium-based catalyst **1** containing an aryl-substituent on the urea-moiety that performed in an even less selective manner). The lower potential of thioureas was proven again by using piperidinium-based catalyst **1q**. Interestingly, the tartaric acid-based catalyst **4b** performed only slightly less selectively than **1c** (compare entries 3 and 11). Nevertheless, because catalysts **1** were found to be more potent than those based on alternative chiral skeletons, further testing and optimization was conducted by using this chiral backbone. Catalyst **1c** was then employed to screen a variety of different liquid/liquid conditions (entries 12–23). Thereby, we identified aqueous K_3_PO_4_ (0.5 m, 2 equiv.) as the best-suited mild inorganic base (aqueous carbonates performed similarly well, compare entries 3 and 15). The reaction could be performed in a similarly selective manner by using different amounts of catalysts (1–20 mol-% were tested), thus showing the high potential of these catalysts to control this fast reaction even using low catalyst loadings (entries 16–18; using less than 1 mol-% resulted in reduced selectivity). Among a variety of different solvents, *m*-xylene was found to facilitate the highest selectivity.

Having identified the biphasic system *m*-xylene/aqueous K_3_PO_4_ (0.5 m, 2 equiv.) to be the best suited, giving **19a** with 82:18 *er* by using catalyst **1c** at 0 °C, a series of substituted catalysts **1** was then screened. As expected, thiourea-containing **1d** was found to be less selective (Table 1, entry 25), although this was less pronounced than in the previous experiments. Testing differently substituted ammonium groups and different ureas, it was observed that increasing the steric bulk on the ammonium moiety (for example by using a naphthyl group) and using ester-containing aryl on the urea group, was beneficial (see entries 29 and 32). Accordingly, combining these structural features in catalyst **1k** was found to be particularly advantageous (entry 33). The α-naphthyl group was found be slightly better-suited than a β-naphthyl for our purpose (entries 33 vs. 34). Attempts to further modify other parent systems were less satisfactory (entries 35 and 36). More interestingly, the use of dibenzylated tartaric acid-based catalyst **4c** resulted in a significantly lower selectivity than using monobenzylated **4b** (entries 36 vs. 11).

Further variation of catalysts **1** by introducing different esters on the aryl group of the ureas showed that *para*-substitution leads to a slightly reduced selectivity (Table 1, entries 37 and 38). In contrast *meta*-disubstitution resulted in the reasonably selective bifunctional ammonium salt catalyst **1o** (entry 39). The selectivity could be slightly improved further by carrying out the reaction at −10 °C (entry 40), whereas further reduction in temperature had no further beneficial effect (entry 41).

**Scheme 4:**
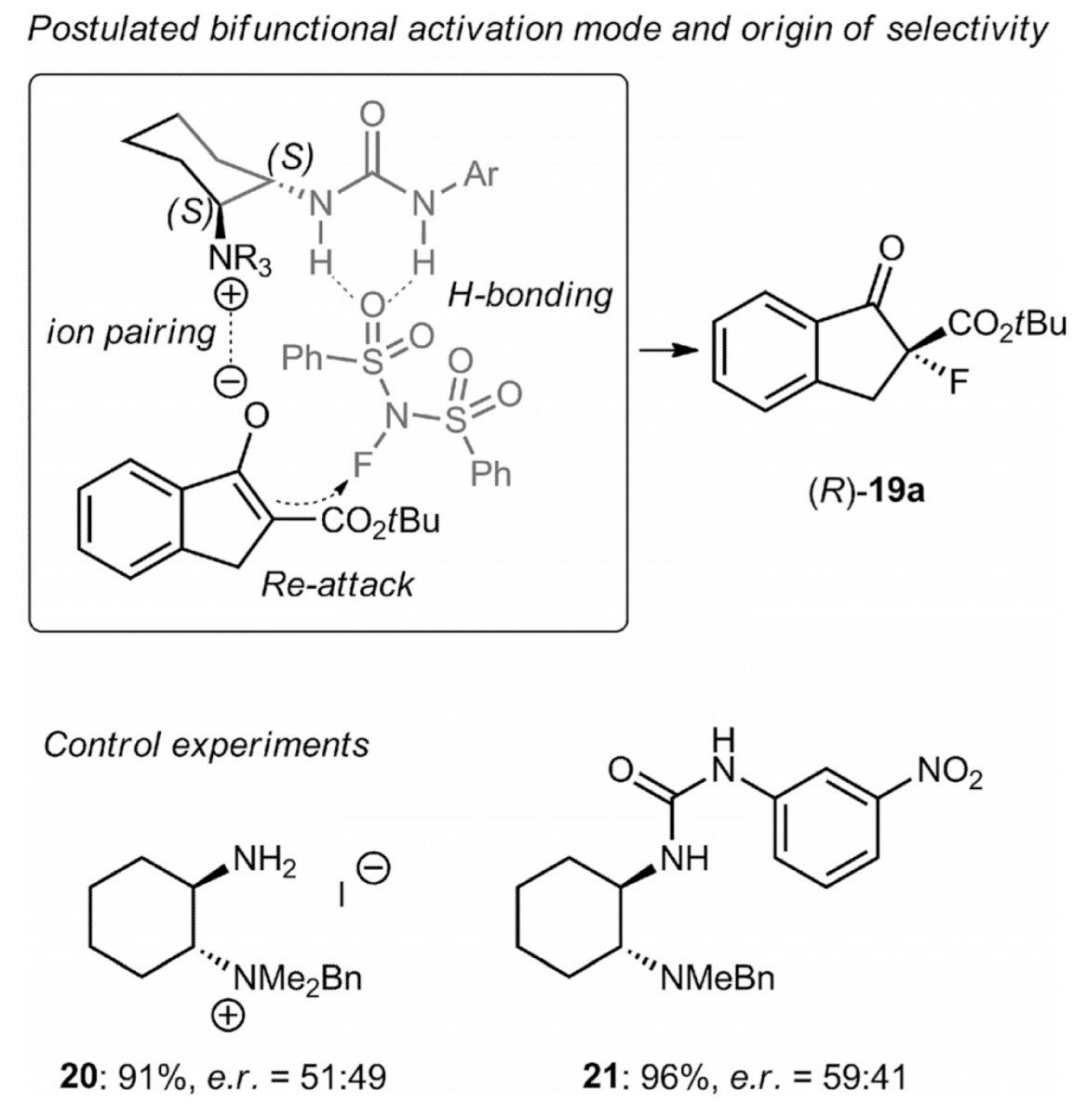
Proposed activation mode of these bifunctional catalysts and control experiments performed by using the simplified catalysts **20** and **21**.

The observed enantioselectivities in all these experiments (also compared with the results obtained by using the opposite configured catalysts **1p**-**r** and **4**) are in accordance with the proposed bifunctional activation model depicted in Scheme 4. Whereas the ammonium group is supposed to activate the enolate of the keto ester, the H-donor group is believed to coordinate the NFSI, thus leading to a highly ordered transition state. Because of the need for a stoichiometric amount of base to obtain high selectivities (see Table 1, entry 4) formation of the enolate and, accordingly, ion pairing with the catalyst, seems crucial. To further prove the necessity for both functional groups we also tested catalyst **20**, which lacks the H-bonding donor unit, and **21**, containing a *tert*-amine instead of the ammonium group, under these reaction conditions and found that both gave **19a** with rather low enantioselectivity.

Finally, having identified the most active catalyst **1o** and the best-suited reaction conditions for this transformation, a short screening of different β-keto esters was carried out (Table 2). All reactions were carried out at −10 °C by using 2 mol-% catalyst **1o** and, in each experiment, complete conversion of the keto ester within 12 h was observed. As can be seen from the results shown in entries 1–5, the nature of the ester group has a pronounced effect on the observed enantioselectivity. The methyl and benzyl esters **17b** and **17c**, respectively, gave the corresponding products with lower selectivities of 85:15. The cumyl ester **17d** (which was recently found to give good selectivities in phase-transfer catalyzed α-alkynylation reactions of β-keto esters^[28]^) also performed slightly less selectively (the corresponding product was also found to be rather sensitive towards hydrolysis and decarboxylation). On the other hand, the sterically more demanding adamantyl ester **17e** gave product **19e** with a reasonably high enantiomeric ratio of 93:7 (entry 5). The same tendency was also observed when using indanone derivatives **22**, **24**, and **26**. In each case, the adamantyl esters led to slightly more selectivity than the corresponding *tert*-butyl esters (entries 6–11). Tetralone-based ester **28** could also be successfully employed albeit with a slightly lower selectivity than **17a**, which is also consistent with reports by others.^[25b]^ Finally, the cyclopentanone-based ester **30** could also be employed with similar selectivity (entry 13).

## Conclusions

We succeeded in introducing a novel class of bifunctional (thio)urea-containing quaternary ammonium salts based on easily obtainable chiral backbones. The developed modular synthetic approach allowed us to access a carefully diversified library of different catalysts. Among the different classes tested herein, those ammonium salts based on *trans*-1,2-cyclohexane diamine were found to be the most powerful catalysts for the asymmetric α-fluorination of β-keto esters. By using the optimized catalyst **1o**, selectivities up to 93:7 could be obtained by using 2 mol-% catalyst. The importance of the bifunctional nature of these catalysts was clearly demonstrated by control experiments using simplified monofunctional catalyst analogues, which gave almost racemic product only. Further investigations to obtain even more powerful catalysts and to elucidate the application scope of these catalysts for other transformation, as well as studies concerning the actual activation mode, are ongoing and will be reported in due course.

## Experimental Section

### General Information

^1^H and ^13^C NMR spectra were recorded with a Bruker Avance III 300 MHz spectrometer and with a Bruker Avance III 700 MHz spectrometer with a TCI cryoprobe; all NMR spectra were referenced to the solvent peak. High-resolution mass spectra were obtained with an Agilent 6520 Q-TOF mass spectrometer with an ESI source and an Agilent G1607A coaxial sprayer. All analyses were made in the positive ionization mode. Purine (*m*/*z* 121.050873 [M + H]^+^) and 1,2,3,4,5,6-hexakis(2,2,3,3-tetrafluoropropoxy)-1,3,5,2,4,6-triazatriphosphinane (*m*/*z* 922.009798 [M + H]^+^) were used for internal mass calibration. IR spectra were recorded with a Shimadzu IR Affinity-1 Fourier transform infrared spectrometer. Optical rotations were recorded with a Perkin–Elmer Polarimeter Model 241 MC and with a Schmidt and Haensch Polarimeter Model UniPol L 1000. HPLC was performed with a Dionex Summit HPLC system with a Chiralcel OD-H (250×4.6 mm), a Chiralcel OD-R (250×4.6 mm, 10 μm), or a Chiralpak AD-H (250 ×4.6 mm, 5 μm) chiral stationary phase. All chemicals were purchased from commercial suppliers and used without further purification unless otherwise stated. All reactions were performed under an Ar-atmosphere. Starting β-keto esters were either purchased from commercial suppliers or prepared according to reported methods.^[25,29]^

A detailed experimental section including all the procedures and analytical data of catalysts and fluorination products as well as copies of NMR spectra and HPLC chromatograms can be found in the Supporting Information.

### Synthesis of Catalyst 1o

#### Compound 7d (R^1^ = α-Np-CH_2_-)

**Step 1:** 1-Naphthaldehyde (780 mg, 5 mmol) was added to a solution of **6** (1.07 g, 5 mmol) [prepared from the dihydrochloride of (*S*,*S*)-cyclohexanediamine (**5**)^[30]^ according to a published procedure^[19]^] in THF/MeOH (1:1, 20 mL) and the solution was stirred at room temp. for 2 h. After the addition of NaBH_4_ (285 mg, 1.5 equiv.), stirring was continued for another 2 h at room temp. The reaction was quenched by addition of H_2_O and extracted with H_2_O/Et_2_O. The organic phase was washed with brine, dried with Na_2_SO_4_, and the solvents were evaporated to dryness to obtain the crude product, which was used directly without any purification. **Step 2:** A mixture of the crude *sec*-amine (5 mmol) and K_2_CO_3_ (1.38 g, 2 equiv.) in methyl iodide (10 mL) was stirred and heated at reflux for 3 d. Excess methyl iodide was removed under reduced pressure and the product was purified by column chromatography (silica gel; CH_2_Cl_2_/MeOH, 40:1→10:1) to obtain **7d** in 37% yield (two steps) (25% recovered *tert*-amine intermediate was also obtained that could be methylated again). ${\left[\alpha \right]}_{D}^{23}$ = −5.2 (*c* = 1.0, CH_2_Cl_2_). ^1^H NMR (300 MHz, CDCl_3_, 298 K): *δ* = 1.25-1.38 (m, 1 H), 1.45 (s, 9 H), 1.58-1.77 (m, 3 H), 1.85-2.08 (m, 3 H), 2.52-2.64 (m, 1 H), 2.99 (s, 3 H), 3.14 (s, 3 H), 4.14-4.28 (m, 1 H), 5.07-5.20 (m, 1 H), 5.30 (d, *J* = 13.4 Hz, 1 H), 5.47 (d, *J* = 13.4 Hz, 1 H), 6.16 (d, *J* = 10.1 Hz, 1 H), 7.40-7.53 (m, 2 H), 7.54-7.63 (m, 1 H), 7.67 (d, *J* = 7.0 Hz, 1 H), 7.86 (d, *J* = 8.0 Hz, 1 H), 7.94 (d, *J* = 8.3 Hz, 1 H), 8.22 (d, *J* = 8.2 Hz, 1 H) ppm. ^13^C NMR (75 MHz, CDCl_3_, 298 K): *δ* = 24.5, 24.6, 27.6, 28.4, 35.6, 48.8, 50.6, 51.6, 62.3, 76.5, 80.7, 123.3, 123.6, 125.0, 126.6, 128.1, 129.3, 132.0, 133.1, 134.0, 134.1, 155.6 ppm. IR (film): $\tilde{\nu }$ = 3439, 3244, 3005, 2976, 2936, 2864, 1697, 1508, 1489, 1456, 1393, 1366, 1321, 1273, 1242, 1159, 1047, 1024, 870, 808, 783, 733 cm^−1^. HRMS (ESI): *m*/*z* calcd. for C_24_H_35_N_2_O_2_^+^ [M^+^] 383.2699; found 383.2693.

#### Catalyst 1o

**Step 1:** A solution of quaternary ammonium salt **7d** (105 mg, 0.2 mmol) and trifluoroacetic acid (155 μL, 10 equiv.) in CH_2_Cl_2_ (2 mL) was stirred at room temp. for 2 h. After evaporation to dryness, the crude amine was directly subjected to the final coupling step (the reaction could also be carried out by using aq. HI). **Step 2:** A mixture of amine, 3,5-dimethoxycarbonyl isocyanate (70 mg, 1.5 equiv.), and K_2_CO_3_ (83 mg, 3 equiv.) in CH_2_Cl_2_(2 mL) was stirred at room temp. for 18 h. After filtration and evaporation to dryness, the crude product was purified by column chromatography (CH_2_Cl_2_/MeOH, 50:1→10:1) to obtain catalyst **1o** (74 mg, 57%; two steps) as a yellowish oil. ${\left[\alpha \right]}_{D}^{23}$ = −49.1 (*c* = 0.75, CH_2_Cl_2_). ^1^H NMR (300 MHz, CDCl_3_, 298 K): *δ* = 1.25-1.48 (m, 2 H), 1.62-1.90 (m, 2 H), 1.91-2.08 (m, 2 H), 2.15-2.28 (m, 1 H), 2.58-2.70 (m, 1 H), 2.90 (s, 3 H), 3.17 (s, 3 H), 3.94 (s, 6 H), 4.45-4.70 (m, 2 H), 5.42 (d, *J* = 13.1 Hz, 1 H), 5.70 (d, *J* = 13.1 Hz, 1 H), 7.15 (t, *J* = 7.6 Hz, 2 H), 7.24 (d, *J* = 7.8 Hz, 1 H), 7.45 (d, *J* = 7.1 Hz, 1 H), 7.66 (t, *J* = 8.6 Hz, 2 H), 7.97 (d, *J* = 9.7 Hz, 1 H), 8.08 (d, *J* = 8.6 Hz, 1 H), 8.42 (t, *J* = 1.4 Hz, 1 H), 8.56 (d, *J* = 1.4 Hz, 2 H), 9.05 (s, 1 H) ppm. ^13^C NMR (75 MHz, CDCl_3_, 298 K): *δ* = 24.5, 25.0, 27.4, 36.1, 48.0, 50.6, 51.4, 52.4, 63.5, 77.3, 122.9, 123.2, 123.8, 124.6, 125.0, 126.4, 127.8, 129.0, 131.2, 131.7, 132.8, 133.6, 133.9, 139.9, 155.2, 166.3 ppm. IR (film): $\tilde{\nu }$ = 3244, 3028, 2943, 2866, 1717, 1684, 1558, 1541, 1508, 1437, 1346, 1317, 1242, 1123, 1047, 997, 876, 808, 783, 754 cm^−1^. HRMS (ESI): *m*/*z* calcd. for C_30_H_36_N_3_O_5_^+^[M^+^] 518.2655; found 518.2662.

#### General Procedure for the Asymmetric α-Fluorination

Reactions were typically carried out by using 0.1–0.5 mmol of the keto ester. Aqueous K_3_PO_4_ (2 m, 2 equiv.) was added to a mixture of keto ester and catalyst **1o** (2 mol-%) in *m*-xylene (20 mL/mmol keto ester) and the mixture was cooled to −10 °C. NFSI was added portionwise over 2 h and the mixture was vigorously stirred for another 10 h at −10 °C under an Ar atmosphere. The reaction was quenched by addition of satd. NH_4_Cl and the mixture was extracted with CH_2_Cl_2_. After drying over Na_2_SO_4_ and evaporation to dryness, the product was purified by silica gel column chromatography (heptanes/EtOAc, 20:1) to give the products in the reported yields.

#### Compound (*R*)-19a

Reacting **17a** (112 mg, 0.48 mmol) with NFSI (**18**) gave the title compound. Analytical data are in full accordance with those reported.^[25]^ Yield 95%; 92:8 *er*; ${\left[\alpha \right]}_{D}^{20}$ = +3.2 (*c* = 0.65, CHCl_3_). ^1^H NMR (700 MHz, CDCl_3_, 298 K): *δ* = 1.41 (s, 9 H), 3.38 (dd, *J* = 22.8, 17.6 Hz, 1 H), 3.71 (dd, *J* = 17.6, 10.9 Hz, 1 H), 7.44 (t, *J* = 6.8 Hz, 1 H), 7.48 (d, *J* = 7.4 Hz, 1 H), 7.67 (t, *J* = 7.6 Hz, 1 H), 7.81 (d, *J* = 7.7 Hz, 1 H) ppm. ^13^C NMR (175 MHz, CDCl_3_, 298 K): *δ* = 27.9, 38.4 (d, *J* = 24.1 Hz), 84.2, 94.5 (d, *J* = 202.4 Hz), 125.5, 126.6, 128.6, 133.7, 136.6, 151.1 (d, *J* = 3.5 Hz), 166.4 (d, *J* = 27.9 Hz), 195.9 (d, *J* = 17.9 Hz) ppm. ^19^F NMR (282 MHz, CDCl_3_, 298 K): *δ* = −164.0 (dd, *J* = 22.8, 10.9 Hz) ppm. IR (film): $\tilde{\nu }$ = 3003, 2981, 2936, 1753, 1717, 1607, 1466, 1370, 1296, 1209, 1152, 1074, 924, 835, 746, 723 cm^−1^. HRMS (ESI): *m*/*z* calcd. for C_14_H_15_FO_3_ [M + NH_4_]^+^ 268.13435; found 268.13488. The enantioselectivity was determined by HPLC analysis {Chiralpak AD-H; hexane/*i*PrOH, 200:1; 0.75 mL/min; 10 °C; *t*_R_ = 25.0 [(*S*)-enantiomer], 32.2 [(*R*)-enantiomer] min}.

## Supplementary Material

Supplementary Information General information, syntheses of bifunctional ammonium salts, asymmetric α-fluorination, copies of NMR spectra of key intermediates and most relevant catalysts, copies of NMR spectra of selected known compounds and of new fluorination products, HPLC chromatograms (chiral stationary phase).

## Figures and Tables

**Table 1:** Identification of the most active catalyst and the best-suited reaction conditions for the asymmetric α-fluorination of β-keto ester **17a**. 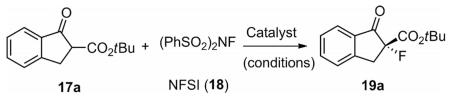

Entry^[a]^	Cat. (%)	Solvent	Base (equiv.)	*T* (°C)	*e.r.*^[b]^ (*R/S*)^[c]^
1	**1a** (5)	toluene	K_2_CO_3_ (0.5 m) (1.1)	0	77:23
2	**1b** (5)	toluene	K_2_CO_3_ (0.5 m) (1.1)	0	58:42
3	**1c** (5)	toluene	K_2_CO_3_ (0.5 m) (1.1)	0	80:20
4^[d]^	**1c** (5)	toluene	K_2_CO_3_ (0.5 m) (0.05)	0	52:48
5	**1c** (5)	toluene	K_2_CO_3_ (s) (1.1)	0	75:25
6	**2a** (5)	toluene	K_2_CO_3_ (0.5 m) (1.1)	0	34:66
7	**3a** (5)	toluene	K_2_CO_3_ (0.5 m) (1.1)	0	67:33
8	**1s** (5)	toluene	K_2_CO_3_ (0.5 m) (1.1)	0	42:58
9	**1q** (5)	toluene	K_2_CO_3_ (0.5 m) (1.1)	0	35:65
10	**4a** (5)	toluene	K_2_CO_3_ (0.5 m) (1.1)	0	40:60
11	**4b** (5)	toluene	K_2_CO_3_ (0.5 m) (1.1)	0	23:77
12	**1c** (5)	toluene	KOH (0.5 m) (1.1)	0	78:22
13	**1c** (5)	toluene	LiOH (0.5 m) (1.1)	0	51:49
14	**1c** (5)	toluene	K_2_HPO_4_ (0.5 m) (2)	0	59:41
15^[e]^	**1c** (5)	toluene	K_3_PO_4_ (0.5 m) (1.1)	0	81:19
16	**1c** (20)	toluene	K_3_PO_4_ (0.5 m) (2)	0	82:18
17	**1c** (2)	toluene	K_3_PO_4_ (0.5 m) (2)	0	81:19
18	**1c** (1)	toluene	K_3_PO_4_ (0.5 m) (2)	0	81:19
19	**1c** (2)	CH_2_Cl_2_	K_3_PO_4_ (0.5 m) (2)	0	69:31
20	**1c** (2)	cyclohexane	K_3_PO_4_ (0.5 m) (2)	0	52:48
21	**1c** (2)	MTBE	K_3_PO_4_ (0.5 m) (2)	0	78:22
22	**1c** (2)	fluorobenzene	K_3_PO_4_ (0.5 m) (2)	0	76:24
23	**1c** (2)	mesitylene	K_3_PO_4_ (0.5 m) (2)	0	81:19
24	**1c** (2)	*m*-xylene	K_3_PO_4_ (0.5 m) (2)	0	82:18
25	**1d** (2)	*m*-xylene	K_3_PO_4_ (0.5 m) (2)	0	79:21
26	**1p** (2)	*m*-xylene	K_3_PO_4_ (0.5 m) (2)	0	20:80
27	**1g** (2)	*m*-xylene	K_3_PO_4_ (0.5 m) (2)	0	75:25
28	**1e** (2)	*m*-xylene	K_3_PO_4_ (0.5 m) (2)	0	82:18
29	**1f** (2)	*m*-xylene	K_3_PO_4_ (0.5 m) (2)	0	85:15
30	**1h** (2)	*m*-xylene	K_3_PO_4_ (0.5 m) (2)	0	79:21
31	**1i** (2)	*m*-xylene	K_3_PO_4_ (0.5 m) (2)	0	80:20
32	**1j** (2)	*m*-xylene	K_3_PO_4_ (0.5 m) (2)	0	86:14
33	**1k** (2)	*m*-xylene	K_3_PO_4_ (0.5 m) (2)	0	90:10
34	**1l** (2)	*m*-xylene	K_3_PO_4_ (0.5 m) (2)	0	89:11
35	**1r** (2)	*m*-xylene	K_3_PO_4_ (0.5 m) (2)	0	15:85
36	**4c** (2)	*m*-xylene	K_3_PO_4_ (0.5 m) (2)	0	43:57
37	**1m** (2)	*m*-xylene	K_3_PO_4_ (0.5 m) (2)	0	85:15
38	**1n** (2)	*m*-xylene	K_3_PO_4_ (0.5 m) (2)	0	86:14
39	**1o** (2)	*m*-xylene	K_3_PO_4_ (0.5 m) (2)	0	91:9
40^[f],[g]^	**1o** (2)	*m*-xylene	K_3_PO_4_ (2 m) (2)	−10	92:8
41^[g]^	**1o** (2)	*m*-xylene	K_3_PO_4_ (2 m) (2)	−20	88:12

[a] All reactions were run for 5 h (ensuring complete conversion of **17a**) using **18** (1.1 equiv.). Different concentrations in the range 0.05–0.3 m did not affect conversion or selectivity.

[b] Determined by HPLC analysis by using a chiral stationary phase.

[c] Determined by comparison of the optical rotation and the retention order with reported values.^[25,26]^

[d] 70% conversion.

[e] The use of 0.5 equiv. base gave 73:27 *er* only.

[f] Partial freezing of the aqueous layer when using 0.5 m K_3_PO_4_.

[g] 12 h reaction time to ensure complete conversion.

**Table 2:** Application scope.^[a]^ 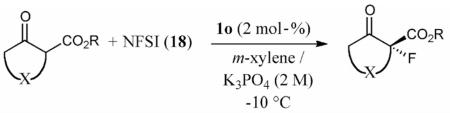

Entry	Keto ester		Yield [%]^[b]^	*e.r.* ^[b]^
1	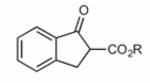	**17a** (R = *t*Bu)	95	92:8
2		**17b** (R = Me)	92	85:15 (>99:1)^[d]^
3		**17c** (R = Bn)	86	85:15
4		**17d** (R = CMe_2_Ph)	52^[e]^	89:11
5		**17e** (R = 1-Ad)^[f]^	96	93:7
6	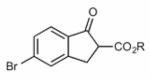	**22a** (R = *t*Bu)	73	88:12
7		**22b** (R = 1-Ad)^[f]^	85	89:11 (97:3)^[d]^
8	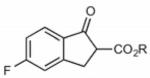	**24a** (R = *t*Bu)	75	91:9
9		**24b**(R=1-Ad)^[f]^	86	92:8
10	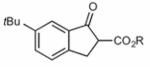	**26a** (R = *t*Bu)	79	85:15
11		**26b** (R = 1-Ad)^[f]^	96	86:14
12	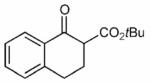	28	73	87:13
13	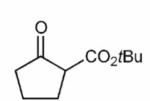	30	66	89:11 (82:18)^[g]^

[a] All reactions were carried out by using **18** (1.1 equiv.) for 12 h to ensure complete conversion.

[b] Isolated yields.

[c] Determined by HPLC analysis by using a chiral stationary phase.

[d] After a single recrystallization from heptane/EtOAc.

[e] The product partially hydrolyzed and decarboxylated during column chromatography.

[f] Ad = adamantyl.

[g] Using the ethyl ester.
