# Supplementary material for: Syntheses and Applications of (Thio)Urea-Containing Chiral Quaternary Ammonium Salt Catalysts
Source: European J Org Chem. Author manuscript; Available in PMC 2014 Oct 20. (PMC4202195; doi:10.1002/ejoc.201301594)
Supplement: Supplementary Information — General information, syntheses of bifunctional ammonium salts, asymmetric α-fluorination, copies of NMR spectra of key intermediates and most relevant catalysts, copies of NMR spectra of selected known compounds and of new fluorination products, HPLC chromatograms (chiral stationary phase). [file NIHMS60690-supplement-Supplementary_Information.pdf]

Eur. J. Org. Chem. **2014** · © WILEY-VCH Verlag GmbH & Co. KGaA, 69451 Weinheim, 2014 · ISSN 1099–0690

SUPPORTING INFORMATION

DOI: 10.1002/ejoc.201301594
Title: Syntheses and Applications of (Thio)Urea-Containing Chiral Quaternary Ammonium Salt Catalysts
Author(s): Johanna Novacek, Mario Waser*

1. General Information:..2
2. Syntheses of Bifunctional Ammonium Salts:...3
3. Asymmetric α-Fluorination:...20
4. Copies of NMR Spectra of Key-Intermediates and Most Relevant Catalysts: 26
5. Copies of NMR Spectra of Selected Known and of the New Fluorination Products:.....47
6. HPLC-Chromatograms (Chiral Stationary Phase): 56

1. General Information:

^{1}H- and ^{13}C-NMR spectra were recorded on a Bruker Avance III 300 MHz spectrometer and on a Bruker Avance III 700 MHz spectrometer with TCI cryoprobe. All NMR spectra were referenced on the solvent peak. High resolution mass spectra were obtained using an Agilent 6520 Q-TOF mass spectrometer with an ESI source and an Agilent G1607A coaxial sprayer. All analyses were made in the positive ionization mode. Purine (exact mass for $[M+H]^+$ = 121.050873) and 1,2,3,4,5,6-hexakis(2,2,3,3-tetrafluoropropoxy)-1,3,5,2,4,6-triazatriphosphinane (exact mass for $[M+H]^+$ = 922.009798) were used for internal mass calibration. IR spectra were recorded on a Shimadzu IR Affinity-1 fourier transform infrared spectrometer. Optical rotations were recorded on a Perkin Elmer Polarimeter Model 241 MC and on a Schmidt + Haensch Polarimeter Model UniPol L 1000. HPLC was performed using a Dionex Summit HPLC system with a Chiralcel OD-H (250 x 4.6 mm), a Chiralcel OD-R (250 x 4.6 mm, 10 µm), or a Chiralpak AD-H (250 x 4.6 mm, 5 µm) chiral stationary phase. All chemicals were purchased from commercial suppliers and used without further purification unless otherwise stated. All reactions were performed under an Ar-atmosphere. Starting β-ketoesters were either purchased from commercial suppliers or prepared according to literature-known methods.[1]

1) a) T. A. Moss, D. R. Fenwick, D. J. Dixon *J. Am. Chem. Soc.* **2008**, *130*, 10076-10077; b) D. Y. Kim, E. J. Park, *Org. Lett.* **2002**, *4*, 545-547; c) X. Wang, Q. Lan, S. Shirakawa, K. Maruoka, *Chem. Commun.* **2010**, *46*, 321-323; d) E.-M. Tanzer, W. B. Schweizer, M.-O. Ebert, R. Gilmour, *Chem. Eur. J.* **2012**, *18*, 2006-2013; e) M. Lian, J. Du, Q. Meng, Z. Gao *Eur. J. Org. Chem.* **2010**, 6525-6530.

2. Syntheses of Bifunctional Ammonium Salts:

Syntheses of Cyclohexanediamine-Based Catalysts 1a – 1o:

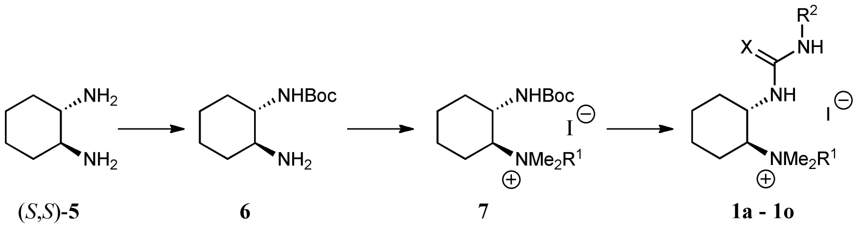

General Syntheses of 7 ($R^1 \neq$ Me): *Step 1:* The corresponding benzaldehyde (3 mmol) was added to a solution of **6** (3 mmol) (prepared from (*S*,*S*)-cyclohexanediamine-**5**-dihydrochloride[2] according to literature[3]) in THF:MeOH = 1:1 (12 mL) and the solution was stirred at r.t. for 2 h. After the addition of 1.5 eq $NaBH_4$ stirring was continued for another 2 h at r.t.. The reaction was quenched by addition of H_2O and extracted with H_2O/Et_2O. The organic phase was washed with brine, dried over Na_2SO_4, and evaporated to dryness to obtain the crude product which could be directly used without any purification. *Step 2:* A mixture of the crude *sec*-amine (3 mmol) and K_2CO_3 (2 eq.) in 3 ml methyl iodide was stirred at reflux for 3 d. Excess methyl iodide was removed under reduced pressure and the product was purified by column chromatography (silica gel, DCM:MeOH, 40:1 → 10:1) to obtain compounds **7** in the reported yields.

Compound 7a: Obtained in 64% (two steps, 3 mmol scale) as an oily residue. $[\alpha]_D^{22}$ (c = 7.5, DCM) = -2.0°; ^{1}H NMR (700 MHz, δ, $CDCl_3$, 298 K): 1.27-1.36 (m, 1H), 1.44 (s, 9H), 1.52-1.65 (m, 2H), 1.71-1.77 (m, 1H), 1.87-1.94 (m, 1H), 1.95-2.02 (m, 2H), 2.51-2.56 (m, 1H), 3.06 (s, 3H), 3.19 (s, 3H), 4.08-4.15 (m, 1H), 4.82 (d, 1H, *J* = 12.4 Hz), 4.82-4.89 (m, 1H), 4.94 (d, 1H, *J* = 12.4 Hz), 6.02 (d, 1H, *J* = 9.3 Hz), 7.40-7.49 (m, 5H) ppm; ^{13}C NMR (176 MHz, δ, $CDCl_3$, 298 K): 24.6, 24.7, 27.5, 28.5, 35.6, 49.2, 50.5, 51.5, 65.8, 76.3, 80.9, 127.2, 129.4, 131.0, 133.4, 155.6 ppm; IR (film): $\tilde{\nu}$ = 3435, 3237, 2976, 2936, 2864, 1691, 1510, 1450, 1366, 1321, 1275, 1240, 1157, 1045, 1024, 993, 914, 851, 775 cm^{-1}; HRMS (ESI) *m/z* calcd for $C_{20}H_{33}N_2O_2^+$: 333.2537 [M^+], found: 333.2539.

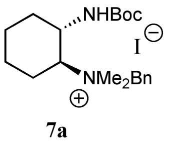

7a

---

2) H.-J. Schanz, M. Linseis, D. Gilheany, *Tetrahedron: Asymmetry* **2003**, *14*, 2763-2769.
3) D. W: Lee, H.-J. Ha, W. K. Lee, *Synth. Commun.* **2007**, *37*, 737-742.

Compound 7b: Obtained in 24% (plus 33% isolated tert. amine intermediate that could be resubmitted again) (two steps, 1 mmol scale) as an oily residue. $[\alpha]_D^{22}$ (c = 0.33, DCM) = -2.7°; ^{1}H NMR (300 MHz, δ, $CDCl_3$, 298 K): 1.27-1.42 (m, 1H), 1.48 (s, 9H), 1.56-1.65 (m, 2H), 1.76-1.88 (m, 1H), 1.91-2.10 (m, 3H), 2.57-2.66 (m, 1H), 3.20 (s, 3H), 3.28 (s, 3H), 4.06-4.20 (m, 1H), 4.91-5.04 (m, 1H), 5.10 (d, 1H, *J* = 12.4 Hz), 5.34 (d, 1H, *J* = 12.9 Hz), 5.84 (d, 1H, *J* = 10.0 Hz), 7.73 (t, 1H, *J* = 8.0 Hz), 8.14 (d, 1H, *J* = 7.8 Hz), 8.31-8.40 (m, 2H) ppm; ^{13}C NMR (75 MHz, δ, $CDCl_3$, 298 K): 24.7, 24.7, 27.5, 28.5, 35.6, 49.2, 50.4, 51.6, 64.5, 77.4, 81.5, 125.9, 127.7, 129.4, 130.9, 139.8, 148.6, 155.7 ppm; IR (film): $\tilde{\nu}$ = 2976, 2936, 2866, 1697, 1533, 1508, 1456, 1352, 1321, 1279, 1242, 1163, 1047, 1024, 768, 731 cm^{-1}; HRMS (ESI) *m/z* calcd for $C_{20}H_{32}N_3O_4^+$: 378.2393 [M^+], found: 378.2395.

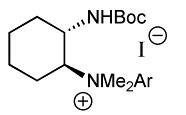

7b (Ar = 3-NO_2-Bn)

Compound 7c: Obtained in 42% (two steps, 1 mmol scale) as an oily residue. $[\alpha]_D^{22}$ (c = 1.1, DCM) = -0.2°; ^{1}H NMR (300 MHz, δ, $CDCl_3$, 298 K): 1.23-1.40 (m, 1H), 1.48 (s, 9H), 1.49-1.85 (m, 3H), 1.89-2.08 (m, 3H), 2.50-2.59 (m, 1H), 3.06 (s, 3H), 3.19 (s, 3H), 3.85 (s, 3H), 4.05-4.20 (m, 1H), 4.77 (d, 1H, *J* = 12.4 Hz), 4.90-5.02 (m, 1H), 4.92 (d, 1H, *J* = 12.4 Hz), 5.94 (d, 1H, *J* = 10.2 Hz), 6.96 (d, 2H, *J* = 8.6 Hz), 7.41 (d, 2H, *J* = 8.6 Hz) ppm; ^{13}C NMR (75 MHz, δ, $CDCl_3$, 298 K): 24.6, 27.3, 28.4, 35.4, 48.6, 49.8, 51.4, 55.5, 65.6, 75.6, 80.8, 114.7, 118.7, 134.6, 155.4, 161.4 ppm; IR (film): $\tilde{\nu}$ = 3447, 3258, 2976, 2934, 2864, 2839, 1690, 1611, 1514, 1449, 1366, 1321, 1283, 1252, 1159, 1024, 916, 837, 768 cm^{-1}; HRMS (ESI) *m/z* calcd for $C_{21}H_{35}N_2O_3^+$: 363.2648 [M^+], found: 363.2656.

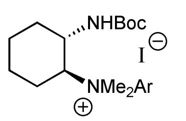

7c (Ar = 4-MeO-Bn)

Compound 7d: Obtained in 37% (+ 25% recovered tert.-amine intermediate that could be resubmitted to the methylation again) (two steps, 5 mmol scale) as an oily residue. $[\alpha]_D^{23}$ (c = 1.0, DCM) = -5.2°; ^{1}H NMR (300 MHz, δ, $CDCl_3$, 298 K): 1.25-1.38 (m, 1H), 1.45 (s, 9H), 1.58-1.77 (m, 3H), 1.85-2.08 (m, 3H), 2.52-2.64 (m, 1H), 2.99 (s, 3H), 3.14 (s, 3H), 4.14-4.28 (m, 1H), 5.07-5.20 (m, 1H), 5.30 (d, 1H, *J* = 13.4 Hz), 5.47 (d, 1H, *J* = 13.4 Hz), 6.16 (d, 1H, *J* = 10.1 Hz), 7.40-7.53 (m, 2H), 7.54-7.63 (m, 1H), 7.67 (d, 1H, *J* = 7.0 Hz), 7.86 (d, 1H, *J* = 8.0 Hz), 7.94 (d, 1H, *J* = 8.3 Hz), 8.22 (d, 1H, *J* = 8.2 Hz) ppm; ^{13}C NMR (75MHz, δ, $CDCl_3$, 298 K): 24.5, 24.6, 27.6, 28.4, 35.6, 48.8, 50.6, 51.6, 62.3, 76.5, 80.7, 123.3, 123.6, 125.0, 126.6, 128.1, 129.3, 132.0, 133.1, 134.0, 134.1, 155.6 ppm; IR (film): $\tilde{\nu}$ = 3439, 3244, 3005, 2976, 2936,

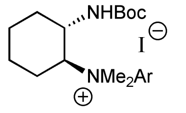

7d (Ar = α-Np-CH_2-)

2864, 1697, 1508, 1489, 1456, 1393, 1366, 1321, 1273, 1242, 1159, 1047, 1024, 870, 808, 783, 733 cm^{-1}; HRMS (ESI) *m/z* calcd for $C_{24}H_{35}N_2O_2^+$: 383.2699 [M^+], found: 383.2693.

Compound 7e: Obtained in 61% (two steps, 2 mmol scale) as an oily residue. $[\alpha]_D^{23}$ (c = 0.5, DCM) = -4.5°; ^{1}H NMR (300 MHz, δ, $CDCl_3$, 298 K): 1.24-1.50 (m, 2H), 1.47 (s, 9H), 1.56-1.67 (m, 2H), 1.67-1.79 (m, 1H), 1.86-2.13 (m, 2H), 2.51-2.64 (m, 1H), 3.11 (s, 3H), 3.22 (s, 3H), 4.06-4.23 (m, 1H), 4.82-4.94 (m, 1H), 5.00 (d, 1H, *J* = 12.6 Hz), 5.11 (d, 1H, *J* = 12.6 Hz), 6.14 (d, 1H, *J* = 9.9 Hz), 7.48-7.59 (m, 3H), 7.80-7.88 (m, 3H), 8.02 (s, 1H) ppm; ^{13}C NMR (75 MHz, δ, $CDCl_3$, 298 K): 24.7, 24.8, 27.5, 28.5, 35.5, 49.3, 50.4, 51.5, 65.9, 76.4, 80.9, 124.5, 127.3, 127.8, 128.0, 128.4, 129.2 (2x), 132.9, 133.9, 134.0, 155.6 ppm; IR (film): $\tilde{\nu}$ = 3237, 2934, 2862, 1701, 1508, 1450, 1391, 1366, 1321, 1277, 1242, 1167, 1020 cm^{-1}; HRMS (ESI) *m/z* calcd for $C_{24}H_{35}N_2O_2^+$: 383.2699 [M^+], found: 383.2698.

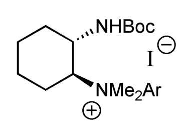

7e (Ar = β-Np-CH_2-)

Synthesis of 7f: K_2CO_3 (319 mg, 2.31 mmol, 2 eq) was added to a solution of mono-Boc-protected diamine **6** (247 mg, 1.16 mmol) in 5 ml AcN. After the addition of 360 µl (5.78 mmol, 5 eq) methyl iodide the suspension was stirred for 3 d. After evaporation of excess methyl iodide and AcN, the residue was dissolved in DCM and filtered to give **7f** as an oily residue in quantitative yield. The product was used without further purification. $[\alpha]_D^{22}$ (c = 6.9, DCM) = +8.3°; ^{1}H NMR (300 MHz, δ, $CDCl_3$, 298 K): 1.20-1.46 (m, 2H), 1.43 (s, 9H) 1.50-1.66 (m, 1H), 1.70-1.80 (m, 1H), 1.85-1.98 (m, 3H), 2.33-2.44 (m, 1H), 3.42 (s, 9H), 3.86-4.01 (m, 1H), 4.65 (m, 1H), 5.76 (d, 1H, NH) ppm; ^{13}C NMR (75 MHz, δ, $CDCl_3$, 298 K): 24.6, 24.7, 27.2, 28.5, 35.2, 51.4, 75.0, 81.0, 155.5 ppm; IR (film): $\tilde{\nu}$ = 3443, 3244, 3005, 2974, 2940, 2862, 1690, 1506, 1449, 1390, 1365, 1321, 1277, 1248, 1159, 1107, 1045, 1022, 960, 932, 868, 847 cm^{-1}; HRMS (ESI) *m/z* calcd for $C_{14}H_{29}N_2O_2^+$: 257.2224 [M^+], found: 257.2226.

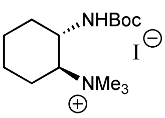

7f

General Syntheses of Catalysts 1a – 1o: *Step 1:* A solution of the quaternary ammonium salt **7** and trifluoroacetic acid (10 eq.) in DCM (10 mL / mmol) was stirred at r.t. for at least 2 h. After evaporation to dryness, the crude amine was directly subjected to the final coupling step (the reaction can also be carried out using aqueous HI). *Step 2:* A mixture of the amine, R^2NCX (1.5 eq.), and K_2CO_3 (3 eq.) in DCM (10 mL / mmol) was stirred at r.t. for 8-18 h. After filtration and evaporation to dryness, the crude product was purified by column chromatography (DCM:MeOH, 50:1 → 10:1) to obtain catalysts **1** in the reported yields.

Compound 1a. Obtained in 71% (2 steps, 0.5 mmol scale) as a colourless oil. $[\alpha]_D^{23}$ (c = 0.9, DCM) = -30.9°; ^{1}H NMR (300 MHz, δ, $CDCl_3$, 298 K): 1.15-1.32 (m, 1H), 1.36-1.53 (m, 2H), 1.59-1.75 (m, 2H), 1.79-1.90 (m, 1H), 1.94-2.05 (m, 1H), 2.28-2.38 (m, 1H), 2.86 (s, 3H), 3.00 (s, 3H), 3.84-3.95 (m, 1H), 4.15-4.29 (m, 1H), 4.67-4.78 (m, 2H), 6.90 (t, 1H, *J* = 7.6 Hz), 7.17 (dd, 2H, J_1 = 7.9 Hz, J_2 = 7.6 Hz), 7.26-7.40 (m, 5H), 7.47 (d, 2H, *J* = 7.9 Hz), 7.84 (d, 1H, *J* = 9.8 Hz), 9.06 (s, 1H) ppm; ^{13}C NMR (75 MHz, δ, $CDCl_3$, 298 K): 24.6, 25.2, 27.2, 35.7, 48.8, 50.4, 50.9, 66.5, 77.4, 119.1, 122.5, 127.1, 129.0, 129.6, 131.1, 133.3, 139.9, 155.6 ppm; IR (film): $\bar{\nu}$ = 3262, 3190, 3038, 2941, 2865, 1670, 1597, 1549, 1499, 1443, 1321, 1258, 1202, 1130, 800, 733 cm^{-1}; HRMS (ESI): *m/z* calcd for $C_{22}H_{30}N_3O^+$: 352.2383 [M^+]; found: 352.2392.

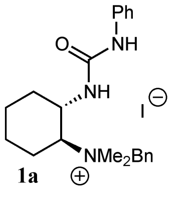

1a

Compound 1b. Obtained in 50% (2 steps, 0.1 mmol scale) as a yellowish oil. $[\alpha]_D^{23}$ (c = 0.3, DCM) = -19.4°; ^{1}H NMR (300 MHz, δ, $CDCl_3$, 298 K): 1.28-1.45 (m, 1H), 1.50-1.69 (m, 2H), 1.73-1.87 (m, 2H), 1.91-2.10 (m, 1H), 2.14-2.25 (m, 1H), 2.46-2.60 (m, 1H), 3.08 (s, 3H), 3.20 (s, 3H), 4.35-4.47 (m, 1H), 4.69 (d, 1H, *J* = 12.6 Hz), 5.10 (d, 1H, *J* = 12.6 Hz), 5.20-5.35 (m, 1H), 7.15 (t, 1H, *J* = 7.4 Hz), 7.31 (dd, 2H, J_1 = 7.7 Hz, J_2 = 7.4 Hz), 7.37-7.51 (m, 5H), 7.60 (d, 2H, *J* = 7.7 Hz), 8.81 (d, 1H, *J* = 9.6 Hz), 9.24 (s, 1H) ppm; ^{13}C NMR (75 MHz, δ, $CDCl_3$, 298 K): 24.3, 24.9, 27.3, 35.0, 50.0, 51.1, 54.0, 65.9, 77.7, 124.4, 125.8, 126.9, 128.8, 129.5, 131.1, 133.3, 138.6, 180.2 ppm; IR (film): $\bar{\nu}$ = 3206, 3032, 2936, 2860, 1599, 1533, 14497, 1449, 1348, 1321, 1265, 1215, 1155, 1134, 1028, 982 cm^{-1}; HRMS (ESI): *m/z* calcd for $C_{22}H_{30}N_3S^+$: 368.2155 [M^+]; found: 368.2152.

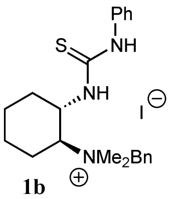

1b

Compound 1c. Obtained in 70% (2 steps, 2 mmol scale) as a yellowish oil. $[\alpha]_D^{23}$ (c = 0.5, DCM) = -47.9°; ^{1}H NMR (300 MHz, δ, $CDCl_3$, 298 K): 1.25-1.44 (m, 1H), 1.51-1.71 (m, 2H), 1.73-1.88 (m, 2H), 1.91-2.05 (m, 1H), 2.08-2.19 (m, 1H), 2.49-2.60 (m, 1H), 3.05 (s, 3H), 3.22 (s, 3H), 4.25-4.50 (m, 2H), 4.94 (d, 1H, *J* = 12.7 Hz), 5.00 (d, 1H, *J* = 12.7 Hz), 7.32-7.54 (m, 7H), 7.72 (dd, 1H, J_1 = 8.0, J_2 = 1.3 Hz), 7.83 (dd, 1H, J_1 = 8.1, J_2 = 2.0 Hz), 8.74 (t, 1H, *J* = 2.1 Hz), 9.21 (s, 1H) ppm; ^{13}C NMR (75 MHz, δ, $CDCl_3$, 298 K): 24.4, 24.9, 27.2, 35.8, 48.7, 50.4, 50.7, 66.8, 77.2, 113.1, 117.1, 124.3, 126.8, 129.4, 129.5, 131.0, 133.2, 140.6, 148.6, 154.9 ppm; IR (film): $\bar{\nu}$ = 3252, 3190, 3061, 3034, 2936, 2860, 1686, 1597, 1543, 1522, 1481, 1449, 1342, 1321, 1258, 1204, 1080, 733 cm^{-1}; HRMS (ESI): *m/z* calcd for $C_{22}H_{29}N_4O_3^+$: 397.2240 [M^+]; found: 397.2242.

1c
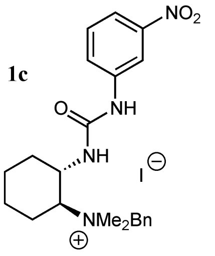

Compound 1d. Obtained in 48% (2 steps, 0.15 mmol scale) as a slightly yellow residue.

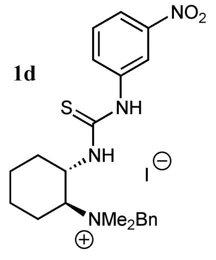

1d

$[\alpha]_D^{22}$ (c = 0.35, DCM) = -35.7°; ^{1}H NMR (700 MHz, δ, $CDCl_3$, 298 K): 1.35-1.47 (m, 1H), 1.62-1.70 (m, 2H), 1.83-1.92 (m, 2H), 2.01-2.08 (m, 1H), 2.24-2.29 (m, 1H), 2.51-2.57 (m, 1H), 3.11 (s, 3H), 3.23 (s, 3H), 4.31-4.38 (m, 1H), 4.65 (d, 1H, *J* = 12.6 Hz), 5.09 (d, 1H, *J* = 12.6 Hz), 5.27-5.33 (m, 1H), 7.39-7.55 (m, 6H), 7.86 (d, 1H, *J* = 8.1 Hz), 7.98 (d, 1H, *J* = 8.0 Hz), 8.93 (s, 1H), 9.37 (d, 1H, *J* = 9.9 Hz), 9.80 (s, 1H) ppm; ^{13}C NMR (176 MHz, δ, $CDCl_3$, 298 K): 24.2, 24.9, 27.1, 34.9, 50.1, 51.3, 53.6, 66.2, 78.0, 118.4, 119.7, 126.5, 129.2, 129.3, 129.7, 131.4, 133.2, 140.2, 148.1, 179.9 ppm; IR (film): $\tilde{\nu}$ = 3200, 3030, 2943, 2864, 1526, 1474, 1348, 1265, 1217, 991, 785, 735 cm^{-1}; HRMS (ESI): *m/z* calcd for $C_{22}H_{29}N_4O_2S^+$: 413.2011 [M$^+$]; found: 413.2014.

Compound 1e. Obtained in 47% (2 steps, 0.2 mmol scale) as a yellowish oil. $[\alpha]_D^{23}$ (c = 0.9, DCM) = -16.6°; ^{1}H NMR (300 MHz, δ, $CDCl_3$, 298 K): 1.31-1.45 (m, 1H), 1.50-1.65 (m, 2H), 1.72-1.93 (m, 2H), 1.95-2.05 (m, 1H), 2.10-2.22 (m, 1H), 2.50-2.61 (m, 1H), 3.04 (s, 3H), 3.23 (s, 3H), 4.25-4.45 (m, 2H), 4.95 (d, 1H, *J* = 12.7 Hz), 5.07 (d, 1H, *J* = 12.7 Hz), 7.25-7.45 (m, 7H), 7.56 (d, 2H, *J* = 9.5 Hz), 7.64 (d, 1H, *J* = 8.1 Hz), 8.10 (s, 1H), 8.88 (s, 1H) ppm; ^{13}C NMR (75 MHz, δ, $CDCl_3$, 298 K): 24.5, 24.9, 27.3, 35.8, 48.6, 50.5, 50.7, 66.9, 77.3, 115.2, 119.0, 126.8, 129.2, 129.4, 130.9, 133.2, 139.8, 155.0 ppm; IR (film): $\tilde{\nu}$ = 3264, 3208, 3088, 3063, 3034, 2943, 2864, 1684, 1558, 1541, 1447, 1339, 1319, 1260, 1215, 1165, 1123, 1069, 791 cm^{-1}; HRMS (ESI): *m/z* calcd for $C_{23}H_{29}F_3N_3O^+$: 420.2263 [M$^+$]; found: 420.2269.

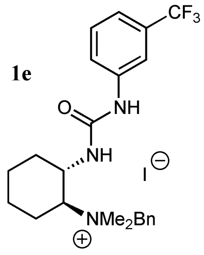

1e

Compound 1f. Obtained in 62% (2 steps, 0.2 mmol scale) as a yellow oil. $[\alpha]_D^{23}$ (c = 1.0, DCM) = -24.9°; ^{1}H NMR (300 MHz, δ, $CDCl_3$, 298 K): 1.22-1.33 (m, 1H), 1.36 (t, 3H, *J* = 7.2 Hz), 1.52-1.69 (m, 2H), 1.70-1.89 (m, 2H), 1.90-1.97 (m, 1H), 2.06-2.18 (m, 1H), 2.50-2.61 (m, 1H), 3.05 (s, 3H), 3.23 (s, 3H), 4.15-4.30 (m, 1H), 4.36 (q, 2H, *J* = 7.2 Hz), 4.38-4.52 (m, 1H), 4.95 (d, 1H, *J* = 12.3 Hz), 5.07 (d, 1H, *J* = 12.3 Hz), 7.26-7.55 (m, 7H), 7.64-7.74 (m, 2H), 8.39 (t, 1H, *J* = 1.7 Hz), 8.73 (s, 1H) ppm; ^{13}C NMR (75 MHz, δ, $CDCl_3$, 298 K): 14.4, 24.4, 25.0, 27.2, 35.9, 48.8, 50.4, 50.9, 60.9, 66.6, 77.1, 119.6, 123.3, 137.7, 127.0, 128.7, 129.3, 130.8, 131.0, 133.2, 139.4, 155.0, 166.7 ppm; IR (film): $\tilde{\nu}$ = 3456, 3256, 3192, 3065, 2936, 2862, 1686, 1593, 1549, 1481, 1439, 1366, 1321, 1287, 1204, 1103, 1022, 851, 756, 727 cm^{-1}; HRMS (ESI): *m/z* calcd for $C_{25}H_{34}N_3O_3^+$: 424.2600 [M$^+$]; found: 424.2604.

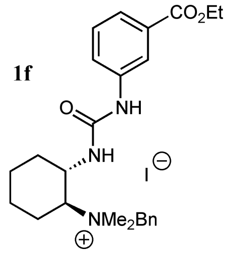

1f

Compound 1g. Obtained in 43% (2 steps, 0.1 mmol scale) as a yellowish oil. $[\alpha]_D^{23}$ (c = 0.9, DCM) = +8.0°; ^{1}H NMR (300 MHz, δ, $CDCl_3$, 298 K): 1.20-1.60 (m, 3H), 1.64-1.80 (m, 2H), 1.86-1.97 (m, 1H), 2.00-2.11 (m, 1H), 2.28-2.41 (m, 1H), 3.34 (s, 9H), 3.89 (td, 1H, J_1 = 11.0 Hz, J_2 = 3.2 Hz), 4.10-4.25 (m, 1H), 6.95-7.02 (m, 2H), 7.25 (m, 2H), 7.52 (d, 2H, J = 7.9 Hz), 8.38 (s, 1H) ppm; ^{13}C NMR (75 MHz, δ, $CDCl_3$, 298 K): 24.5, 24.8, 27.2, 35.7, 50.3, 54.4, 76.4, 119.1, 122.9, 129.0, 139.2, 155.0 ppm; IR (film): $\tilde{\nu}$ = 3258, 3188, 3130, 3032, 2938, 2861, 1682, 1595, 1541, 1497, 1441, 1319, 1256, 1223, 1204, 959, 843 cm^{-1}; HRMS (ESI): *m/z* calcd for $C_{16}H_{26}N_3O^+$: 276.2070 [M^+]; found: 276.2065.

1g

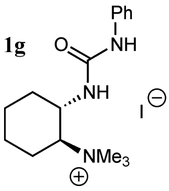

Compound 1h. Obtained in 70% (2 steps, 0.15 mmol scale) as a yellowish oil. $[\alpha]_D^{23}$ (c = 0.3, DCM) = -46.6°; ^{1}H NMR (300 MHz, δ, CD_3CN, 298 K): 1.38-1.52 (m, 2H), 1.65-1.85 (m, 3H), 2.00-2.16 (m, 2H), 2.20-2.38 (m, 1H), 3.06 (s, 3H), 3.09 (s, 3H), 3.70-3.81 (m, 1H), 4.32-4.48 (m, 1H), 4.81 (d, 1H, J = 12.8 Hz), 4.97 (d, 1H, J = 12.8 Hz), 6.95 (d, 1H, J = 8.7 Hz), 7.50 (t, 1H, J = 8.0 Hz), 7.42-7.55 (m, 3H), 8.01 (d, 1H, J = 7.7 Hz), 8.32-8.45 (m, 2H), 8.65 (s, 1H). 9.14 (s, 1H) ppm; ^{13}C NMR (75 MHz, δ, CD_3CN, 298 K): 23.4, 24.1, 25.7, 34.8, 49.2, 49.5, 50.6, 63.6, 76.8, 116.2, 122.2, 123.4, 124.9, 127.6, 129.2, 129.4, 129.6, 130.2, 139.1, 140.8, 148.2, 148.3, 154.4 ppm; IR (film): $\tilde{\nu}$ = 3252, 3055, 2932, 2859, 1686, 1599, 1522, 1481, 1348, 1258, 1206, 1084, 783, 733 cm^{-1}; HRMS (ESI): *m/z* calcd for $C_{22}H_{28}N_5O_5^+$: 442.2090 [M^+]; found: 442.2094.

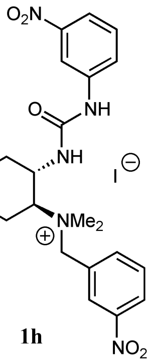

1h

Compound 1i. Obtained in 31% (2 steps, 0.2 mmol scale) as a yellowish oil. $[\alpha]_D^{23}$ (c = 0.5, DCM) = -35.1°; ^{1}H NMR (300 MHz, δ, $CDCl_3$, 298 K): 1.30-1.50 (m, 1H), 1.55-1.68 (m, 2H), 1.75-2.06 (m, 3H), 2.10-2.25 (m, 1H), 2.48-2.58 (m, 1H), 3.03 (s, 3H), 3.18 (s, 3H), 3.81 (s, 3H), 4.10-4.24 (m, 1H), 4.32-4.49 (m, 1H), 4.88 (d, 1H, J = 12.2 Hz), 4.94 (d, 1H, J = 12.2 Hz), 6.87 (d, 2H, J = 8.8 Hz), 7.33 (d, 2H, J = 8.8 Hz), 7.42 (t, 1H, J = 8.7 Hz), 7.63 (d, 1H, J = 9.5 Hz), 7.73 (dd, 1H, J_1 = 8.1 Hz, J_2 = 2.1 Hz), 7.87 (dd, 1H, J_1 = 8.2 Hz, J_2 = 2.1 Hz), 8.80 (d, 1H, J = 2.1 Hz), 9.16 (s, 1H) ppm; ^{13}C NMR (75 MHz, δ, $CDCl_3$, 298 K): 24.4, 24.9, 27.2, 35.5, 48.4, 50.4, 53.4, 55.4, 66.8, 77.2, 113.1, 114.8, 117.2, 118.3, 124.3, 134.5, 140.5, 148.7, 154.9, 161.5 ppm; IR (film): $\tilde{\nu}$ = 3252, 3028, 2934, 2862, 2837, 1886, 1611, 1524, 1516, 1476, 1341, 1254, 1204, 1180, 1028, 831, 735 cm^{-1}; HRMS (ESI): *m/z* calcd for $C_{23}H_{31}N_4O_4^+$: 427.2345 [M^+]; found: 427.2335.

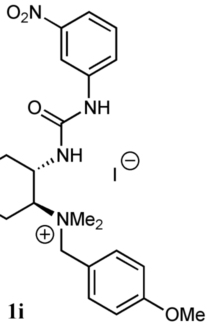

1i

Compound 1j. Obtained in 53% (2 steps, 0.15 mmol scale) as an oily residue. $[\alpha]_D^{23}$ (c = 0.45, DCM) = -45.5°; ^{1}H NMR (700 MHz, δ, $CDCl_3$, 298 K): 1.31-1.44 (m, 1H), 1.62-1.77 (m, 3H), 1.90-2.03 (m, 2H), 2.15-2.21 (m, 1H), 2.60-2.66 (m, 1H), 2.88 (s, 3H), 3.13 (s, 3H), 4.52-4.60 (m, 2H), 5.40 (d, 1H, *J* = 13.4 Hz), 5.65 (d, 1H, *J* = 13.4 Hz), 7.10-7.18 (m, 2H), 7.22-7.28 (m, 1H), 7.38-7.49 (m, 2H), 7.61-7.72 (m, 3H), 7.81 (dd, 1H, J_1 = 8.1 Hz, J_2 = 1.6 Hz), 7.89 (dd, 1H, J_1 = 8.3 Hz, J_2 = 1.8 Hz), 8.07 (d, 1H, *J* = 8.3 Hz), 8.88 (s, 1H), 9.36 (s, 1H) ppm; ^{13}C NMR (176 MHz, δ, $CDCl_3$, 298 K): 24.6, 25.1, 27.5, 36.2, 47.9, 50.7, 51.3, 63.8, 113.2, 117.4, 123.0, 123.3, 124.5, 124.8, 126.5, 127.9, 129.2, 129.6, 131.9, 132.9, 133.8, 134.0, 140.8, 148.8, 155.2 ppm; IR (film): $\bar{\nu}$ = 3250, 3057, 2943, 2860, 1685, 1597, 1545, 1528, 1483, 1352, 1207, 766 cm^{-1}; HRMS (ESI): *m/z* calcd for $C_{26}H_{31}N_4O_3^+$: 447.2396 [M^+]; found: 447.2405.

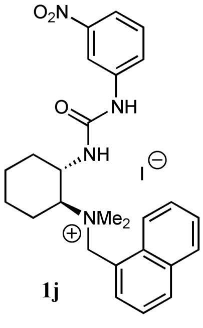

1j

Compound 1k. Obtained in 39% (2 steps, 0.12 mmol scale) as a yellowish oil. $[\alpha]_D^{22}$ (c = 0.45, DCM) = -55.5°; ^{1}H NMR (700 MHz, δ, $CDCl_3$, 298 K): 1.37 (t, 3H, *J* = 7.4 Hz), 1.35-1.42 (m, 1H), 1.65-1.74 (m, 2H), 1.76-1.83 (m, 1H), 1.93-2.05 (m, 2H), 2.18-2.24 (m, 1H), 2.58-2.64 (m, 1H), 2.88 (s, 3H), 3.15 (s, 3H), 4.37 (q, 2H, *J* = 7.4 Hz), 4.42-4.50 (m, 1H), 4.54-4.60 (m, 1H), 5.42 (d, 1H, *J* = 13.3 Hz), 5.63 (d, 1H, *J* = 13.3 Hz), 7.15-7.20 (m, 2H), 7.25-7.30 (m, 1H), 7.38 (t, 1H, *J* = 7.9 Hz), 7.46 (d, 1H, *J* = 6.6 Hz), 7.67 (d, 1H, *J* = 7.9 Hz), 7.71 (d, 1H, *J* = 8.1 Hz), 7.73-7.77 (m, 2H), 7.82 (d, 1H, *J* = 9.9 Hz), 8.07 (d, 1H, *J* = 8.4 Hz), 8.48 (s, 1H), 8.84 (s, 1H) ppm; ^{13}C NMR (176 MHz, δ, $CDCl_3$, 298 K): 14.5, 24.6, 25.1, 27.6, 36.2, 48.0, 50.6, 51.5, 61.1, 63.6, 77.5, 119.7, 123.1, 123.4, 123.5, 124.0, 124.7, 126.5, 128.0, 129.0, 129.1, 131.3, 131.8, 133.0, 133.7, 134.0, 139.7, 155.4, 166.8 ppm; IR (film): $\bar{\nu}$ = 3254, 3196, 3042, 2934, 2860, 1709, 1686, 1593, 1551, 1481, 1439, 1287, 1260, 1228, 1101, 1022, 806, 783, 758, 735 cm^{-1}; HRMS (ESI): *m/z* calcd for $C_{29}H_{36}N_3O_3^+$: 474.2757 [M^+]; found: 474.2762.

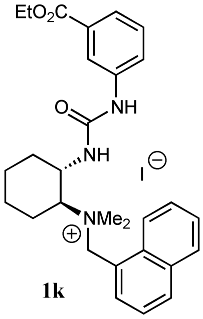

1k

Compound 1l. Obtained in 60% (2 steps, 0.2 mmol scale) as a yellowish oil. $[\alpha]_D^{23}$ (c = 1.0, DCM) = 36.6°; ^{1}H NMR (300 MHz, δ, $CDCl_3$, 298 K): 1.38 (t, 3H, *J* = 7.4 Hz), 1.55-1.98 (m, 6H), 2.08-2.22 (m, 1H), 2.51-2.61 (m, 1H), 3.03 (s, 3H), 3.23 (s, 3H), 4.20-4.33 (m, 1H), 4.38 (q, 2H, *J* = 7.4 Hz), 4.40-4.55 (m, 1H), 5.11 (d, 1H, *J* = 12.9 Hz), 5.27 (d, 1H, *J* = 12.9 Hz), 7.30-7.50 (m, 4H), 7.52-7.58 (m, 2H), 7.60-7.68 (m, 2H), 7.70-7.77 (m, 2H), 7.89 (s, 1H), 8.48 (s, 1H), 8.88 (s, 1H) ppm; ^{13}C NMR (75 MHz, δ, $CDCl_3$, 298 K): 14.4, 24.5, 25.0, 27.2, 35.9, 48.3, 50.5, 51.0, 61.0, 67.0, 77.3, 119.6, 123.3, 123.8, 124.1, 127.0, 127.6, 127.8, 128.2, 128.8, 128.9, 131.1, 132.6, 133.6, 133.7, 139.6, 155.1, 166.7 ppm; IR (film): $\tilde{\nu}$ = 3256, 3196, 3042, 2980, 2934, 2860, 1686, 1593, 1545, 1477, 1439, 1366, 1287, 1227, 1204, 1103, 1020, 914, 864, 820, 754, 727 cm^{-1}; HRMS (ESI): *m/z* calcd for $C_{29}H_{36}N_3O_3^+$: 474.2757 [M$^+$]; found: 474.2765.

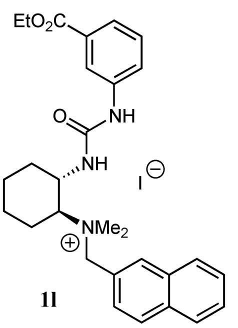

1l

Compound 1m. Obtained in 57% (2 steps, 0.1 mmol scale) as a yellowish oil. $[\alpha]_D^{23}$ (c = 0.4, DCM) = -28.6°; ^{1}H NMR (700 MHz, δ, $CDCl_3$, 298 K): 1.22-1.57 (m, 5H), 1.71-2.05 (m, 4H), 2.12-2.23 (m, 1H), 2.59-2.65 (m, 1H), 2.84 (s, 3H), 3.12 (s, 3H), 4.36 (q, *J* = 7.0 Hz, 2H), 4.41-4.50 (m, 1H), 4.53-4.63 (m, 1H), 5.42 (d, *J* = 13.3 Hz, 1H), 5.68 (d, *J* = 13.3 Hz, 1H), 7.07-7.19 (m, 2H), 7.36-7.46 (m, 1H), 7.61-7.71 (m, 3H), 7.72-7.83 (m, 3H), 8.00 (d, *J* = 8.3 Hz, 2H), 8.07 (d, *J* = 8.5 Hz, 1H), 9.19 (s, 1H) ppm; ^{13}C NMR (175 MHz, δ, $CDCl_3$, 298 K): 14.5, 24.65, 25.1, 27.7, 36.1, 47.4, 50.8, 51.0, 60.8, 64.3, 77.2, 117.7, 123.1, 123.4, 124.4, 124.7, 126.5, 128.0, 129.2, 130.9, 131.9, 133.0, 133.8, 134.0, 143.9, 155.1, 166.7 ppm; IR (film): $\tilde{\nu}$ = 3250, 3198, 3101, 3044, 2980, 2938, 2864, 1697, 1595, 1537, 1410, 1322, 1277, 1258, 1206, 1173, 1105, 1020, 857, 783 cm^{-1}; HRMS (ESI): *m/z* calcd for $C_{29}H_{36}N_3O_3^+$: 474.2757 [M$^+$]; found: 474.2767.

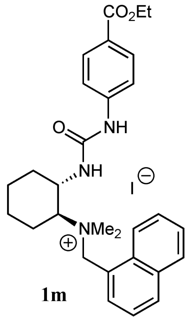

1m

Compound 1n. Obtained in 49% (2 steps, 0.2 mmol scale) as a yellowish oil. $[\alpha]_D^{23}$ (c = 0.3, DCM) = -44.0°; ^{1}H NMR (700 MHz, δ, $CDCl_3$, 298 K): 0.98 (t, *J* = 7.4 Hz, 3H), 1.34-1.52 (m, 4H), 1.67-1.88 (m, 4H), 1.90-2.06 (m, 2H), 2.18-2.25 (m, 1H), 2.57-2.67 (m, 1H), 2.85 (s, 3H), 3.14 (s, 3H), 4.31 (q, *J* = 7.4 Hz, 2H), 4.43-4.51 (m, 1H), 4.53-4.60 (m, 1H), 5.43 (d, *J* = 13.1 Hz, 1H), 5.63 (d, *J* = 13.1 Hz, 1H), 7.12-7.22 (m, 2H), 7.27-7.33 (m, 1H), 7.44 (d, *J* = 6.6 Hz, 1H), 7.66-7.75 (m, 2H), 7.75-7.84 (m, 3H), 8.00 (d, *J* = 8.4 Hz, 2H), 8.08 (d,

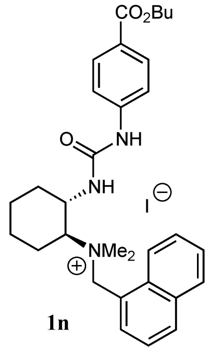

1n

J = 8.4 Hz, 1H), 9.13 (s, 1H) ppm; ^{13}C NMR (175 MHz, δ, CDCl$_3$, 298 K): 13.9, 19.4, 24.6, 25.1, 27.7, 31.1, 36.1, 47.6, 50.7, 51.1, 64.2, 64.7, 77.2, 117.7, 123.0, 123.3, 124.4, 124.7, 126.6, 128.1, 129.2, 130.9, 132.0, 133.0, 133.8, 134.0, 143.8, 155.1, 166.7 ppm; IR (film): $\tilde{\nu}$ = 3246, 3198, 3103, 3044, 2934, 2866, 1686, 1595, 1533, 1508, 1410, 1321, 1275, 1256, 1204, 1171, 1101, 912, 856, 806, 770, 727 cm^{-1}; HRMS (ESI): *m/z* calcd for $C_{31}H_{40}N_3O_3^+$: 502.3070 [M$^+$]; found: 502.3079.

Compound 1o. Obtained in 57% (2 steps, 0.2 mmol scale) as a yellowish oil. $[\alpha]_D^{23}$ (c = 0.75, DCM) = -49.1°; ^{1}H NMR (300 MHz, δ, CDCl$_3$, 298 K): 1.25-1.48 (m, 2H), 1.62-1.90 (m, 2H), 1.91-2.08 (m, 2H), 2.15-2.28 (m, 1H), 2.58-2.70 (m, 1H), 2.90 (s, 3H), 3.17 (s, 3H), 3.94 (s, 6H), 4.45-4.70 (m, 2H), 5.42 (d, 1H, J = 13.1 Hz), 5.70 (d, 1H, J = 13.1 Hz), 7.15 (t, 2H, J = 7.6 Hz), 7.24 (d, 1H, J = 7.8 Hz), 7.45 (d, 1H, J = 7.1 Hz), 7.66 (t, 2H, J = 8.6 Hz), 7.97 (d, 1H, J = 9.7 Hz), 8.08 (d, 1H, J = 8.6 Hz), 8.42 (t, 1H, J = 1.4 Hz), 8.56 (d, 2H, J = 1.4 Hz), 9.05 (s, 1H) ppm; ^{13}C NMR (75 MHz, δ, CDCl$_3$, 298 K): 24.5, 25.0, 27.4, 36.1, 48.0, 50.6, 51.4, 52.4, 63.5, 77.3, 122.9, 123.2, 123.8, 124.6, 125.0, 126.4, 127.8, 129.0, 131.2, 131.7, 132.8, 133.6, 133.9, 139.9, 155.2, 166.3 ppm; IR (film): $\tilde{\nu}$ = 3244, 3028, 2943, 2866, 1717, 1684, 1558, 1541, 1508, 1437, 1346, 1317, 1242, 1123, 1047, 997, 876, 808, 783, 754 cm^{-1}; HRMS (ESI): *m/z* calcd for $C_{30}H_{36}N_3O_5^+$: 518.2655 [M$^+$]; found: 518.2662.

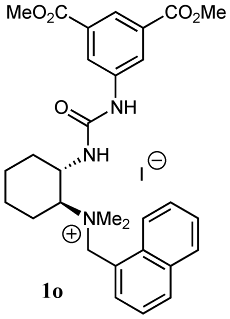

1o

Syntheses of Cyclohexanediamine-Based Catalysts 1p – 1r:

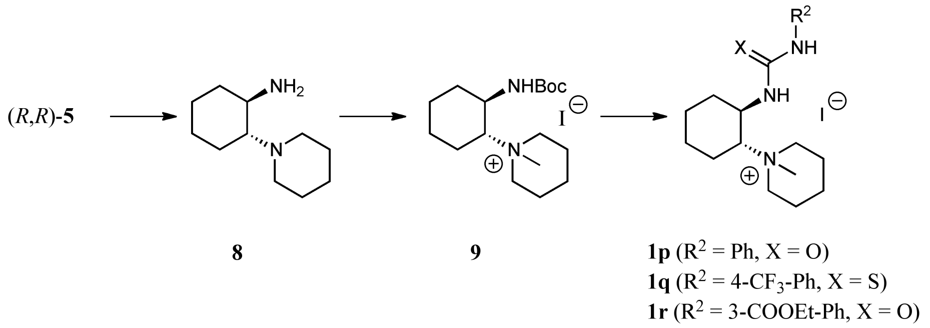

Synthesis of 9: *Step 1:* A mixture of **8** (prepared according to literature[4]) (1.817 g,

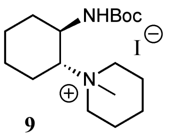

10.0 mmol) and di-*tert*-butyl dicarbonate (2.61 g, 12.0 mmol, 1.2 eq) in 50 ml DCM was stirred at r.t. for 20 h and the solvent was removed under reduced pressure. The product was purified by column chromatography (silica gel, DCM:MeOH, 40:1 → 10:1) to give the Boc-protected *tert*-amine in 72% yield (2.04 g, 7.2 mmol). *Step 2:* A mixture of *tert*-amine (378 mg, 1.3 mmol), K_2CO_3 185 mg (1.3 mmol, 1 eq) and 4 ml methyl iodide was stirred at 40 °C in a Schlenk flask for 3 d. Excess methyl iodide was removed under reduced pressure and the product was purified by column chromatography (silica gel, DCM:MeOH, 60:1 → 10:1) to give **9** in 45% (255 mg, 0.6 mmol). $[\alpha]_D^{23}$ (c = 1.3, DCM) = -2.4°; ^{1}H NMR (700 MHz, δ, $CDCl_3$, 298 K): 1.24-1.33 (m, 1H), 1.35-1.48 (m, 1H), 1.41 (s, 9H), 1.58-1.66 (m, 1H), 1.69-1.79 (m, 3H), 1.81-1.94 (m, 5H), 2.06-2.13 (m, 1H), 2.24-2.34 (m, 2H), 3.18 (s, 3Hg), 3.52-3.57 (m, 1H), 3.64-3.69 (m, 1Hh), 3.72-3.76 (m, 1H), 3.94-4.00 (m, 1H), 4.07-4.12 (m, 1H), 4.58-4.63 (m, 1H), 6.01 (d, 1H, J = 8.6 Hz) ppm; ^{13}C NMR (176 MHz, δ, $CDCl_3$, 298 K): 20.8, 20.9 (2x), 24.5, 24.7, 26.0, 28.5, 35.0, 45.2, 51.2, 61.4, 62.3, 71.7, 80.8, 155.5 ppm; IR (film): $\tilde{\nu}$ = 3237, 2968, 2934, 2862, 1692, 1508, 1450, 1391, 1366, 1321, 1275, 1242, 1155, 1109, 1043, 1020, 999, 957, 930, 885, 866, 856, 816, 783, 731 cm^{-1}; HRMS (ESI) *m/z* calcd for $C_{17}H_{33}N_2O_2^+$: 297.2537 [M^+], found: 297.2531.

General Syntheses of Catalysts 1p – 1r: *Step 1:* A solution of the quaternary ammonium salt **9** and trifluoroacetic acid (10 eq.) in DCM (10 mL / mmol) was stirred at r.t. for 2 h. After evaporation to dryness, the crude amine was directly subjected to the final coupling step. *Step 2:* A mixture of the amine, R^2NCX (1.5 eq.), and K_2CO_3 (3 eq.) in DCM (10 mL / mmol) was stirred at r.t. for 8-18 h. After filtration and evaporation to dryness, the crude product was purified by column chromatography (DCM:MeOH, 40:1 → 10:1) to obtain catalysts **1** in the reported yields.

4) Y. Zhu, J. Malerich, V. Rawal, *Angew. Chem. Int. Ed.* **2010**, *49*, 153-156.

Compound 1p. Obtained in 64% (0.2 mmol scale, 2 steps) as a colourless oil. $[\alpha]_D^{22}$ (c = 0.3, DCM) = +2.9°; ^{1}H NMR (300 MHz, δ, $CDCl_3$, 298 K): 1.22-1.58 (m, 3H), 1.60-1.99 (m, 8H), 2.01-2.11 (m, 1H), 2.17-2.33 (m, 2H), 3.14 (s, 3H), 3.16-3.27 (m, 1H), 3.54-3.71 (m, 2H), 3.89 (td, 1H, J_1 = 10.8 Hz, J_2 = 2.9 Hz), 4.14-4.29 (m, 1H), 4.23-4.33 (m, 1H), 6.97 (t, 1H, J = 7.4 Hz), 7.23 (dd, 2H, J_1 = 7.6 Hz, J_2 = 7.4 Hz), 7.29 (d, 1H, J = 10.0 Hz), 7.53 (d, 2H, J = 7.6 Hz), 8.49 (s, 1H) ppm; ^{13}C NMR (75 MHz, δ, $CDCl_3$, 298 K): 20.8, 21.0, 21.2, 24.5, 25.2, 26.0, 35.9, 46.1, 50.2, 62.0, 63.9, 71.4, 119.0, 122.7, 128.9, 139.4, 155.2 ppm; IR (film): $\bar{\nu}$ = 3254, 3190, 3034, 2938, 2864, 1686, 1597, 1545, 1499, 1441, 1321, 1265, 1221, 1207 cm^{-1}; HRMS (ESI): *m/z* calcd for $C_{19}H_{30}N_3O^+$: 316.2383 [M$^+$]; found: 316.2392.

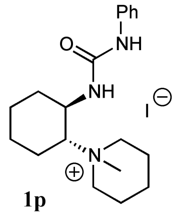

1p

Compound 1q. Obtained in 42% (0.1 mmol scale, 2 steps) as a colourless oil. $[\alpha]_D^{22}$ (c = 0.4, DCM) = +41.8°; ^{1}H NMR (300 MHz, δ, $CDCl_3$, 298 K): 1.23-1.60 (m, 3H), 1.66-2.02 (m, 8H), 2.10-2.22 (m, 1H), 2.23-2.36 (m, 2H), 3.21 (s, 3H), 3.26-3.36 (m, 1H), 3.48-3.59 (m, 1H), 3.59-3.70 (m, 1H), 4.04-4.14 (m, 1H), 4.11-4.21 (m, 1H), 5.12 (qd, 1H, J_1 = 10.1 Hz, J_2 = 4.3 Hz), 7.53 (d, 2H, J = 8.5 Hz), 7.93 (d, 2H, J = 8.5 Hz), 9.04 (d, 1H, J = 10.1 Hz), 9.89 (s, 1H) ppm; ^{13}C NMR (75 MHz, δ, $CDCl_3$, 298 K): 20.8, 21.0, 21.3, 24.0, 24.9, 25.7, 34.7, 46.2, 53.6, 61.9, 63.7, 72.8, 122.7, 124.3, 125.7, 126.5, 142.3, 180.0 ppm; IR (film): $\bar{\nu}$ = 2940, 2864, 1578, 1522, 1451, 1321, 1252, 1163, 1107, 1065, 1016, 843 cm^{-1}; HRMS (ESI): *m/z* calcd for $C_{20}H_{29}F_3N_3S^+$: 400.2030 [M$^+$]; found: 400.2032.

1q

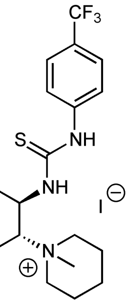

Compound 1r. Obtained in 46% (0.2 mmol scale, 2 steps) as a colourless oil. $[\alpha]_D^{22}$ (c = 1.2, DCM) = +1.1°; ^{1}H NMR (300 MHz, δ, $CDCl_3$, 298 K): 1.24-1.60 (m, 3H), 1.38 (t, 3H, J = 6.8 Hz), 1.69-2.05 (m, 8H), 2.07-2.15 (m, 1H), 2.23-2.40 (m, 2H), 3.20 (s, 3H), 3.218-3.32 (m, 1H), 3.56-3.66 (m, 1H), 3.67-3.78 (m, 1H), 3.90-4.01 (m, 1H), 4.22-4.40 (m, 2H), 4.35 (q, 2H, J = 6.8 Hz), 7.32 (t, 1H, J = 7.7 Hz), 7.48 (d, 1H, J = 10.0 Hz), 7.62-7.71 (m, 2H), 8.30 (s, 1H), 8.64 (s, 1H) ppm; ^{13}C NMR (75 MHz, δ, $CDCl_3$, 298 K): 14.4, 20.6, 20.9, 21.1, 24.3, 25.0, 25.8, 30.9, 35.7, 46.0, 50.0, 60.9, 62.0, 63.9, 71.2, 119.5, 123.3, 123.7, 128.7, 131.0, 139.4, 154.9, 166.7 ppm; IR (film): $\bar{\nu}$ = 3254, 3192, 3088, 3035, 2937, 2864, 1684, 1593, 1543, 1437, 1364, 1287, 1204, 1101, 1022, 858, 814, 754 cm^{-1}; HRMS (ESI): *m/z* calcd for $C_{22}H_{34}N_3O_3^+$: 388.2595 [M$^+$]; found: 388.2602.

1r

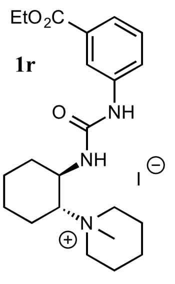

Synthesis of Cyclohexanediamine-Based Catalyst 1s:

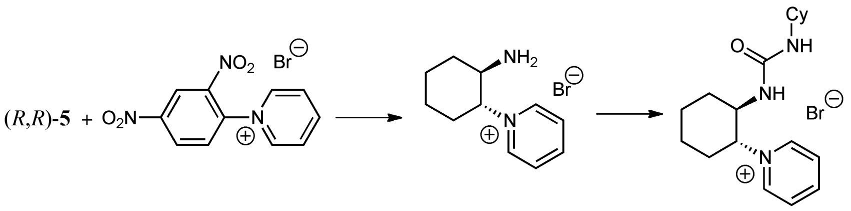

10 **11** **1s**

Compound 1s: *Step 1:* A mixture of **5** (170 mg, 1.49 mmol) and **10** (490 mg, 1 eq.) in DMSO (3 mL) was stirred at 90 °C for 1 d. DMSO was removed under reduced pressure and the crude **11** was directly submitted to the coupling step. *Step 2:* A mixture of the amine **11** (1.49 mmol), CyNCO (416 mg, 1.5 eq.), and K_2CO_3 (205 mg, 1 eq.) in DCM (3 mL) was stirred at r.t. for 18 h. After filtration and evaporation to dryness, the crude product was purified by column chromatography (DCM:MeOH, 40:1 → 10:1) to obtain **1s** in 41% (233 mg, 0.61 mmol). $[\alpha]_D^{23}$ (c = 0.27, DCM) = -96.3°; ^{1}H NMR (300 MHz, δ, CD_3OD, 298 K): 0.85-2.30 (m, 18H), 3.01-3.21 (m, 1H), 4.00-4.18 (m, 1H), 4.44-4.69 (m, 1H), 5.71 (d, 1H, J = 7.9 Hz), 8.01 (dd, 2H, J_1 = 7.8 Hz, J_2 = 6.4 Hz,), 8.58 (t, 1H, J = 7.8 Hz), 9.07 (d, 2H, J = 6.4 Hz), ppm; ^{13}C NMR (75 MHz, δ, CD_3OD, 298 K): 25.4, 25.7, 25.8, 25.9, 26.6, 31.2, 32.6, 33.8, 34.2, 34.5, 54.0, 78.1, 80.8, 128.9, 145.5, 147.2, 158.9 ppm; IR (film): $\tilde{\nu}$ = 3294, 3051, 2928, 2855, 1655, 1630, 1571, 1487, 1450, 1321, 1256, 1231, 1165, 779 cm^{-1}; HRMS (ESI) *m/z* calcd for $C_{18}H_{28}N_3O^+$: 302.2227 [M$^+$], found: 302.2232.

Synthesis of Diphenylethylenediamine-Based Catalyst 2a:

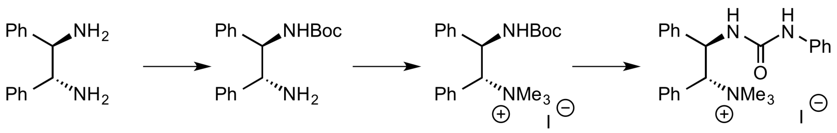

12 **32** **33** **2a**

Compound 2a: *Step 1:* A mixture of K_2CO_3 (620 mg, 4.48 mmol, 2 eq), mono-Boc-protected diamine **32** (prepared according to literature[5]) (630 mg, 2.01 mmol), and MeI (1mL, 16 mmol) in 50 ml AcN was stirred at 80 °C for 3 d. After filtration and evaporation to dryness the product was used without further purification for the deprotection and coupling. *Step 2:* A solution of **33** (350 mg, 0.73 mmol) and trifluoroacetic acid (0.56 mL, 10 eq.) in DCM (10

5) D. W. Lee, H.-J. Ha, W. K. Lee *Synth. Commun.* **2007**, *37*, 737-742.

mL) was stirred at r.t. for 3 h. After evaporation to dryness, the crude amine was directly subjected to the final coupling step. *Step 3:* A mixture of the amine (0.73 mmol), PhNCO (143 mg, 1.5 eq.), and K_2CO_3 (0.5 g, 3 eq.) in DCM (5 mL) was stirred at r.t. for 19 h. After filtration and evaporation to dryness, the crude product was purified by column chromatography (DCM:MeOH, 40:1 → 10:1) to obtain **2a** as an oily residue (0.14 g, 0.28 mmol, 38% over 3 steps). $[\alpha]_D^{23}$ (c = 0.95, DCM) = -127.4°; ^{1}H NMR (300 MHz, δ, $CDCl_3$, 298 K): 3.19 (s, 9H), 5.28 (d, 1H, J = 10.9 Hz), 5.87 (dd, 1H, J_1 = 10.9 Hz, J_2 = 9.5 Hz), 6.78-6.98 (m, 4H), 7.00-7.28 (m, 8H), 7.32-7.42 (m, 3H), 8.18 (d, 1H, J = 9.5 Hz), 8.97 (s, 1H) ppm; ^{13}C NMR (75 MHz, δ, $CDCl_3$, 298 K): 54.6, 54.8, 80.6, 119.0, 122.5, 126.9, 127.7, 127.8, 128.7, 128.8, 129.1, 129.5, 130.6, 131.2, 133.9, 139.2, 154.9 ppm; IR (film): $\tilde{\nu}$ = 3256, 3198, 3034, 2959, 2920, 2851, 1670, 1597, 1545, 1489, 1443, 1314, 1202, 1128, 949, 833, 800, 756, 731 cm^{-1}; HRMS (ESI) *m/z* calcd for $C_{24}H_{28}N_3O^+$: 347.2227 [M^+], found: 347.2233.

Synthesis of Anthracene-Based Catalyst 3a:

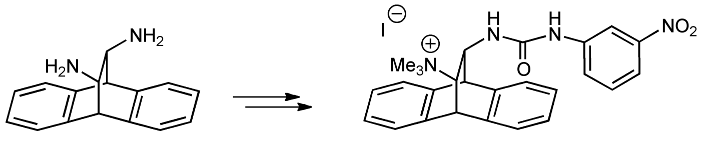

13 **3a**

Compound 3a: *Step 1:* Enantioenriched **13** (prepared according to literature[6]) (396 mg, 1.68 mmol) was dissolved in a mixture MeOH (3 mL) and H_2O (0.5 mL), cooled to 0 °C and HCl in MeOH (1.25 M, 1.8 mL) was added dropwise over 15 min. After further 45 min a solution of Boc_2O (376 mg, 2.16 mmol) in MeOH (2 mL) was added dropwise over 30 min and the mixture was stirred for 5 h at ambient temperature. After extraction with DCM and brine, drying of the organic phase over Na_2SO_4 and evaporation to dryness, the crude product was purified by column chromatography (DCM:MeOH = 10:1) to yield the mono-Boc-protected **13** (167 mg, 30%). ^{1}H NMR (300 MHz, δ, $CDCl_3$, 298 K): 1.41 (m, 2H), 2.80 (s, 1H), 3.47-3.45 (m, 1H), 4.13 (d, J = 2.6 Hz, 1H), 4.24 (s, 1H), 7.32-7.15 (m, 8H) ppm; ^{13}C NMR (75 MHz, δ, $CDCl_3$, 298 K): 28.5, 49.4, 52.3, 61.1, 61.2, 79.7, 124.5, 124.6, 125.7, 126.3, 126.5, 126.6, 126.7, 126.8, 138.9, 139.3, 140.8, 142.4, 155.8 ppm.

Step 2: K_2CO_3 (345 mg, 2.5 mmol, 5 eq) was added to a solution of mono-Boc-protected diamine (167mg, 0.5 mmol) in 5 ml AcN. After the addition of 310 µl (5 mmol, 10 eq) methyl

6) M. E. Fox, A. Gerlach, I. C. Lennon, G. Meek, C. Praquin, *Synthesis* **2005**, *19*, 3196-3198.

iodide the suspension was stirred for 1 d. After evaporation of excess methyl iodide and AcN, the residue was dissolved in DCM and filtered to give Boc-protected ammonium intermediate as an oily residue in quantitative yield. The product was used without further purification.
Step 3: A solution of the Boc-protected quaternary ammonium salt (178 mg, 0.35 mmol) and trifluoroacetic acid (270 µL, 10 eq.) in DCM (2 mL) was stirred at r.t. for 6 h. After evaporation to dryness, a mixture of this crude amine, 3-nitro phenylisocyanate (57mg, 0.35 mmol), and K_2CO_3 (240mg, 1.75 mmol) in AcN (2 mL) was stirred at r.t. for 18 h. After filtration and evaporation to dryness, the crude product was purified by column chromatography (DCM:MeOH, 40:1 → 10:1) to obtain catalyst **3a** as an oily residue (130mg, 66%). $[\alpha]_D^{23}$ (c = 3.2, MeOH) = +21.4°; ^{1}H NMR (300 MHz, δ, CD_3OD, 298 K): 3.08 (s, 9H), 3.68-3.77 (m, 1H), 4.43-52 (m, 1H), 4.60 (s, 1H), 5.17 (s, 1H), 7.28-7.59 (m, 8H), 7.61-7.64 (m, 2H), 7.82-7.88 (m, 1H), 8.52 (s, 1H) ppm; ^{13}C NMR (75 MHz, δ, CD_3OD, 298 K): 45.6, 51.7 (2x), 53.4, 81.9, 114.0, 118.0, 125.2, 125.9, 126.4, 127.0, 127.5, 128.6, 128.8, 128.9 128.9, 130.9, 138.8, 140.7, 141.3, 142.0, 150.1, 156.0 ppm; IR (film): $\tilde{\nu}$ = 3018, 2970, 2934, 1676, 1524, 1458, 1420, 1348, 1231, 1155, 951, 932, 880, 764 cm^{-1}; HRMS (ESI) *m/z* calcd for $C_{26}H_{27}N_4O_3^+$: 443.2078 [M$^+$], found: 443.2083.

Synthesis of Tartaric Acid-Based Catalysts 4:

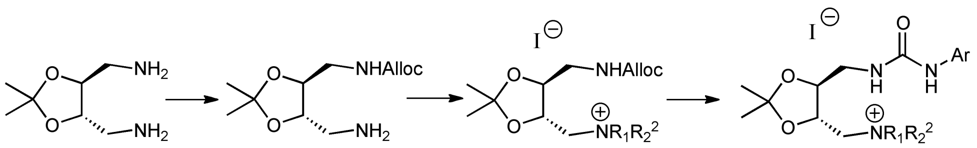

14 **15** **16**

4a ($R^1 = R^2$ = Me, Ar = Ph)
4b (R^1 = Bn, R^2 = Me, Ar = 3-NO_2-Ph)
4c (R^1 = Me, R^2 = Bn, Ar = 3-NO_2-Ph)

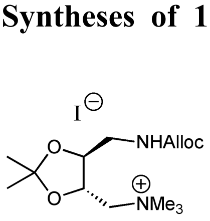

16a

Syntheses of 16a: *Step 1:* A solution of **14** (1.43 g, 8.9 mmol) (prepared according to literature[7]) in DCM (38 mL) was cooled to 0 °C. A solution of allylchloroformate (385 µL, 0.4 eq.) in DCM (10 mL) was added dropwise over 2 h. The mixture was stirred for further 16 h on an ice bath. After extraction with EtOAc/Na_2CO_3 (sat.) the organic layer was washed with brine, dried over Na_2SO_4 and evaporated to dryness to give a 2:1 mixture of **15** and the di-Alloc-protected amine which was used directly for the next step. *Step 2:* A mixture of crude

7) a) T. Shibuguchi, Y. Fukuta, Y. Akachi, A. Sekine, T. Ohshima, M. Shibasaki, *Tetrahedron Lett.* **2002**, *43*, 9539-9543; b) T. Ohshima, V. Gnanadesikan, T. Shibuguchi, Y. Fukuta, T. Nemoto, M. Shibasaki, *J. Am. Chem. Soc.* **2003**, *125*, 11206-11207; c) T. Ohshima, T. Shibuguchi, Y. Fukuta, M. Shibasaki, *Tetrahedron* **2004**, *60*, 7743-7754.

15, K_2CO_3 (744 mg, 5.4 mmol), and 1.2 ml methyl iodide in AcN (12 mL) was stirred at reflux for 2 d. Excess methyl iodide was removed under reduced pressure and the product was purified by column chromatography (silica gel, DCM:MeOH, 40:1 → 10:1) to obtain compound **16a** as an oily residue (840 mg, 23% over 2 steps). $[\alpha]_D^{23}$ (c = 0.5, DCM) = +9.8°; ^{1}H NMR (300 MHz, δ, $CDCl_3$, 298 K): 1.28 (s, 3H), 1.31 (s, 3H), 3.39 (s, 9H), 3.39-3.46 (m, 2H), 3.60-3.73 (m, 1H), 3.77-3.86 (m, 1H), 3.95-4.04 (m, 1H), 4.15-4.26 (m, 1H), 4.42 (d, 2H, *J* = 5.2 Hz), 5.05-5.11 (m, 1H), 5.14-5.24 (m, 1H), 5.70-5.85 (m, 1H), 5.88 (t, 1H, *J* = 6.2 Hz) ppm; ^{13}C NMR (75 MHz, δ, $CDCl_3$, 298 K): 27.0, 27.1, 40.8, 55.0, 65.6, 67.7, 72.3, 78.0, 110.9, 117.6, 132.8, 156.9 ppm; IR (film): $\tilde{\nu}$ = 3434, 3283, 2986, 2936, 1707, 1526, 1476, 1375, 1248, 1167, 1094, 991, 920, 845 cm^{-1}; HRMS (ESI) *m/z* calcd for $C_{14}H_{27}N_2O_4^+$: 287.1965 [M^+], found: 287.1972.

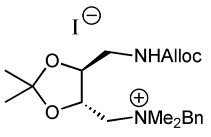

16b

Syntheses of 16b: *Step 1:* A solution of **14** (300 mg, 1.87 mmol) (prepared according to literature[7]) in DCM (8 mL) was cooled to 0 °C. A solution of allylchloroformate (80 µL, 0.4 eq.) in DCM (2 mL) was added dropwise over 2 h. The mixture was stirred for further 16 h on an ice bath. After extraction with EtOAc/Na_2CO_3 (sat.) the organic layer was washed with brine, dried over Na_2SO_4 and evaporated to dryness to give a 2:1 mixture of **15** and the di-Alloc-protected amine which was used directly for the next step. *Step 2:* Benzaldehyde (122 µL,1.2 mmol) was added to a solution of crude **15** in THF:MeOH = 1:1 (5 mL) and the solution was stirred at r.t. for 2 h. After the addition of $NaBH_4$ (68 mg) stirring was continued for another 16 h at r.t.. The reaction was quenched by addition of H_2O and extracted with H_2O/EtOAc. The organic phase was washed with brine, dried over Na_2SO_4, and evaporated to dryness to obtain the crude product which was directly used without any purification. *Step 3:* A mixture of the crude *sec*-amine and K_2CO_3 (138 mg, 1 mmol) in 2 ml methyl iodide was stirred at reflux for 3 d. Excess methyl iodide was removed under reduced pressure and the product was purified by column chromatography (silica gel, DCM:MeOH, 40:1 → 10:1) to obtain compound **16b** as an oily residue (101 mg, 11% over 3 steps). $[\alpha]_D^{23}$ (c = 0.85, DCM) = +2.0°; ^{1}H NMR (300 MHz, δ, $CDCl_3$, 298 K): 1.40 (s, 3H), 1.42 (s, 3H), 3.36 (s, 6H), 3.40-3.52 (m, 1H), 3.55-3.68 (m, 2H), 3.81-3.92 (m, 1H), 4.20 (d, 1H, *J* = 13.5 Hz), 4.39 (t, 1H, *J* = 9.0 Hz), 4.49 (d, 2H, *J* = 5.0 Hz), 4.93 (d, 1H, *J* = 12.5 Hz), 5.08 (d, 1H, *J* = 12.5 Hz), 5.15 (dd, 1H, J_1 = 10.1 Hz, J_2 = 1.2 Hz), 5.26 (dd, 1H, J_1 = 17.5 Hz, J_2 = 1.2 Hz), 5.78-5.92 (m, 1H), 6.00 (t, 1H, *J* = 6.4 Hz), 7.40-7.50 (m, 3H), 7.62-7.70 (m, 2H) ppm; ^{13}C NMR (75 MHz, δ, $CDCl_3$, 298 K): 27.1, 40.8, 50.7, 51.9, 64.7, 65.7, 69.1, 72.7, 78.3, 111.0, 117.6, 126.9,

129.3, 130.9, 132.8, 133.4, 157.0 ppm; IR (film): $\bar{\nu}$ = 3428, 2986, 2938, 1707, 1649, 1528, 1477, 1456, 1385, 1254, 1167, 1094, 993, 922 cm^{-1}; HRMS (ESI) *m/z* calcd for $C_{20}H_{31}N_2O_4^+$: 363.2278 [M^+], found: 363.2288.

Syntheses of 16c: *Step 1:* A solution of **14** (210 mg, 1.3 mmol) (prepared according to literature[7]) in DCM (5 mL) was cooled to 0 °C. A solution of allylchloroformate (55 µL, 0.4 eq.) in DCM (1 mL) was added dropwise over 2 h. The mixture was stirred for further 16 h on an ice bath. After extraction with EtOAc/Na_2CO_3 (sat.) the organic layer was washed with brine, dried over Na_2SO_4 and evaporated to dryness to give a 2:1 mixture of **15** and the di-Alloc-protected amine which was used directly for the next step. *Step 2:* Benzylbromide (105 µL, 0.88 mmol) was added to a mixture of crude **15** and K_2CO_3 (63 mg, 0.45 mmol) in AcN (3 mL) and the solution was stirred at reflux for 24 h. After filtration and evaporation to dryness MeI (2 mL) and K_2CO_3 (63 mg, 0.45 mmol) were added and the mixture was stirred at reflux for 2 d. Excess methyl iodide was removed under reduced pressure and the product was purified by column chromatography (silica gel, DCM:MeOH, 40:1 → 10:1) to obtain compound **16c** as an oily residue (146 mg, 22% overall). $[\alpha]_D^{23}$ (c = 0.45, DCM) = -23.3°; ^{1}H NMR (300 MHz, δ, $CDCl_3$, 298 K): 1.41 (s, 6H), 3.09 (s, 3H), 3.32-3.60 (m, 3H), 3.81-3.90 (m, 1H), 4.22 (d, 1H, *J* = 13.4 Hz), 4.35-4.41 (m, 2H), 4.42-4.56 (m, 1H), 4.60 (d, 1H, *J* = 12.3 Hz), 4.75 (d, 1H, *J* = 12.4 Hz), 5.05-5.14 (m, 2H), 5.23 (d, 1H, *J* = 17.4 Hz), 5.42 (d, 1H, *J* = 12.4 Hz), 5.72-5.88 (m, 1H), 6.11 (t, 1H, *J* = 5.9 Hz), 7.35-7.50 (m, 6H), 7.55-7.71 (m, 4H) ppm; ^{13}C NMR (75 MHz, δ, $CDCl_3$, 298 K): 27.1, 27.2, 41.0, 47.7, 61.3, 65.5, 65.6, 66.5, 72.8, 78.4, 111.0, 117.5, 126.7, 126.9, 129.3, 129.4, 130.9, 132.7, 133.4, 133.5, 156.9 ppm; IR (film): $\bar{\nu}$ = 3275, 3032, 2985, 2935, 1707, 1522, 1508, 1456, 1373, 1236, 1217, 1159, 1086, 991, 926, 785, 754, 725 cm^{-1}; HRMS (ESI) *m/z* calcd for $C_{26}H_{35}N_2O_4^+$: 439.2591 [M^+], found: 439.2594.

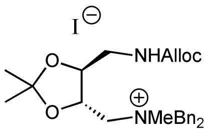

16c

General Syntheses of Catalysts 4a – 4c: *Step 1:* A solution of the quaternary ammonium salt **16**, $Pd(PPh_3)_4$ (5 mol%), $NaBH_4$ (3 eq.) in DCM:MeOH = 1:1 was stirred at r.t. for 2 h. After filtration and evaporation to dryness, the crude amine was directly subjected to the final coupling step. *Step 2:* A mixture of the amine, R^2NCX (1.5 eq.), and K_2CO_3 (3 eq.) in DCM (20 mL / mmol amine) was stirred at r.t. for 18 h. After filtration and evaporation to dryness, the crude product was purified by column chromatography (DCM:MeOH, 40:1 → 10:1) to obtain catalysts **4** in the reported yields.

Compound 4a. Obtained in 51% (2 steps, 1 mmol scale) as a slightly yellow oil. $[\alpha]_D^{23}$ (c = 0.8, DCM) = +15.4°; ^{1}H NMR (300 MHz, δ, $CDCl_3$, 298 K): 1.35 (s, 3H), 1.38 (s, 3H), 3.28 (s, 9H), 3.32-3.48 (m, 2H), 3.68-3.79 (m, 1H), 3.81-3.93 (m, 1H), 4.25-4.35 (m, 1H), 4.45 (d, 1H, *J* = 13.9 Hz), 6.43 (t, 1H, *J* = 7.5 Hz), 6.90 (t, 1H, *J* = 7.4 Hz), 7.17 (t, 2H, *J* = 8.1 Hz), 7.46 (d, 2H, *J* = 7.8 Hz), 8.30 (s, 1H) ppm; ^{13}C NMR (75 MHz, δ, $CDCl_3$, 298 K): 27.0, 27.1, 41.1, 55.1, 68.7, 74.4, 79.9, 111.5, 118.9, 122.5, 128.8, 139.4, 156.0 ppm; IR (film): $\bar{\nu}$ = 3414, 2988, 2928, 1674, 1599, 1551, 1499, 1441, 1385, 1314, 1238, 1170, 1105, 1084, 920 cm^{-1}; HRMS (ESI): *m/z* calcd for $C_{17}H_{28}N_3O_3^+$: 322.2131 [M$^+$]; found: 322.2132.

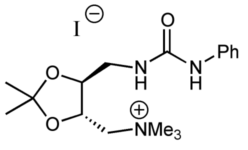

4a

Compound 4b. Obtained in 69% (2 steps, 0.3 mmol scale) as a yellowish oil. $[\alpha]_D^{23}$ (c = 1.35, DCM) = +10.7°; (300 MHz, δ, $CDCl_3$, 298 K): 1.41 (s, 3H), 1.44 (s, 3H), 3.20 (s, 3H), 3.28 (s, 3H), 3.28-3.48 (m, 2H), 3.88-4.02 (m, 2H), 4.45-4.53 (m, 1H), 4.62-4.70 (m, 1H), 4.71 (d, 1H, *J* = 12.3 Hz), 4.94 (d, 1H, *J* = 12.3 Hz), 6.78 (t, 1H, *J* = 6.4 Hz), 7.28-7.43 (m, 3H), 7.44-7.54 (m, 3H), 7.70-7.78 (m, 2H), 8.66 (t, 1H, *J* = 1.9 Hz), 8.93 (s, 1H) ppm; ^{13}C NMR (75 MHz, δ, $CDCl_3$, 298 K): 27.0, 27.1, 41.3, 50.6, 51.5, 67.1, 69.6, 74.6, 77.5, 111.5, 112.8, 116.8, 124.0, 126.0, 129.3, 129.5, 131.4, 133.0, 140.8, 148.6, 155.5 ppm; IR (film): $\bar{\nu}$ = 3264, 3065, 2985, 2935, 2874, 1690, 1596, 1526, 1481, 1350, 1223, 1080, 999, 783 cm^{-1}; HRMS (ESI): *m/z* calcd for $C_{23}H_{31}N_4O_5^+$: 443.2289 [M$^+$]; found: 443.2297.

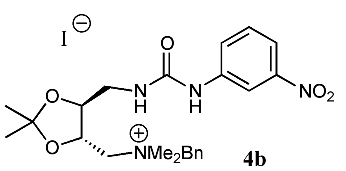

4b

Compound 4c. Obtained in 40% (2 steps, 0.5 mmol scale) as a yellowish oil. $[\alpha]_D^{23}$ (c = 0.9, DCM) = -18.8°; ^{1}H NMR (300 MHz, δ, $CDCl_3$, 298 K): 1.35 (s, 3H), 1.43 (s, 3H), 2.93 (s, 3H), 2.97-3.13 (m, 1H), 3.20-3.35 (m, 1H), 3.82-3.97 (m, 2H), 4.03 (d, 1H, *J* = 12.4 Hz), 4.48-4.68 (m, 2H), 4.61 (d, 1H, *J* = 12.4 Hz), 5.08 (d, 1H, *J* = 12.4 Hz), 5.53 (d, 1H, *J* = 12.4 Hz), 6.91 (t, 1H, *J* = 6.0 Hz), 7.20-7.75 (m, 14H), 8.63 (t, 1H, *J* = 2.1 Hz), 8.94 (s, 1H) ppm; ^{13}C NMR (75 MHz, δ, $CDCl_3$, 298 K): 26.9, 27.1, 41.2, 47.7, 63.6, 65.3, 66.4, 74.3, 77.7, 111.4, 112.8, 116.7, 123.9, 126.2, 128.5, 128.7, 129.2, 129.5, 129.6, 131.3, 132.1, 132.2, 133.2, 133.3, 140.8, 148.6, 155.4 ppm; IR (film): $\bar{\nu}$ = 3281, 3065, 2986, 2934, 1697, 1595, 1541, 1526, 1350, 1223, 1166, 1093, 785, 754 cm^{-1}; HRMS (ESI): *m/z* calcd for $C_{29}H_{35}N_4O_5^+$: 519.2602 [M$^+$]; found: 519.2610.

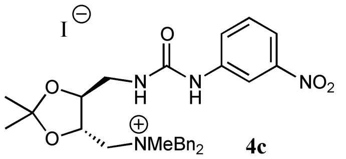

4c

3. Asymmetric α-Fluorination:

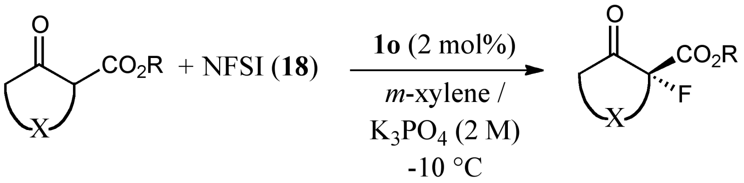

General procedure for the α-fluorination of β-ketoesters: Reactions were usually carried out using 0.1 – 0.5 mmol of the ketoester. Aqueous K_3PO_4 (2M, 2 eq.) was added to a mixture of ketoester and catalyst **1o** (2 mol%) in *m*-xylene (20 mL / mmol ketoester) and the mixture was cooled to -10 °C. NFSI was added portion-wise over 2 h and the mixture was heavily stirred for another 10 h at -10 °C (Ar-atmosphere). The reaction was quenched by addition of $NH_4Cl_{(sat)}$ and the mixture was extracted with CH_2Cl_2. After drying over Na_2SO_4, and evaporation to dryness, the product was purified by silica gel column chromatography (heptanes:EtOAc = 20:1) to give the products in the reported yields.

***(R)*-19a.** Obtained in 95% yield with *e.r.* = 92 : 8 upon reacting **17a** (112 mg, 0.48 mmol) with NFSI (**18**). Analytical data are in accordance with those reported in literature.[8] $[\alpha]_D^{23}$ (c = 0.65, $CHCl_3$) = +3.2°; ^{1}H NMR (700 MHz, δ, $CDCl_3$, 298 K): 1.41 (s, 9H), 3.38 (dd, *J* = 22.8, 17.6 Hz, 1H), 3.71 (dd, *J* = 17.6, 10.9 Hz, 1H), 7.44 (t, *J* = 6.8 Hz, 1H), 7.48 (d, *J* = 7.4 Hz, 1H), 7.67 (t, *J* = 7.6 Hz, 1H), 7.81 (d, *J* = 7.7 Hz, 1H) ppm; ^{13}C NMR (175 MHz, δ, $CDCl_3$, 298 K): 27.9, 38.4 (d, *J* = 24.1 Hz), 84.2, 94.5 (d, *J* = 202.4 Hz), 125.5, 126.6, 128.6, 133.7, 136.6, 151.1 (d, *J* = 3.5 Hz), 166.4 (d, *J* = 27.9 Hz), 195.9 (d, *J* = 17.9 Hz) ppm; ^{19}F NMR (282 MHz, δ, $CDCl_3$, 298 K): -164.0 (dd, *J* = 22.8, 10.9 Hz) ppm; IR (film): $\tilde{\nu}$ = 3003, 2981, 2936, 1753, 1717, 1607, 1466, 1370, 1296, 1209, 1152, 1074, 924, 835, 746, 723 cm^{-1}; HRMS (ESI): *m/z* calcd for $C_{14}H_{15}FO_3$: 268.13435 $[M+NH_4]^+$; found: 268.13488. The enantioselectivity was determined by HPLC (Chiralpak AD-H, eluent: hexane:i-PrOH = 200:1, 0.75 mL/min, 10°C, retention times: (*S*)-enantiomer 25.0 min, (*R*)-enantiomer 32.2 min).

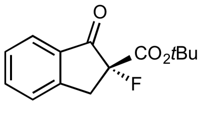

19a

8) a) D. Y. Kim, E. J. Park, *Org. Lett.* **2002**, *4*, 545-547; b) X. Wang, Q. Lan, S. Shirakawa, K. Maruoka, *Chem. Commun.* **2010**, *46*, 321-323; c) E.-M. Tanzer, W. B. Schweizer, M.-O. Ebert, R. Gilmour, *Chem. Eur. J.* **2012**, *18*, 2006-2013; d) J. Luo, W. Wu, L.-W. Xu, Y. Meng, Y. Lu, *Tetrahedron Lett.* **2013**, *54*, 2623-2626.

19b. Obtained in 92% yield with *e.r.* = 85 : 15 upon reacting **17b** (106 mg, 0.56 mmol) with NFSI (**18**). The product can recrystallized from heptanes/EtOAc to obtain almost enantiopure material (*e.r.* = 99 : 1). Analytical data are in accordance with those reported in literature.[8] $[\alpha]_D^{23}$ (c = 0.2, CH_2Cl_2) = -27.0°; ^1H NMR (700 MHz, δ, $CDCl_3$, 298 K): 3.44 (dd, J = 23.3, 17.5 Hz, 1H), 3.80 (dd, J = 17.5, 11.7 Hz, 1H), 3.82 (s, 3H), 7.42-7.52 (m, 2H), 7.71 (t, J = 7.7 Hz, 1H), 7.85 (d, J = 7.7 Hz, 1H) ppm; ^{13}C NMR (175 MHz, δ, $CDCl_3$, 298 K): 38.5 (d, J = 23.0 Hz), 53.4, 94.7 (d, J = 201.9 Hz), 125.8, 126.7, 128.8, 133.3, 136.9, 150.9 (d, J = 3.8 Hz), 167.8 (d, J = 27.7 Hz), 195.2 (d, J = 18.2 Hz) ppm; ^{19}F NMR (282 MHz, δ, $CDCl_3$, 298 K): -164.5 (dd, J = 23.3, 11.7 Hz) ppm; IR (film): $\bar{\nu}$ = 2961, 2918, 2849, 1769, 1721, 1611, 1589, 1477, 1422, 1267, 1198, 1180, 1074, 1051, 924, 812, 752, 716 cm^{-1}; HRMS (ESI): *m/z* calcd for $C_{11}H_9FO_3$: 209.0608 $[M+H]^+$; found: 209.0613. The enantioselectivity was determined by HPLC (Chiralcel OD-H, eluent: hexane:i-PrOH = 95:5, 0.75 mL/min, 10°C, retention times: major 28.8 min, minor 36.5 min).

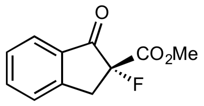

19b

19c. Obtained in 86% yield with *e.r.* = 85 : 15 upon reacting **17c** (105 mg, 0.4 mmol) with NFSI (**18**). Analytical data are in accordance with those reported in literature.[8] $[\alpha]_D^{23}$ (c = 1.07, CH_2Cl_2) = -8.1°; ^1H NMR (300 MHz, δ, $CDCl_3$, 298 K): 3.35 (dd, J = 22.6, 17.7 Hz, 1H), 3.69 (dd, J = 17.7, 11.8 Hz, 1H), 5.13 (d, J = 12.2 Hz, 1H), 5.20 (d, J = 12.2 Hz, 1H), 7.10-7.28 (m, 5H), 7.37-7.44 (m, 2H), 7.61 (t, J = 7.3 Hz, 1H), 7.75 (d, J = 7.3 Hz, 1H) ppm; ^{19}F NMR (282 MHz, δ, $CDCl_3$, 298 K): -164.5 (dd, J = 22.6, 11.8 Hz) ppm; IR (film): $\bar{\nu}$ = 3096, 3064, 3037, 1761, 1720, 1607, 1585, 1450, 1377, 1294, 1263, 1213, 1184, 1078, 926, 800, 739 cm^{-1}; HRMS (ESI): *m/z* calcd for $C_{17}H_{13}FO_3$: 302.11870 $[M+NH_4]^+$; found: 302.11951. The enantioselectivity was determined by HPLC (Chiralpak AD-H, eluent: hexane:i-PrOH = 90:10, 0.75 mL/min, 10°C, retention times: minor 22.7 min, major 24.4 min).

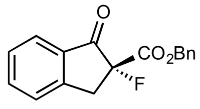

19c

19d. Obtained in 52% yield with *e.r.* = 89 : 11 upon reacting **17e** (21 mg, 0.07 mmol) with NFSI (**18**). The compound tends to hydrolyse and decarboxylate during column chromatography. $[\alpha]_D^{23}$ (c = 0.36, CH_2Cl_2) = +10.8°; ^1H NMR (300 MHz, δ, $CDCl_3$, 298 K): 1.79 (s, 3H), 1.80 (s, 3H), 3.45 (dd, J = 22.6, 17.5 Hz, 1H), 3.78 (dd, J = 17.5, 10.3 Hz, 1H), 7.19-7.35 (m, 5H), 7.48-7.55 (m, 2H), 7.72 (t, J = 7.6 Hz, 1H), 7.88 (d, J = 7.5 Hz, 1H) ppm; ^{13}C NMR (75 MHz, δ, $CDCl_3$, 298 K): 28.1, 28.3, 38.2 (d, J = 22.6 Hz), 85.2, 94.6 (d, J = 204.8 Hz), 124.1, 125.6, 126.5 (d, J = 1.3 Hz), 127.4, 128.4, 128.6, 129.5, 136.5, 144.5, 150.8 (d, J = 4.0 Hz), 165.6 (d, J = 26.5 Hz),

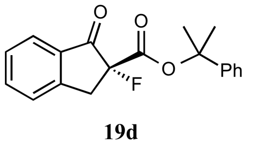

19d

195.7 (d, J = 17.6 Hz) ppm; ^{19}F NMR (282 MHz, δ, $CDCl_3$, 298 K): -164.6 (dd, J = 22.6, 10.3 Hz) ppm; IR (film): $\tilde{\nu}$ = 2986, 1763, 1724, 1607, 1449, 1300, 1273, 1196, 1138, 1072, 924, 789, 764 cm^{-1}; HRMS (ESI): *m/z* calcd for $C_{19}H_{17}FO_3$: 330.15000 $[M+NH_4]^+$; found: 330.15058. The enantioselectivity was determined by HPLC (Chiralpak AD-H, eluent: hexane:i-PrOH = 90:10, 0.75 mL/min, 10°C, retention times: major 14.2 min, minor 15.8 min).

19e. Obtained in 97% yield with *e.r.* = 93 : 7 upon reacting **17e** (99 mg, 0.32 mmol) with NFSI (**18**). Analytical data are in accordance with those reported in literature.[8] $[\alpha]_D^{23}$ (c = 0.92, CH_2Cl_2) = -0.8°; ^{1}H NMR (300 MHz, δ, $CDCl_3$, 298 K): 1.54 (s, 6H), 1.96 (s, 6H), 2.06 (s, 3H), 3.31 (dd, J = 22.8, 17.5 Hz, 1H), 3.65 (dd, J = 17.5, 10.1 Hz, 1H), 7.35-7.46 (m, 2H), 7.60 (t, J = 7.2 Hz, 1H), 7.75 (d, J = 7.2 Hz, 1H) ppm; ^{13}C NMR (75 MHz, δ, $CDCl_3$, 298 K): 30.6, 35.8, 38.2 (d, J = 24.0 Hz), 40.9, 84.0, 94.1 (d, J = 200.8 Hz) , 125.2, 126.4, 128.2, 133.5, 136.1, 150.8 (d, J = 4.0 Hz), 165.6 (d, J = 27.5 Hz), 195.6 (d, J =18.0 Hz) ppm; ^{19}F NMR (282 MHz, δ, $CDCl_3$, 298 K): -164.1 (dd, J = 22.8, 10.1 Hz) ppm; IR (film): $\tilde{\nu}$ = 2910, 2853, 1759, 1724, 1607, 1458, 1287, 1194, 1072, 1049, 922, 754 cm^{-1}; HRMS (ESI): *m/z* calcd for $C_{20}H_{21}FO_3$: 346.18285 $[M+NH_4]^+$; found: 346.18330. The enantioselectivity was determined by HPLC (Chiralcel OD-R, eluent: H_2O:AcN = 55:45, 0.8 mL/min, 10 °C, retention times: major 90.6 min, minor 97.0 min).

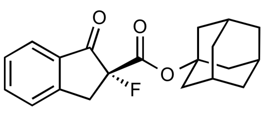

19e

23a. Obtained in 73% yield with *e.r.* = 88 : 12 upon reacting **22a** (21 mg, 0.07 mmol) with NFSI (**18**). Analytical data are in full accordance with those reported in literature.[9] $[\alpha]_D^{23}$ (c = 0.28, CH_2Cl_2) = -27.5°; ^{1}H NMR (300 MHz, δ, $CDCl_3$, 298 K): 1.46 (s, 9H), 3.40 (dd, J = 22.5, 17.4 Hz, 1H), 3.73 (dd, J = 17.4, 11.3 Hz, 1H), 7.58-7.66 (m, 1H), 7.68-7.74 (m, 2H) ppm; ^{13}C NMR (75 MHz, δ, $CDCl_3$, 298 K): 27.8, 37.9 (d, J = 24.8 Hz), 84.5, 94.3 (d, J = 201.6 Hz), 126.5, 129.8 (d, J = 1.1 Hz), 132.1, 132.2, 132.4 (d, J = 1.3 Hz), 152.3 (d, J = 3.8 Hz), 165.7 (d, J = 29.8 Hz), 194.7 (d, J =18.6 Hz) ppm; ^{19}F NMR (282 MHz, δ, $CDCl_3$, 298 K): -163.4 (dd, J = 22.5, 11.3 Hz) ppm; IR (film): $\tilde{\nu}$ = 3069, 2972, 2928, 1759, 1724, 1597, 1576, 1368, 1288, 1269, 1213, 1150, 1084, 1057, 932, 912, 845, 835, 799, 785 cm^{-1}; HRMS (ESI): *m/z* calcd for $C_{14}H_{14}BrFO_3$: 346.04486 $[M+NH_4]^+$; found: 346.04519. The enantioselectivity was determined by HPLC (Chiralcel OD-H, eluent: hexane:i-PrOH = 95:5, 0.75 mL/min, 10°C,

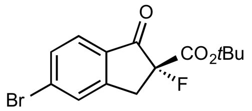

23a

9) Q-H. Deng, H. Wadepohl, L. H. Gade, *Chem. Eur. J.* **2011**, *17*, 14922-14928.

retention times: major 12.3 min, minor 14.3 min).

23b. Obtained in 85% yield with *e.r.* = 89 : 11 upon reacting **22b** (25 mg, 0.07 mmol) with NFSI (**18**). $[\alpha]_D^{23}$ (c = 0.51, CH_2Cl_2) = -38.8°; ^{1}H NMR (700 MHz, δ, $CDCl_3$, 298 K): 1.62 (s, 6H), 2.04 (s, 6H), 2.15 (s, 3H), 3.37 (dd, *J* = 22.5, 17.2 Hz, 1H), 3.70 (dd, *J* = 17.2, 10.7 Hz, 1H), 7.59 (d, *J* = 8.1 Hz, 1H), 7.67 (s, 1H), 7.68 (d, *J* = 8.1 Hz, 1H), ppm; ^{13}C NMR (175 MHz, δ, $CDCl_3$, 298 K): 31.0, 36.0, 38.1 (d, *J* = 24.3 Hz), 41.2, 84.6, 94.2 (d, *J* = 201.1 Hz), 126.6, 129.9, 132.1, 132.3, 132.6, 152.5 (d, *J* = 3.9 Hz), 165.5 (d, *J* = 28.4 Hz), 194.8 (d, *J* =19.0 Hz) ppm; ^{19}F NMR (282 MHz, δ, $CDCl_3$, 298 K): -163.6 (dd, *J* = 22.5, 10.7 Hz) ppm; IR (film): $\tilde{\nu}$ = 2913, 2849, 1757, 1726, 1595, 1415, 1265, 1196, 1078, 1051, 914, 833 cm^{-1}; HRMS (ESI): *m/z* calcd for $C_{20}H_{20}BrFO_3$: 424.09181 $[M+NH_4]^+$; found: 424.09321. The enantioselectivity was determined by HPLC (Chiralpak AD-H, eluent: hexane:i-PrOH = 90:10, 0.75 mL/min, 10°C, retention times: minor 16.3 min, major 22.8 min).

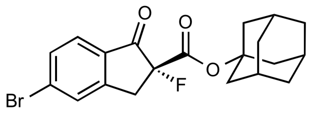

23b

25a. Obtained in 75% yield with *e.r.* = 91 : 9 upon reacting **24a** (25 mg, 0.07 mmol) with NFSI (**18**). Analytical data are in full accordance with those reported in literature.[8d] $[\alpha]_D^{23}$ (c = 0.3, CH_2Cl_2) = -1.2°; ^{1}H NMR (300 MHz, δ, $CDCl_3$, 298 K): 1.46 (s, 9H), 3.41 (dd, *J* = 22.5, 17.9 Hz, 1H), 3.74 (dd, *J* = 17.9, 10.7 Hz, 1H), 7.11-7.25 (m, 2H), 7.82-7.92 (m, 1H) ppm; ^{13}C NMR (75 MHz, δ, $CDCl_3$, 298 K): 27.8, 38.2 (d, *J* = 24.7 Hz), 84.4, 94.4 (d, *J* = 202.1 Hz), 113.3 (d, *J* = 25.9 Hz), 116.9 (d, *J* = 23.3 Hz), 128.0 (d, *J* = 11.0 Hz), 130.0, 153.9 (dd, *J* = 10.4, 4.1 Hz), 165.9 (d, *J* = 26.4 Hz), 167.8 (d, *J* = 257.7 Hz), 193.8 (d, *J* =17.6 Hz) ppm; ^{19}F NMR (282 MHz, δ, $CDCl_3$, 298 K): -98.8 (m), -163.2 (dd, *J* = 22.5, 10.7 Hz) ppm; IR (film): $\tilde{\nu}$ = 2982, 2938, 1763, 1734, 1616, 1595, 1483, 1371, 1259, 1156, 1078, 943, 839 cm^{-1}; HRMS (ESI): *m/z* calcd for $C_{14}H_{14}F_2O_3$: 286.12493 $[M+NH_4]^+$; found: 286.12564. The enantioselectivity was determined by HPLC (Chiralpak AD-H, eluent: hexane:i-PrOH = 95:5, 0.75 mL/min, 10°C, retention times: minor 16.3 min, major 13.2 min).

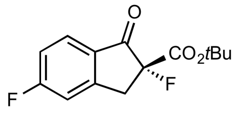

25a

25b. Obtained in 86% yield with *e.r.* = 92 : 8 upon reacting **24b** (25 mg, 0.07 mmol) with NFSI (**18**). $[\alpha]_D^{23}$ (c = 0.32, CH_2Cl_2) = -0.9°; ^{1}H NMR (700 MHz, δ, $CDCl_3$, 298 K): 1.62 (s, 6H), 2.05 (s, 6H), 2.15 (s, 3H), 3.38 (dd, *J* = 22.2, 17.8 Hz, 1H), 3.71 (dd, *J* = 17.8, 10.6 Hz, 1H), 7.10-7.20 (m, 2H), 7.80-7.88 (m, 1H) ppm; ^{13}C NMR (175 MHz, δ, $CDCl_3$, 298 K): 31.0, 36.0, 38.4 (d, *J* =

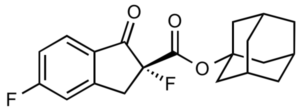

25b

24.4 Hz), 41.2, 84.6, 94.3 (d, J = 204.8 Hz), 113.5 (d, J = 22.8 Hz), 117.0 (d, J = 23.8 Hz), 128.0 (d, J = 10.4 Hz), 130.2, 154.0 (dd, J = 10.4, 4.0 Hz), 165.5 (d, J = 27.8 Hz), 168.0 (d, J = 259.7 Hz), 194.0 (d, J =18.3 Hz) ppm; ^{19}F NMR (282 MHz, δ, $CDCl_3$, 298 K): -98.8 (m), -163.3 (dd, J = 22.2, 10.6 Hz) ppm; IR (film): $\bar{\nu}$ = 2913, 2855, 1761, 1732, 1616, 1595, 1483, 1458, 1373, 1258, 1198, 1076, 1049, 943 cm^{-1}; HRMS (ESI): *m/z* calcd for $C_{20}H_{20}F_2O_3$: 364.17188 $[M+NH_4]^+$; found: 364.17256. The enantioselectivity was determined by HPLC (Chiralpak AD-H, eluent: hexane:i-PrOH = 90:10, 0.75 mL/min, 10°C, retention times: minor 16.3 min, major 21.2 min).

27a. Obtained in 79% yield with *e.r.* = 85 : 15 upon reacting **26a** (20 mg, 0.07 mmol) with NFSI (**18**). $[\alpha]_D^{23}$ (c = 0.37, CH_2Cl_2) = +9.2°; ^{1}H NMR (300 MHz, δ, $CDCl_3$, 298 K): 1.37 (s, 9H), 1.47 (s, 9H), 3.36 (dd, J = 23.0, 17.5 Hz, 1H), 3.71 (dd, J = 17.5, 11.3 Hz, 1H), 7.43 (d, J = 7.9 Hz, 1H), 7.77 (dd, J = 7.9, 1.9 Hz, 1H), 7.85 (d, J = 1.9 Hz, 1H) ppm; ^{13}C NMR (75 MHz, δ, $CDCl_3$, 298 K): 27.9, 31.2, 34.9, 38.0 (d, J = 23.7 Hz), 84.1, 94.7 (d, J = 200.1 Hz), 121.7, 126.0, 133.4, 134.4, 148.6 (d, J = 3.8 Hz), 152.1, 166.5 (d, J = 27.5 Hz), 196.1 (d, J =16.6 Hz) ppm; ^{19}F NMR (282 MHz, δ, $CDCl_3$, 298 K): -163.3 (dd, J = 23.0, 11.3 Hz) ppm; IR (film): $\bar{\nu}$ = 2965, 2938, 2911, 2870, 1761, 1719, 1616, 1495, 1369, 1287, 1256, 1213, 1194, 1155, 1074, 957, 839, 760 cm^{-1}; HRMS (ESI): *m/z* calcd for $C_{18}H_{23}FO_3$: 324.19695 $[M+NH_4]^+$; found: 324.19740. The enantioselectivity was determined by HPLC (Chiralpak AD-H, eluent: hexane:i-PrOH = 95:5, 0.75 mL/min, 10°C, retention times: major 7.4 min, minor 7.9 min).

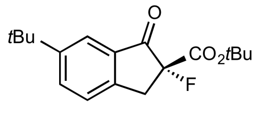

27a

27b. Obtained in 96% yield with *e.r.* = 86 : 14 upon reacting **26b** (27 mg, 0.07 mmol) with NFSI (**18**). $[\alpha]_D^{23}$ (c = 0.56, CH_2Cl_2) = +11.8°; ^{1}H NMR (700 MHz, δ, $CDCl_3$, 298 K): 1.34 (s, 9H), 1.62 (s, 6H), 2.07 (s, 6H), 2.15 (s, 3H), 3.33 (dd, J = 22.2, 17.0 Hz, 1H), 3.68 (dd, J = 17.0, 11.1 Hz, 1H), 7.40 (d, J = 7.5 Hz, 1H), 7.73 (d, J = 7.5 Hz, 1H), 7.83 (s, 1H) ppm; ^{13}C NMR (175 MHz, δ, $CDCl_3$, 298 K): 31.0, 31.3, 35.0, 36.0, 38.1 (d, J = 24.0 Hz), 41.2, 84.2, 94.8 (d, J = 200.9 Hz), 121.8, 126.1, 133.6, 134.5, 148.7 (d, J = 3.5 Hz), 152.2, 166.2 (d, J = 27.1 Hz), 196.3 (d, J =18.8 Hz) ppm; ^{19}F NMR (282 MHz, δ, $CDCl_3$, 298 K): -163.3 (dd, J = 22.2, 11.1 Hz) ppm; IR (film): $\bar{\nu}$ = 2957, 2913, 2855, 1763, 1719, 1541, 1286, 1192, 1074, 1049, 792 cm^{-1}; HRMS (ESI): *m/z* calcd for $C_{24}H_{30}FO_3$: 402.24390 $[M+NH_4]^+$; found: 402.24465. The enantioselectivity was determined by HPLC (Chiralpak AD-H, eluent: hexane:i-PrOH = 90:10, 0.75 mL/min, 10°C, retention times: minor 10.4 min, major 11.4 min).

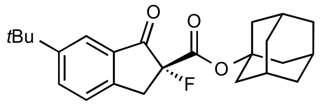

27b

29. Obtained in 73% yield with *e.r.* = 87 : 13 upon reacting **28** (97 mg, 0.4 mmol) with NFSI (**18**). Analytical data are in full accordance with those reported in literature.[8b] $[\alpha]_D^{23}$ (c = 1.03, CH_2Cl_2) = +6.4°; ^1H NMR (300 MHz, δ, $CDCl_3$, 298 K): 1.21 (s, 9H), 2.33-2.50 (m, 1H), 2.52-2.71 (m, 1H), 2.93-3.18 (m, 2H), 7.20 (d, *J* = 7.3 Hz, 1H), 7.29 (t, *J* = 7.5 Hz, 1H), 7.42-7.52 (m, 1H), 8.0 (d, *J* = 7.8 Hz, 1H) ppm; ^{19}F NMR (282 MHz, δ, $CDCl_3$, 298 K): -163.3 (dd, *J* = 19.4, 11.0 Hz) ppm; IR (film): $\tilde{\nu}$ = 2999, 2984, 2967, 2940, 2905, 2874, 2845, 1721, 1699, 1601, 1456, 1373, 1310, 1292, 1221, 1028, 1080, 999, 916, 835, 766, 739 cm^{-1}; HRMS (ESI): *m/z* calcd for $C_{15}H_{17}FO_3$: 282.15000 $[M+NH_4]^+$; found: 282.14944. The enantioselectivity was determined by HPLC (Chiralcel OD-H, eluent: hexane:i-PrOH = 200:1, 0.8 mL/min, 10°C, retention times: major 21.0 min, minor 22.9 min).

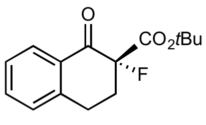

29

31. Obtained in 66% yield with *e.r.* = 89 : 11 upon reacting **30** (26 mg, 0.14 mmol) with NFSI (**18**). Analytical data are in accordance with those reported in literature.[8b] $[\alpha]_D^{24}$ (c = 0.15, CH_2Cl_2) = +44.0°; ^1H NMR (300 MHz, δ, $CDCl_3$, 298 K): 1.52 (s, 9H), 2.03-2.20 (m, 2H), 2.20-2.39 (m, 1H), 2.43-2.62 (m, *3*H) ppm; ^{13}C NMR (75 MHz, δ, $CDCl_3$, 298 K): 18.0 (d, *J* = 4.0 Hz), 27.9, 33.9 (d, *J* = 21.0 Hz), 35.7, 84.1, 94.3 (d, *J* = 199.9 Hz), 166.4 (d, *J* = 27.3 Hz), 208.1 (d, *J* =17.4 Hz) ppm; ^{19}F NMR (282 MHz, δ, $CDCl_3$, 298 K): -162.8 (dd, *J* = 22.0, 16.5 Hz) ppm; IR (film): $\tilde{\nu}$ = 2978, 2938, 2903, 1771, 1751, 1541, 1150 cm^{-1}; HRMS (ESI): *m/z* calcd for $C_{10}H_{15}FO_3$: 220.13435 $[M+NH_4]^+$; found: 220.13392. The enantioselectivity was determined by HPLC (Chiralpak AD-H, eluent: hexane:i-PrOH = 95:5, 0.75 mL/min, 10°C, retention times: minor 8.6 min, major 9.9 min).

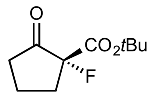

31

4. Copies of NMR Spectra of Key-Intermediates and Most Relevant Catalysts:

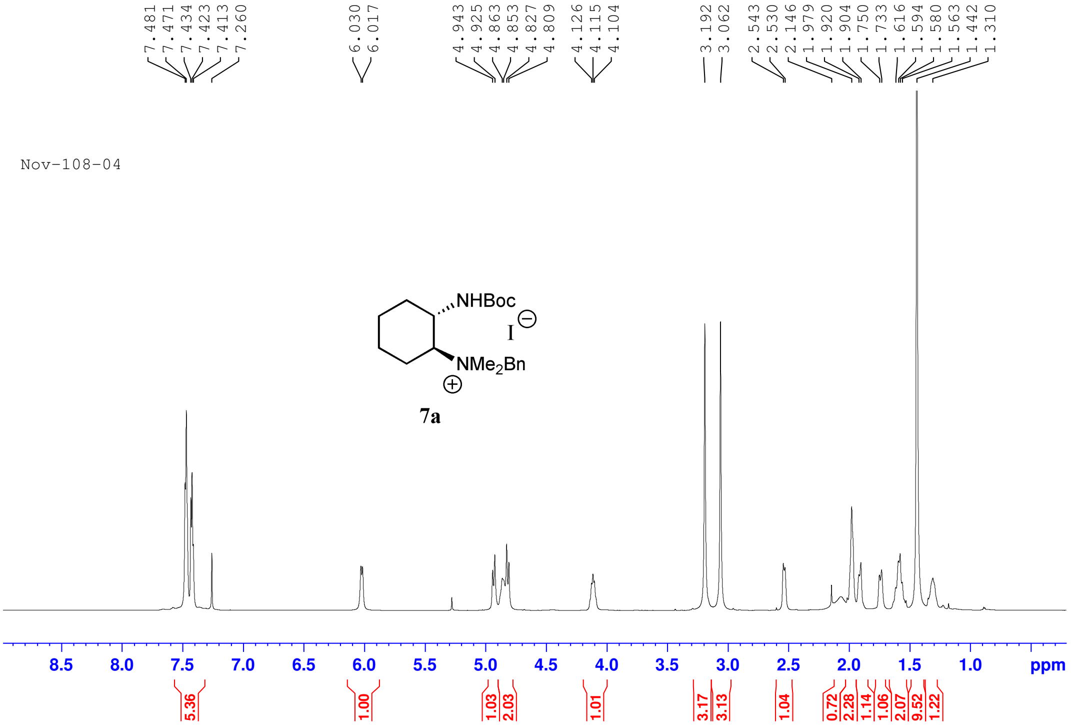

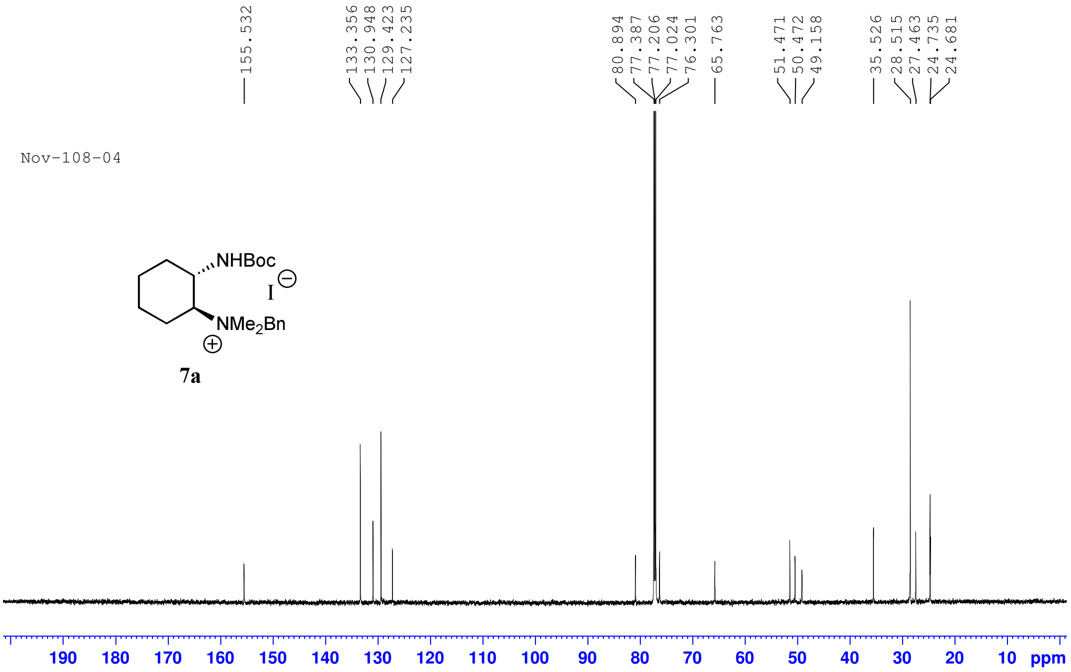

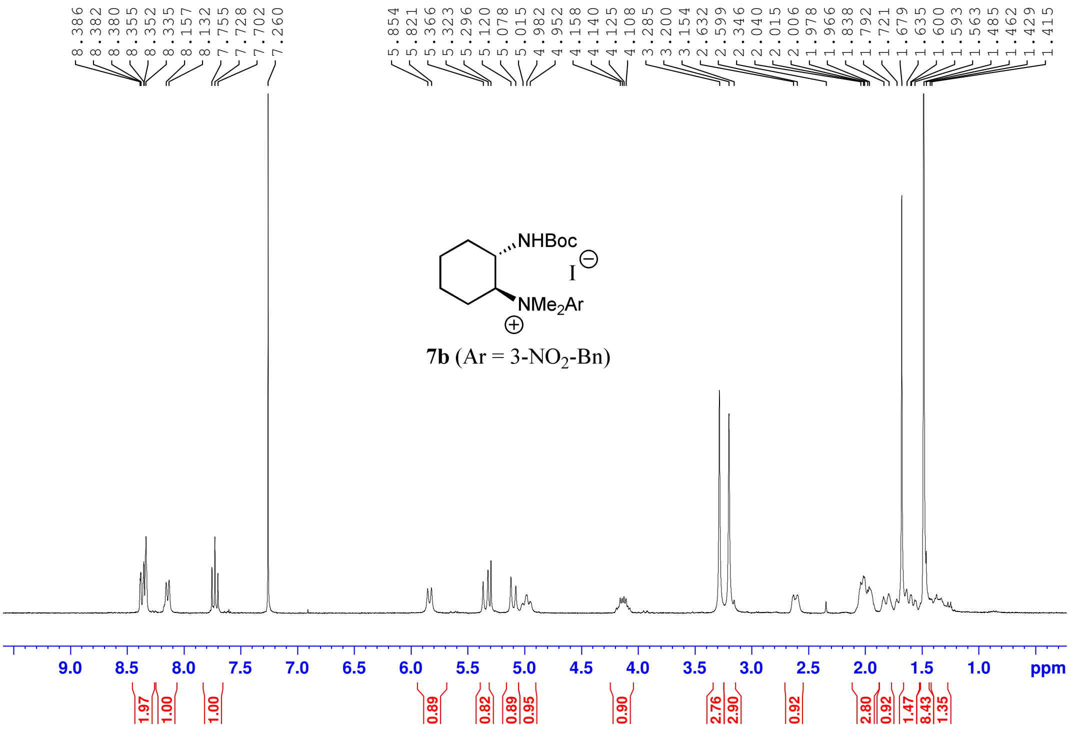
8.386
8.382
8.380
8.355
8.352
8.335
8.157
8.132
7.755
7.728
7.702
7.260
5.854
5.821
5.366
5.323
5.296
5.120
5.078
5.015
4.982
4.952
4.158
4.140
4.125
4.108
3.285
3.200
3.154
2.632
2.599
2.346
2.040
2.015
2.006
1.978
1.966
1.838
1.792
1.721
1.679
1.635
1.600
1.593
1.563
1.485
1.462
1.429
1.415
NHBoc
I
NMe2Ar
7b (Ar = 3-NO2-Bn)
9.0
8.5
8.0
7.5
7.0
6.5
6.0
5.5
5.0
4.5
4.0
3.5
3.0
2.5
2.0
1.5
1.0
ppm
1.97
1.00
1.00
0.89
0.82
0.89
0.95
0.90
2.76
2.90
0.92
2.80
0.92
1.47
8.43
1.35

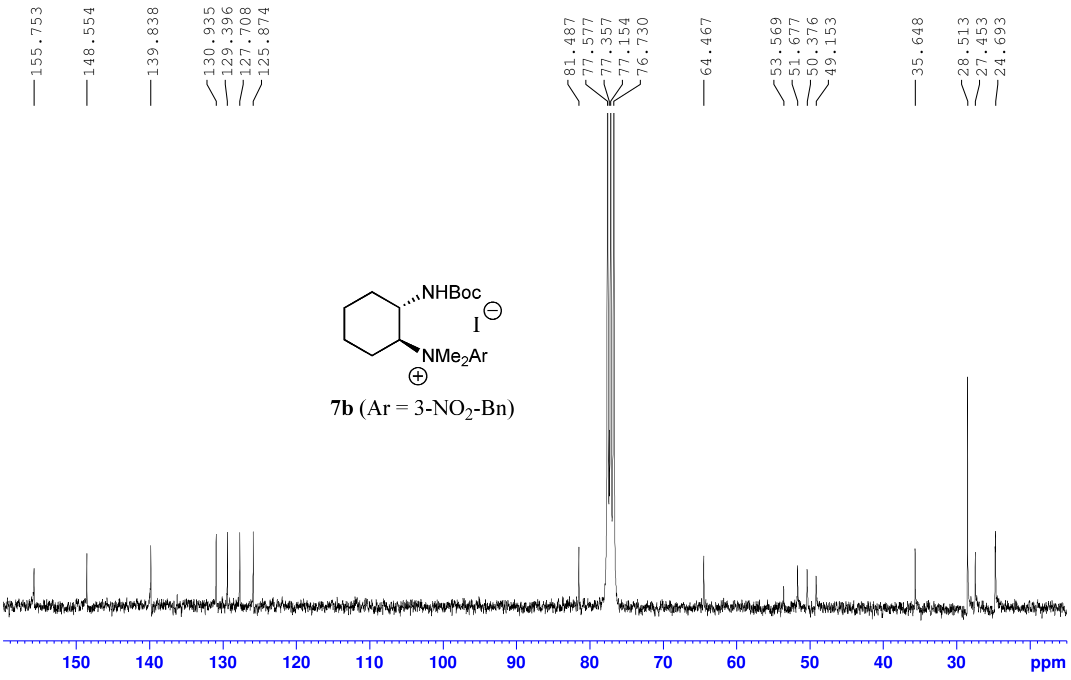
155.753
148.554
139.838
130.935
129.396
127.708
125.874
81.487
77.577
77.357
77.154
76.730
64.467
53.569
51.677
50.376
49.153
35.648
28.513
27.453
24.693
NHBoc
I
NMe2Ar
7b (Ar = 3-NO2-Bn)
150
140
130
120
110
100
90
80
70
60
50
40
30
ppm

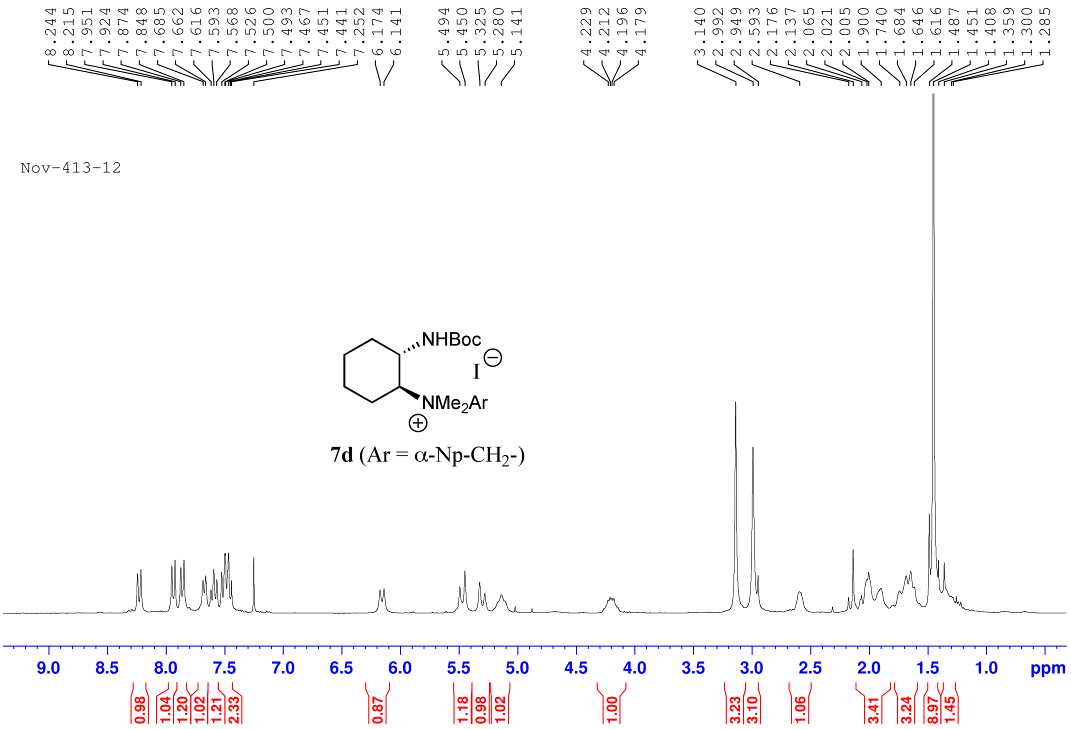
8.244 8.215 7.951 7.924 7.874 7.848 7.685 7.662 7.616 7.593 7.568 7.526 7.500 7.493 7.467 7.451 7.441 7.252 6.174 6.141
5.494 5.450 5.325 5.280 5.141
4.229 4.212 4.196 4.179
3.140 2.992 2.949 2.593 2.176 2.137 2.065 2.021 2.005 1.900 1.740 1.684 1.646 1.616 1.487 1.451 1.408 1.359 1.300 1.285
Nov-413-12
NHBoc
I
NMe2Ar
7d (Ar = α-Np-CH2-)
9.0 8.5 8.0 7.5 7.0 6.5 6.0 5.5 5.0 4.5 4.0 3.5 3.0 2.5 2.0 1.5 1.0 ppm
0.98 1.04 1.20 1.02 1.21 2.33 0.87 1.18 0.98 1.02 1.00 3.23 3.10 1.06 3.41 3.24 8.97 1.45

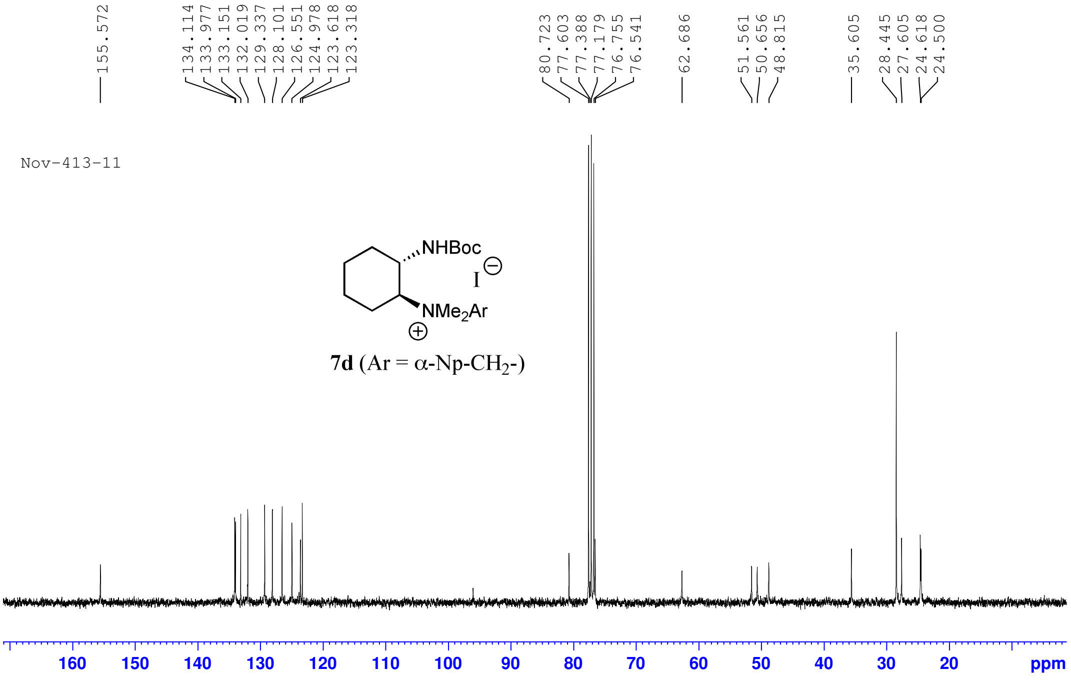
155.572
134.114 133.977 133.151 132.019 129.337 128.101 126.551 124.978 123.618 123.318
80.723 77.603 77.388 77.179 76.755 76.541
62.686
51.561 50.656 48.815
35.605 28.445 27.605 24.618 24.500
Nov-413-11
NHBoc
I
NMe2Ar
7d (Ar = α-Np-CH2-)
160 150 140 130 120 110 100 90 80 70 60 50 40 30 20 ppm

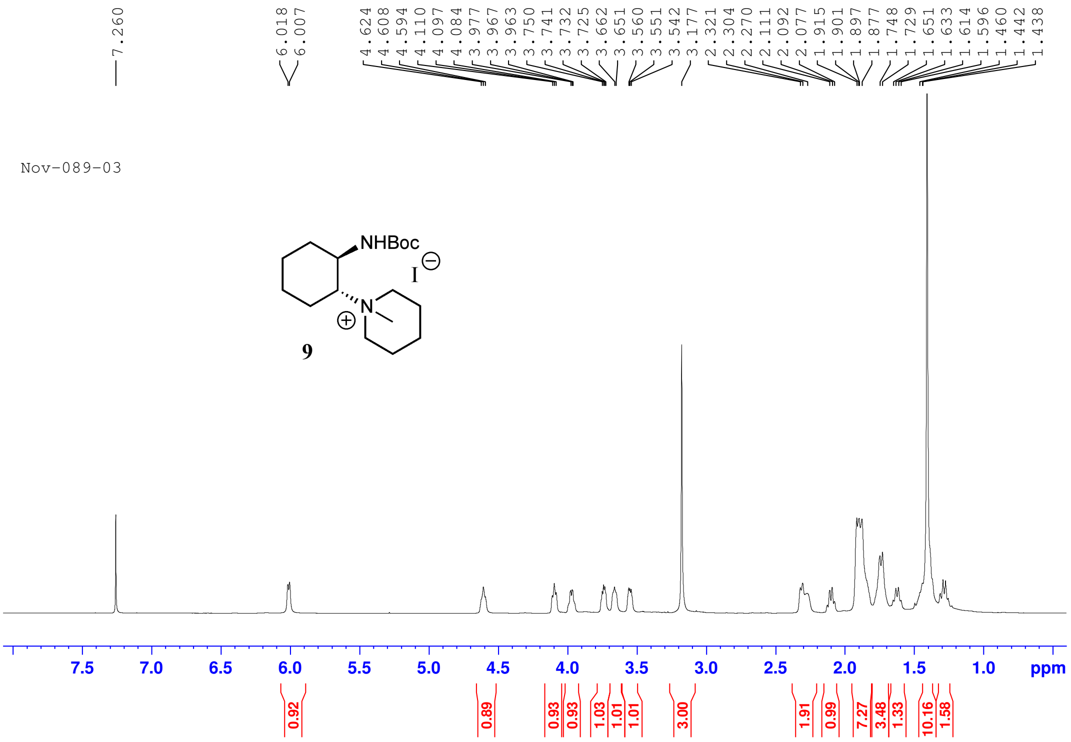
7.260
6.018
6.007
4.624
4.608
4.594
4.110
4.097
4.084
3.977
3.967
3.963
3.750
3.741
3.732
3.725
3.662
3.651
3.560
3.551
3.542
3.177
2.321
2.304
2.270
2.111
2.092
2.077
1.915
1.901
1.897
1.877
1.748
1.729
1.651
1.633
1.614
1.596
1.460
1.442
1.438
Nov-089-03
NHBoc
I
N
9
7.5 7.0 6.5 6.0 5.5 5.0 4.5 4.0 3.5 3.0 2.5 2.0 1.5 1.0 ppm
0.92 0.89 0.93 0.93 1.03 1.01 1.01 3.00 1.91 0.99 7.27 3.48 1.33 10.16 1.58

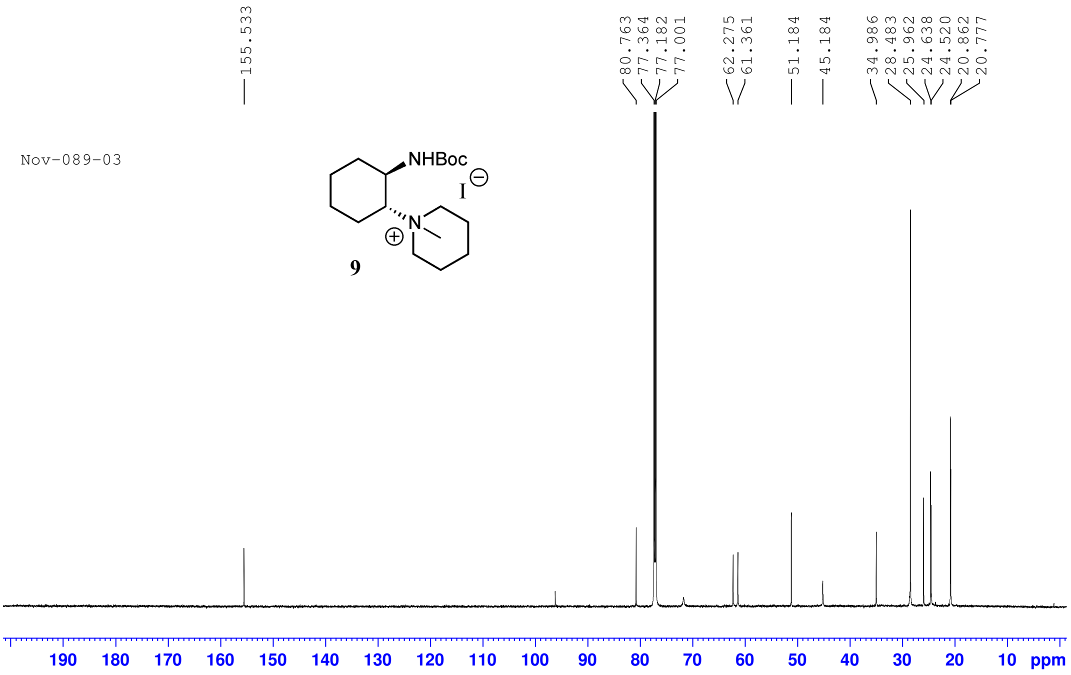
155.533
80.763
77.364
77.182
77.001
62.275
61.361
51.184
45.184
34.986
28.483
25.962
24.638
24.520
20.862
20.777
Nov-089-03
NHBoc
I
N
9
190 180 170 160 150 140 130 120 110 100 90 80 70 60 50 40 30 20 10 ppm

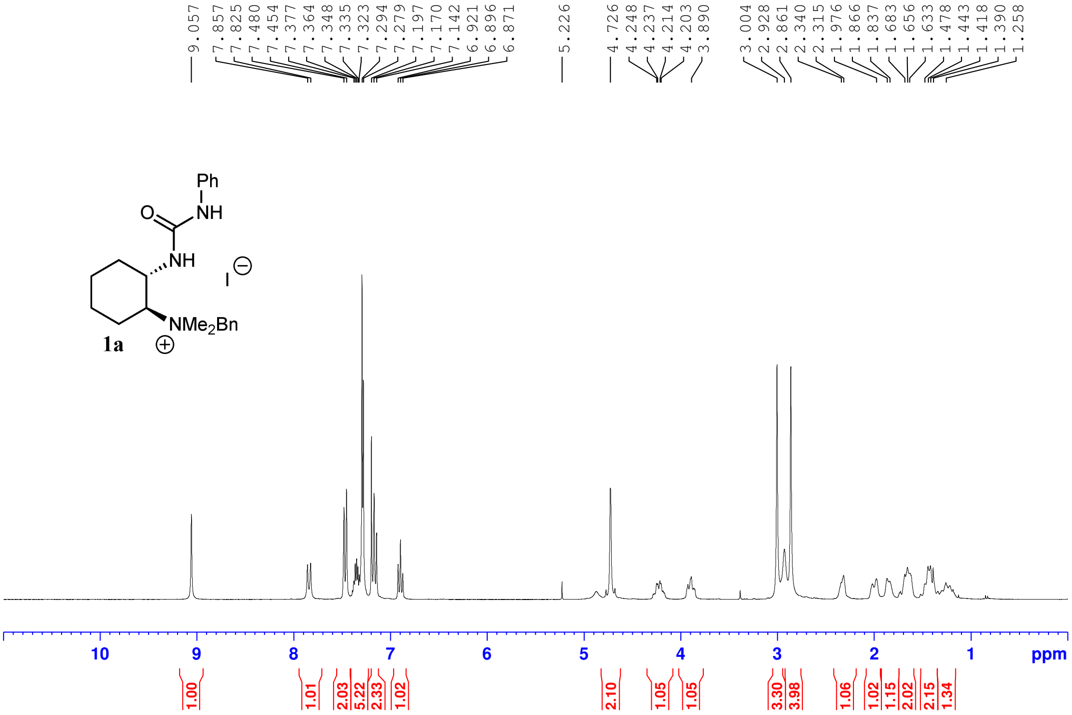
9.057
7.857
7.825
7.480
7.454
7.377
7.364
7.348
7.335
7.323
7.294
7.279
7.197
7.170
7.142
6.921
6.896
6.871
5.226
4.726
4.248
4.237
4.214
4.203
3.890
3.004
2.928
2.861
2.340
2.315
1.976
1.866
1.837
1.683
1.656
1.633
1.478
1.443
1.418
1.390
1.258
Ph
NH
O
NH
I
NMe2Bn
1a
10
9
8
7
6
5
4
3
2
1
ppm
1.00
1.01
2.03
5.22
2.33
1.02
2.10
1.05
1.05
3.30
3.98
1.06
1.02
1.15
2.02
2.15
1.34

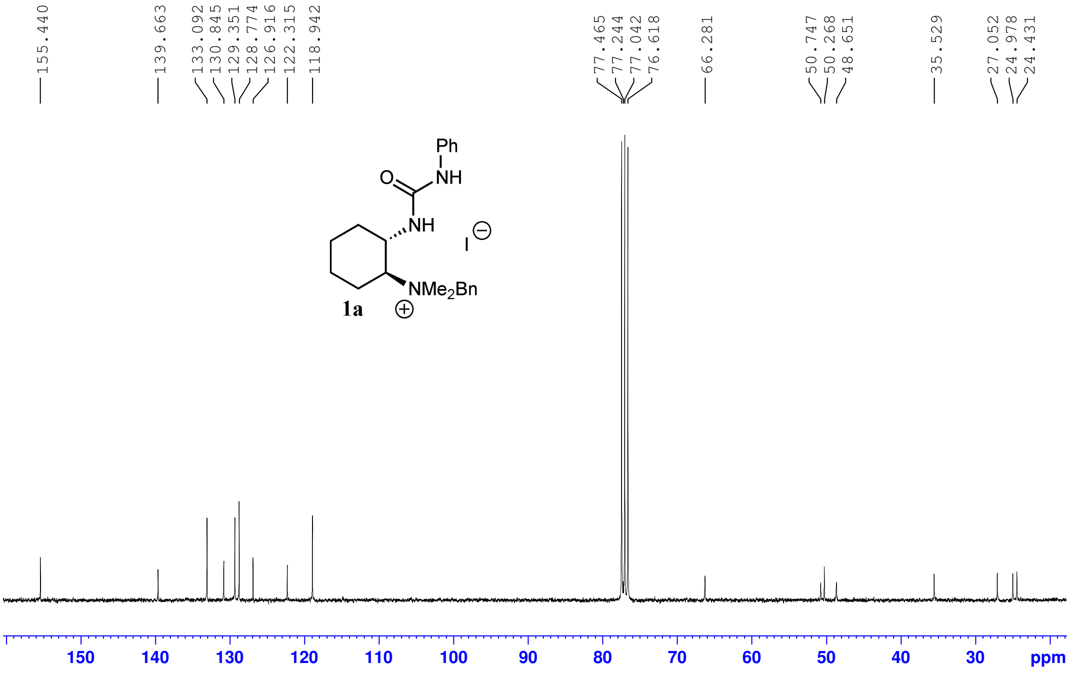
155.440
139.663
133.092
130.845
129.351
128.774
126.916
122.315
118.942
77.465
77.244
77.042
76.618
66.281
50.747
50.268
48.651
35.529
27.052
24.978
24.431
Ph
NH
O
NH
I
NMe2Bn
1a
150
140
130
120
110
100
90
80
70
60
50
40
30
ppm

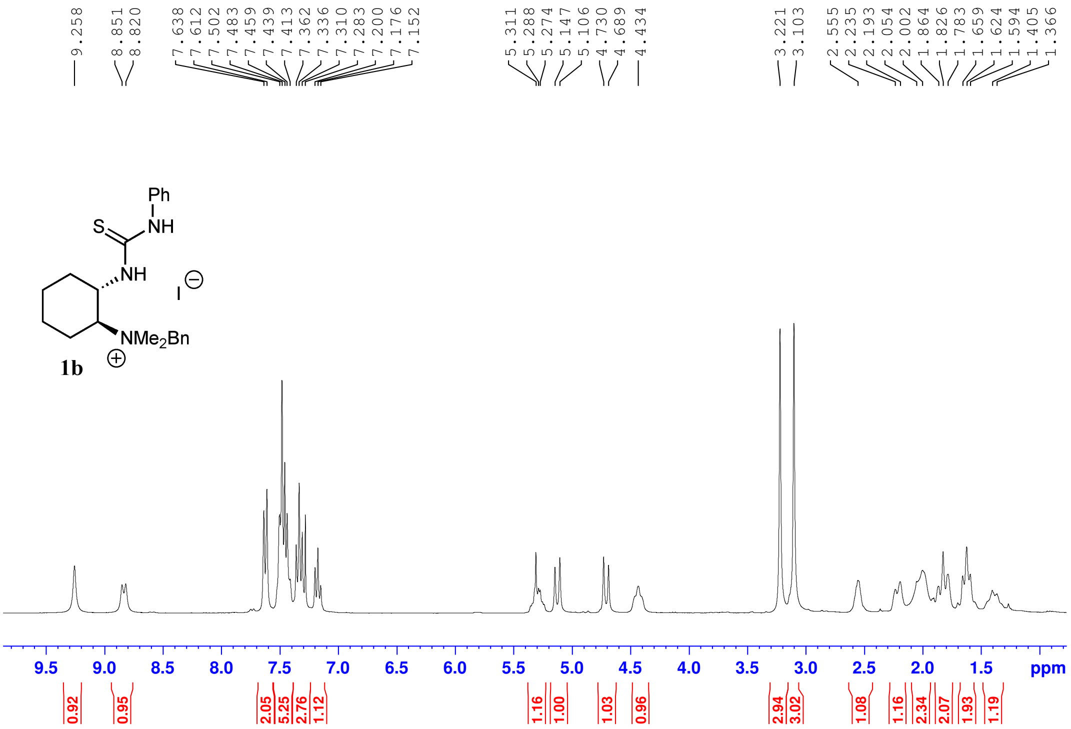
9.258
8.851
8.820
7.638
7.612
7.502
7.483
7.459
7.439
7.413
7.362
7.336
7.310
7.283
7.200
7.176
7.152
5.311
5.288
5.274
5.147
5.106
4.730
4.689
4.434
3.221
3.103
2.555
2.235
2.193
2.054
2.002
1.864
1.826
1.783
1.659
1.624
1.594
1.405
1.366
Ph
NH
S
NH
I
NMe2Bn
1b
9.5 9.0 8.5 8.0 7.5 7.0 6.5 6.0 5.5 5.0 4.5 4.0 3.5 3.0 2.5 2.0 1.5 ppm
0.92
0.95
2.05
5.25
2.76
1.12
1.16
1.00
1.03
0.96
2.94
3.02
1.08
1.16
2.34
2.07
1.93
1.19

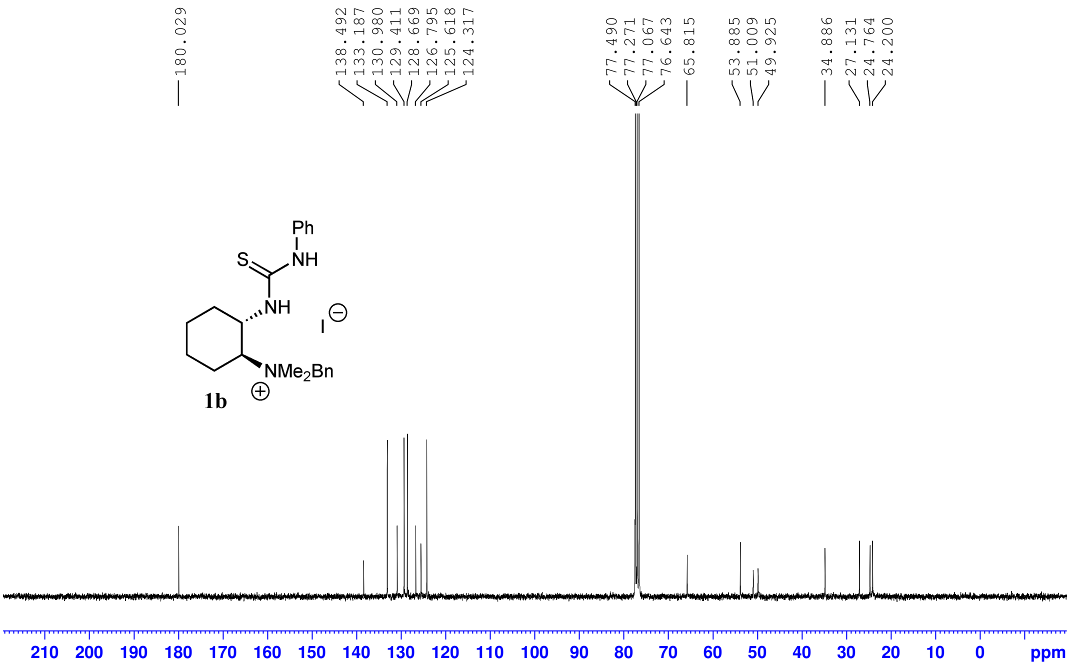
180.029
138.492
133.187
130.980
129.411
128.669
126.795
125.618
124.317
77.490
77.271
77.067
76.643
65.815
53.885
51.009
49.925
34.886
27.131
24.764
24.200
Ph
NH
S
NH
I
NMe2Bn
1b
210 200 190 180 170 160 150 140 130 120 110 100 90 80 70 60 50 40 30 20 10 0 ppm

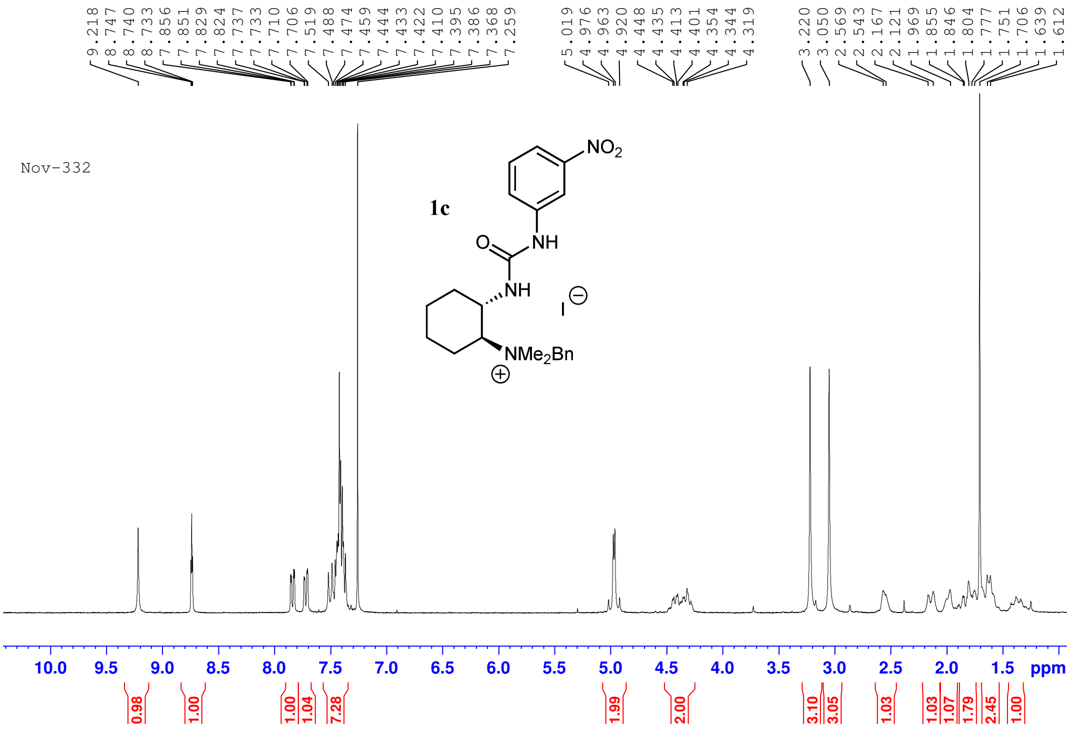
9.218 8.747 8.740 8.733 7.856 7.851 7.829 7.824 7.737 7.733 7.710 7.706 7.519 7.488 7.474 7.459 7.444 7.433 7.422 7.410 7.395 7.386 7.368 7.259
5.019 4.976 4.963 4.920 4.448 4.435 4.413 4.401 4.354 4.344 4.319
3.220 3.050 2.569 2.543 2.167 2.121 1.969 1.855 1.846 1.804 1.777 1.751 1.706 1.639 1.612
Nov-332
1c
NO2
NH
O
NH
I
NMe2Bn
10.0 9.5 9.0 8.5 8.0 7.5 7.0 6.5 6.0 5.5 5.0 4.5 4.0 3.5 3.0 2.5 2.0 1.5 ppm
0.98 1.00 1.00 1.04 7.28 1.99 2.00 3.10 3.05 1.03 1.03 1.07 1.79 2.45 1.00

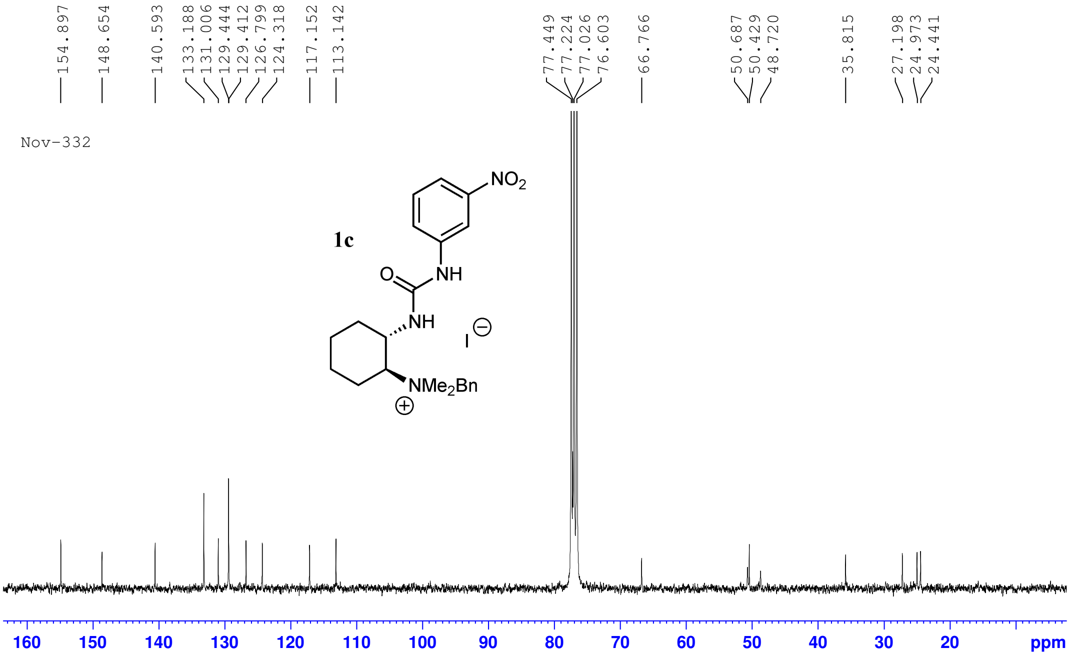
154.897 148.654 140.593 133.188 131.006 129.444 129.412 126.799 124.318 117.152 113.142
77.449 77.224 77.026 76.603 66.766 50.687 50.429 48.720 35.815 27.198 24.973 24.441
Nov-332
1c
NO2
NH
O
NH
I
NMe2Bn
160 150 140 130 120 110 100 90 80 70 60 50 40 30 20 ppm

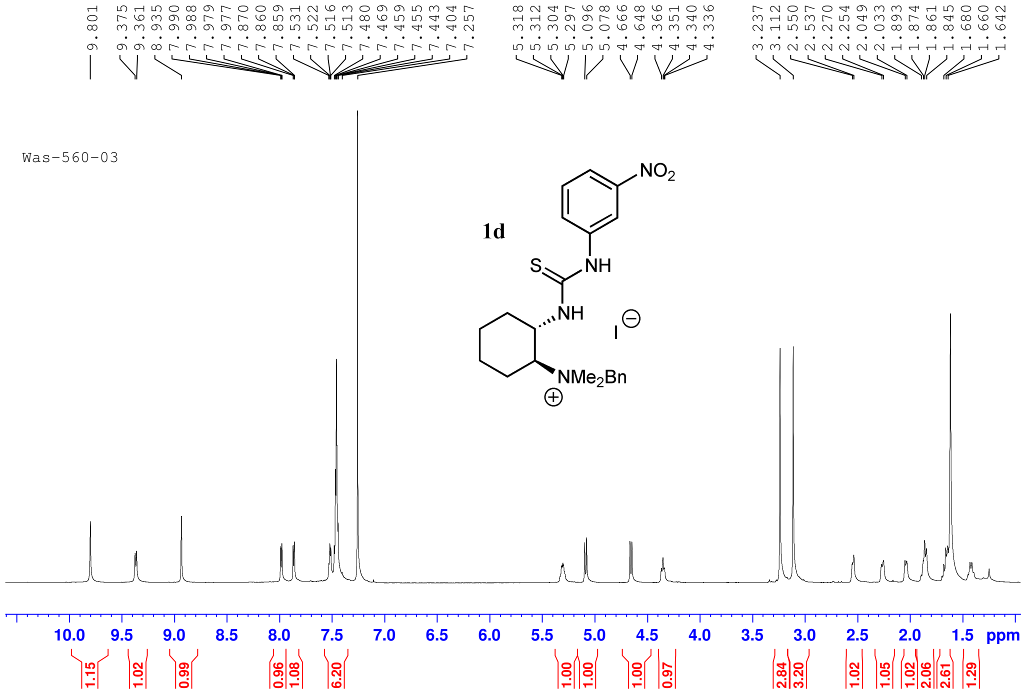

9.801
9.375
9.361
8.935
7.990
7.988
7.979
7.977
7.870
7.860
7.859
7.531
7.522
7.516
7.513
7.480
7.469
7.459
7.455
7.443
7.404
7.257
5.318
5.312
5.304
5.297
5.096
5.078
4.666
4.648
4.366
4.351
4.340
4.336
3.237
3.112
2.550
2.537
2.270
2.254
2.049
2.033
1.893
1.874
1.861
1.845
1.680
1.660
1.642
Was-560-03
1d
NO_2
NH
S
NH
I
NMe2Bn
10.0 9.5 9.0 8.5 8.0 7.5 7.0 6.5 6.0 5.5 5.0 4.5 4.0 3.5 3.0 2.5 2.0 1.5 ppm
1.15 1.02 0.99 0.96 1.08 6.20 1.00 1.00 1.00 0.97 2.84 3.20 1.02 1.05 1.02 2.06 2.61 1.29

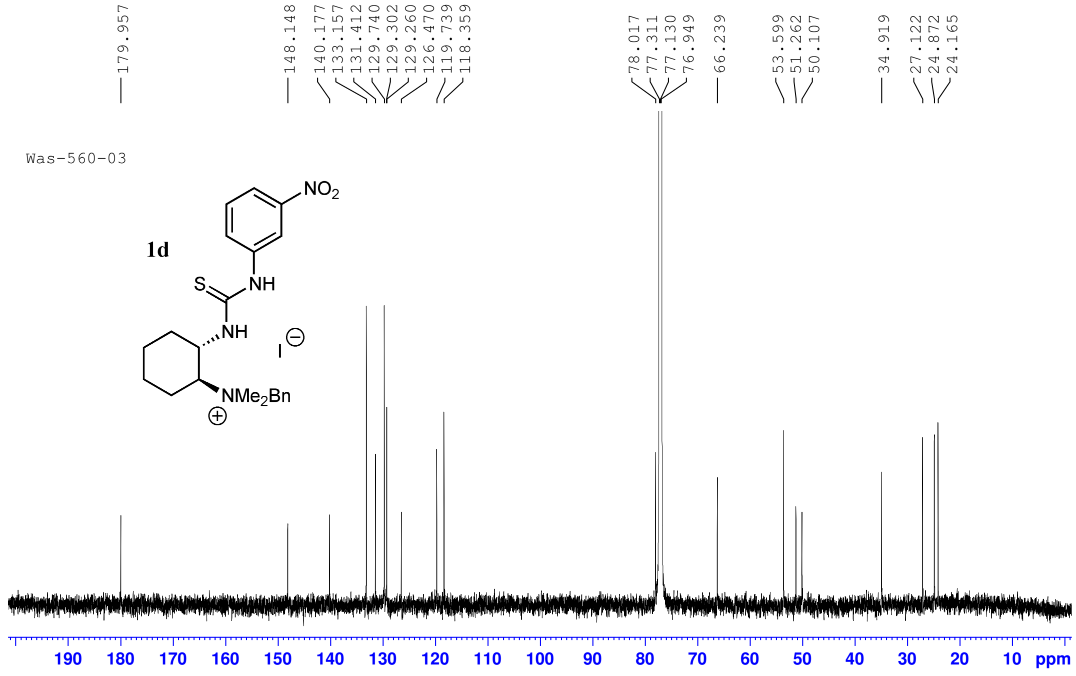

179.957
148.148
140.177
133.157
131.412
129.740
129.302
129.260
126.470
119.739
118.359
78.017
77.311
77.130
76.949
66.239
53.599
51.262
50.107
34.919
27.122
24.872
24.165
Was-560-03
1d
NO_2
NH
S
NH
I
NMe2Bn
190 180 170 160 150 140 130 120 110 100 90 80 70 60 50 40 30 20 10 ppm

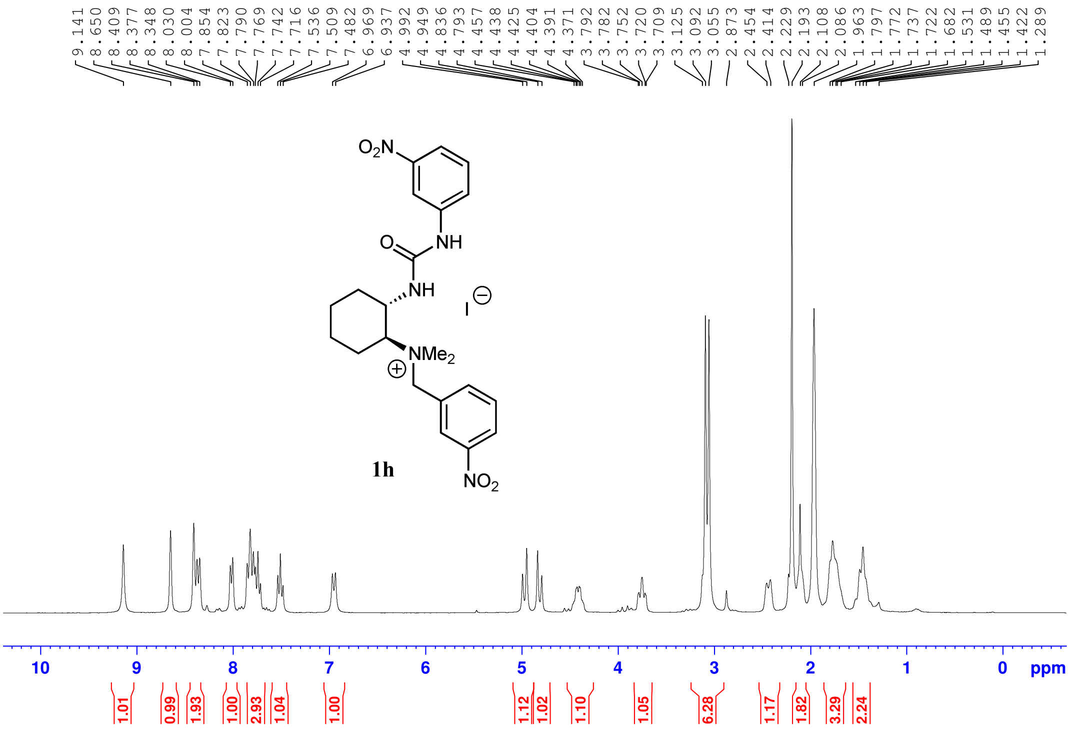
9.141 8.650 8.409 8.377 8.348 8.030 8.004 7.854 7.823 7.790 7.769 7.742 7.716 7.536 7.509 7.482 6.969 6.937 4.992 4.949 4.836 4.793 4.457 4.438 4.425 4.404 4.391 4.371 3.792 3.782 3.752 3.720 3.709 3.125 3.092 3.055 2.873 2.454 2.414 2.229 2.193 2.108 2.086 1.963 1.797 1.772 1.737 1.722 1.682 1.531 1.489 1.455 1.422 1.289
O_2N
NH
O
NH
I
NMe2
1h
NO_2
10 9 8 7 6 5 4 3 2 1 0 ppm
1.01 0.99 1.93 1.00 2.93 1.04 1.00 1.12 1.02 1.10 1.05 6.28 1.17 1.82 3.29 2.24

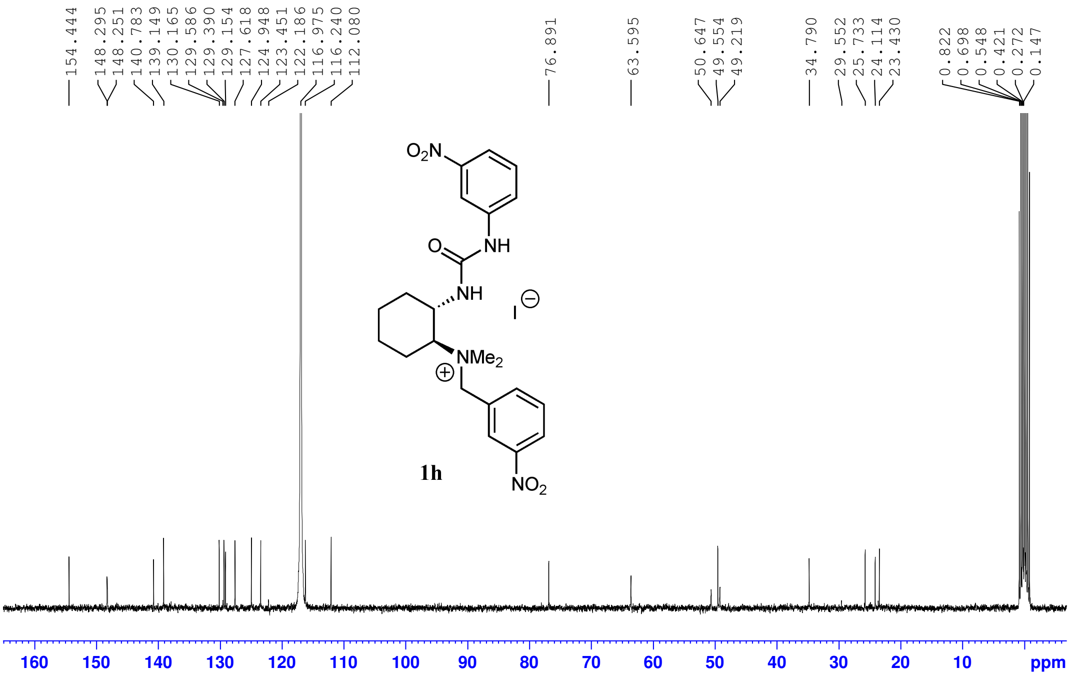
154.444 148.295 148.251 140.783 139.149 130.165 129.586 129.390 129.154 127.618 124.948 123.451 122.186 116.975 116.240 112.080
76.891
63.595
50.647 49.554 49.219
34.790 29.552 25.733 24.114 23.430
0.822 0.698 0.548 0.421 0.272 0.147
O_2N
NH
O
NH
I
NMe2
1h
NO_2
160 150 140 130 120 110 100 90 80 70 60 50 40 30 20 10 ppm

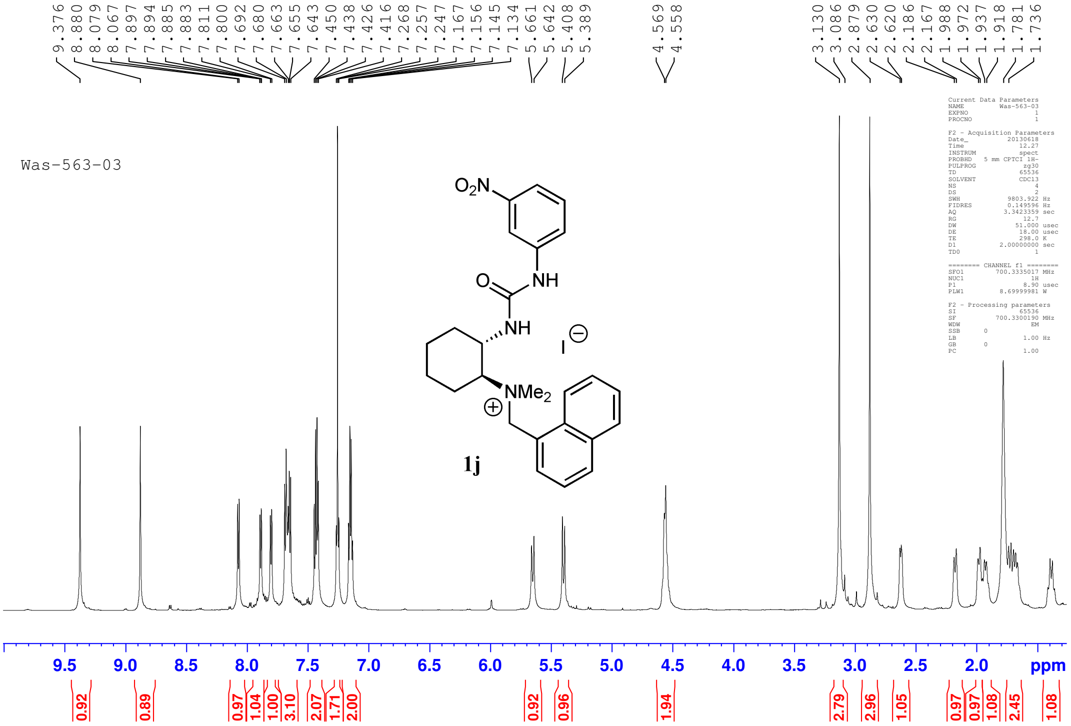

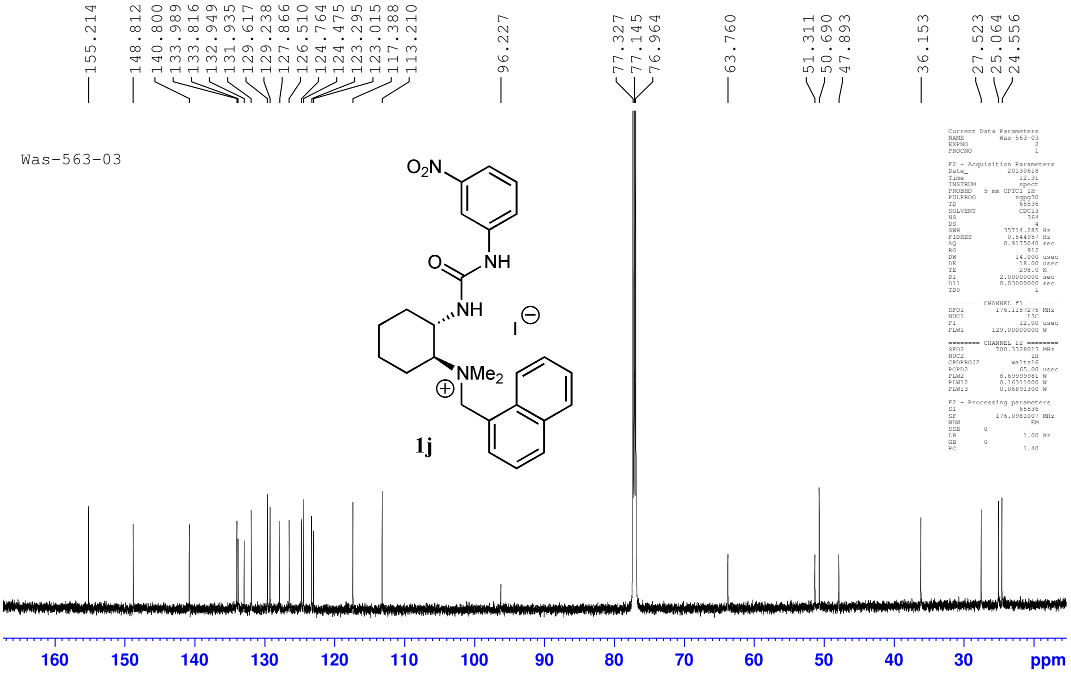

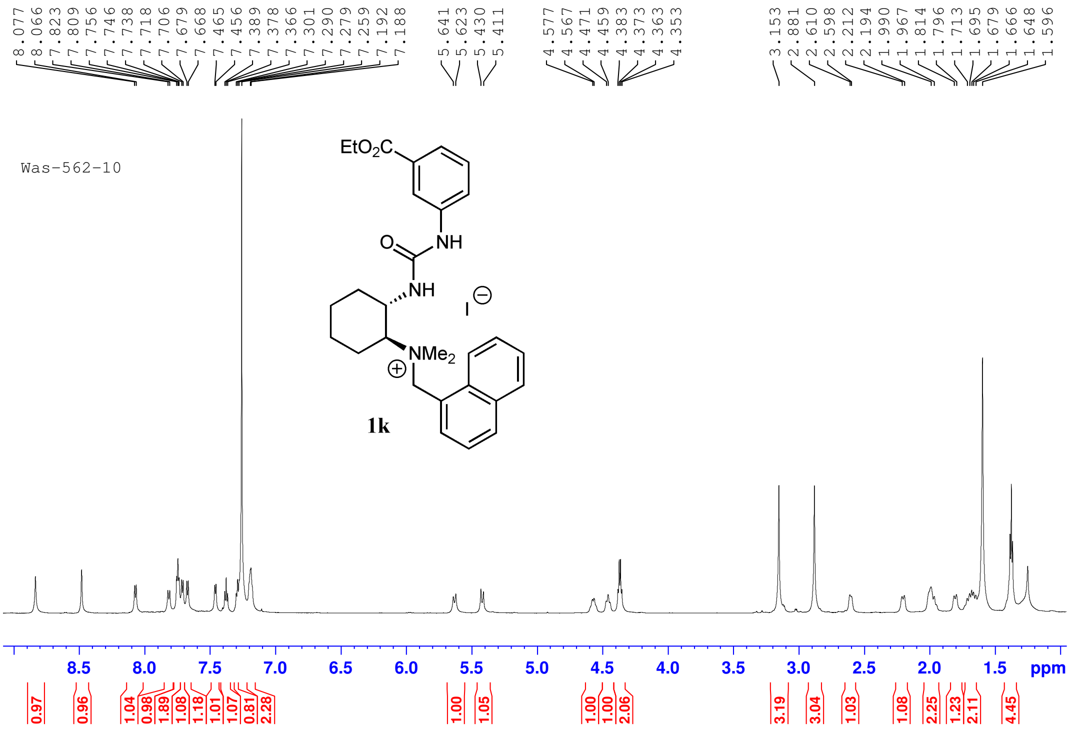
Was-562-10
1k
ppm

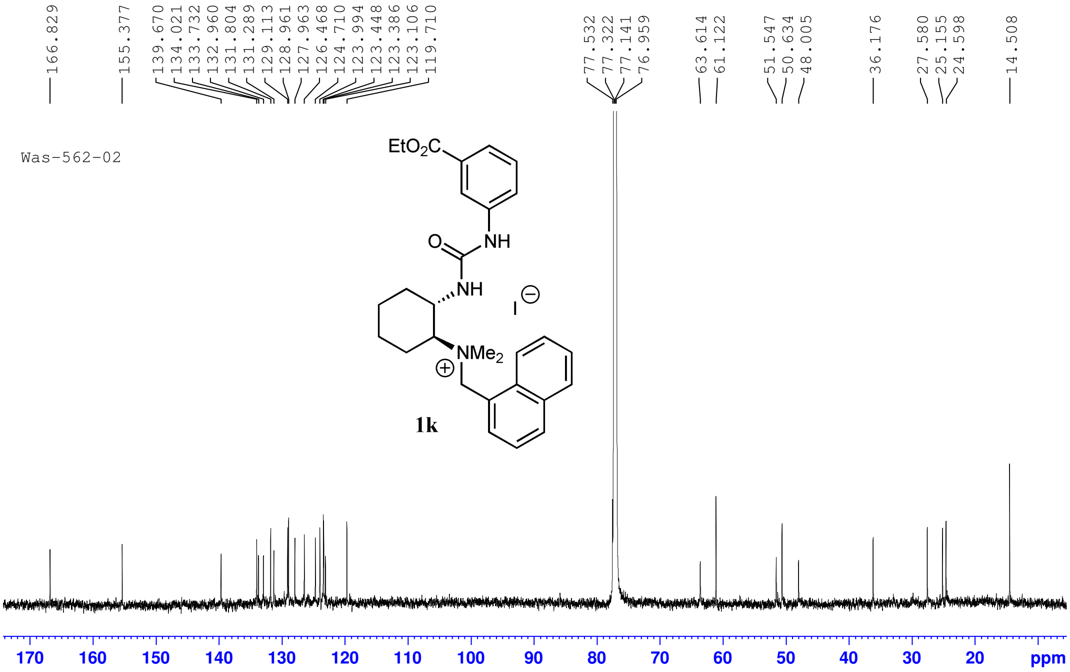
Was-562-02
1k
ppm

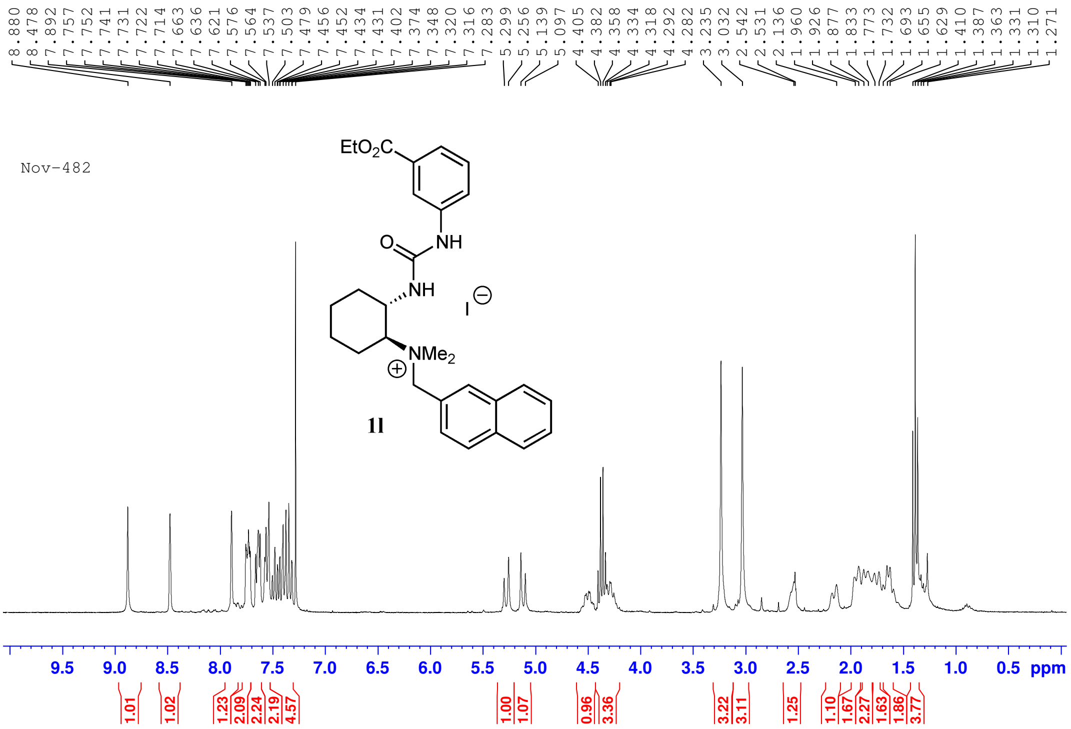
Nov-482
EtO2C
O
NH
NH
I
NMe2
1l
9.5
9.0
8.5
8.0
7.5
7.0
6.5
6.0
5.5
5.0
4.5
4.0
3.5
3.0
2.5
2.0
1.5
1.0
0.5
ppm

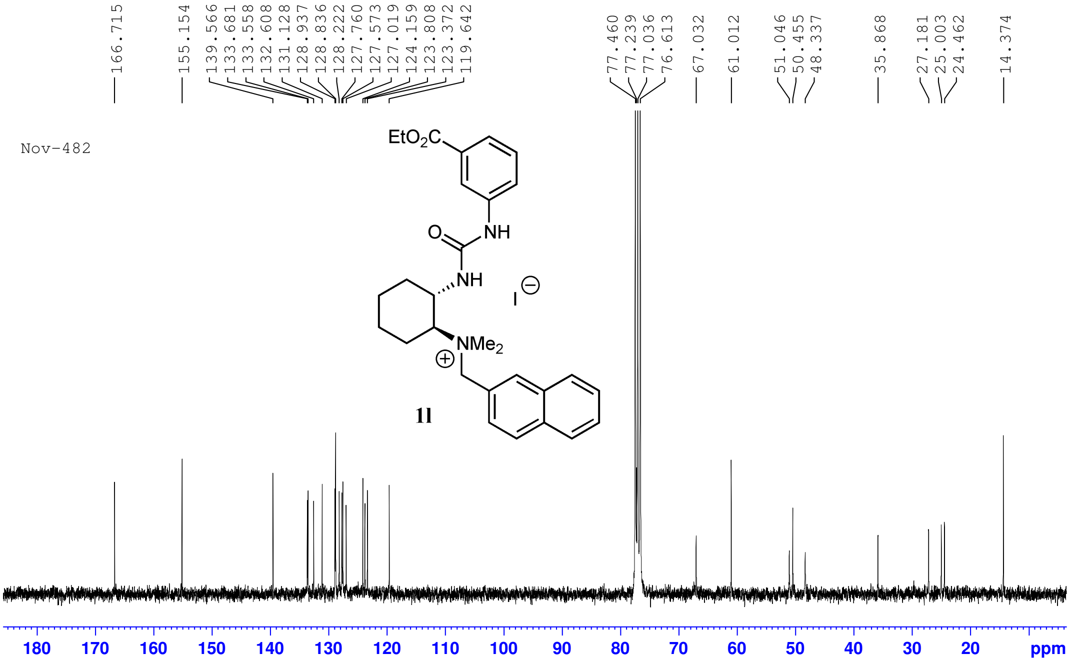
Nov-482
EtO2C
O
NH
NH
I
NMe2
1l
180
170
160
150
140
130
120
110
100
90
80
70
60
50
40
30
20
ppm

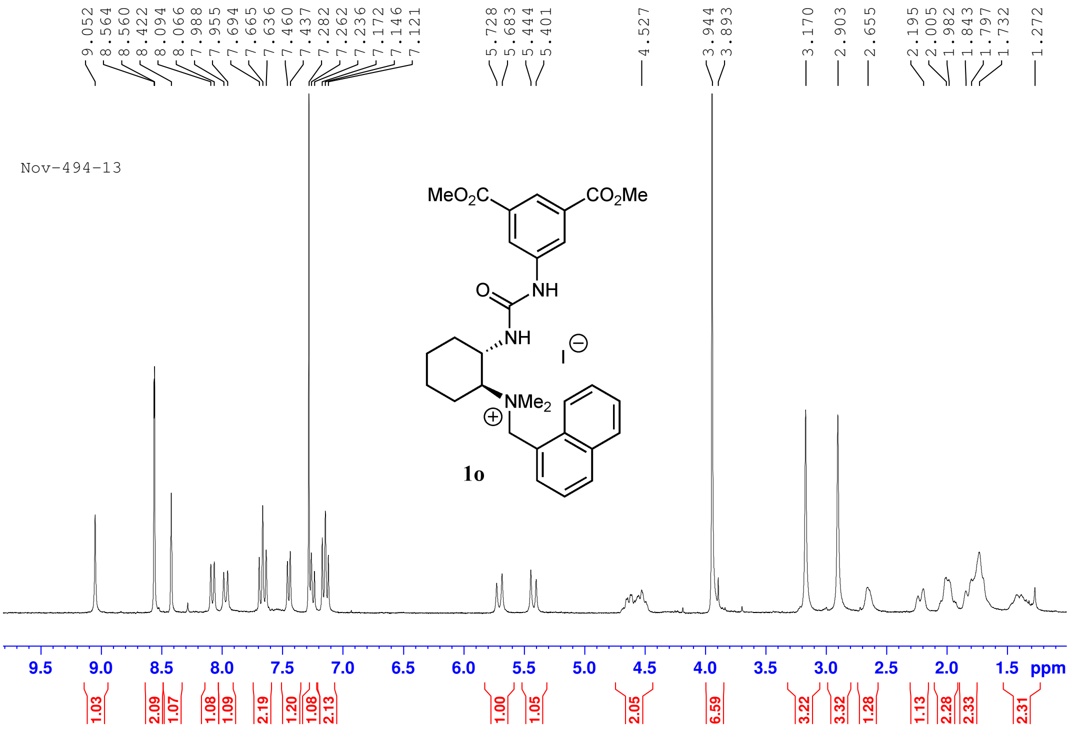
Nov-494-13
9.052 8.564 8.560 8.422 8.094 8.066 7.988 7.955 7.694 7.665 7.636 7.460 7.437 7.282 7.262 7.236 7.172 7.146 7.121
5.728 5.683 5.444 5.401
4.527
3.944 3.893
3.170
2.903
2.655
2.195 2.005 1.982 1.843 1.797 1.732
1.272
MeO2C
CO2Me
NH
O
NH
I
NMe2
1o
9.5 9.0 8.5 8.0 7.5 7.0 6.5 6.0 5.5 5.0 4.5 4.0 3.5 3.0 2.5 2.0 1.5 ppm
1.03 2.09 1.07 1.08 1.09 2.19 1.20 1.08 2.13 1.00 1.05 2.05 6.59 3.22 3.32 1.28 1.13 2.28 2.33 2.31

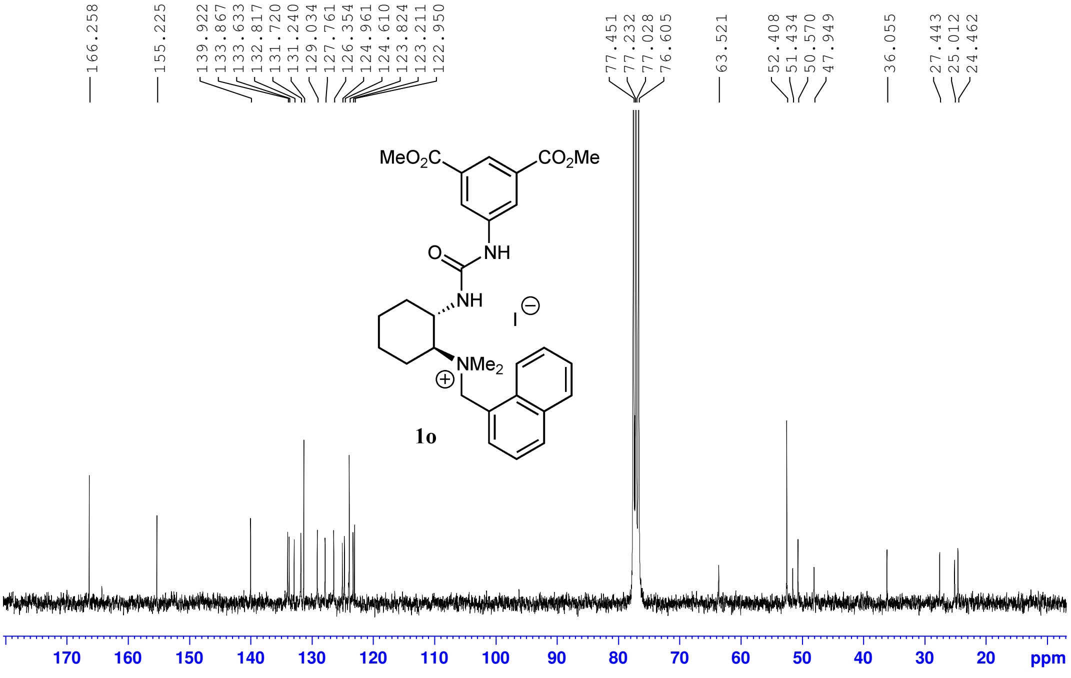
166.258
155.225
139.922 133.867 133.633 132.817 131.720 131.240 129.034 127.761 126.354 124.961 124.610 123.824 123.211 122.950
77.451 77.232 77.028 76.605
63.521
52.408 51.434 50.570 47.949
36.055
27.443 25.012 24.462
MeO2C
CO2Me
NH
O
NH
I
NMe2
1o
170 160 150 140 130 120 110 100 90 80 70 60 50 40 30 20 ppm

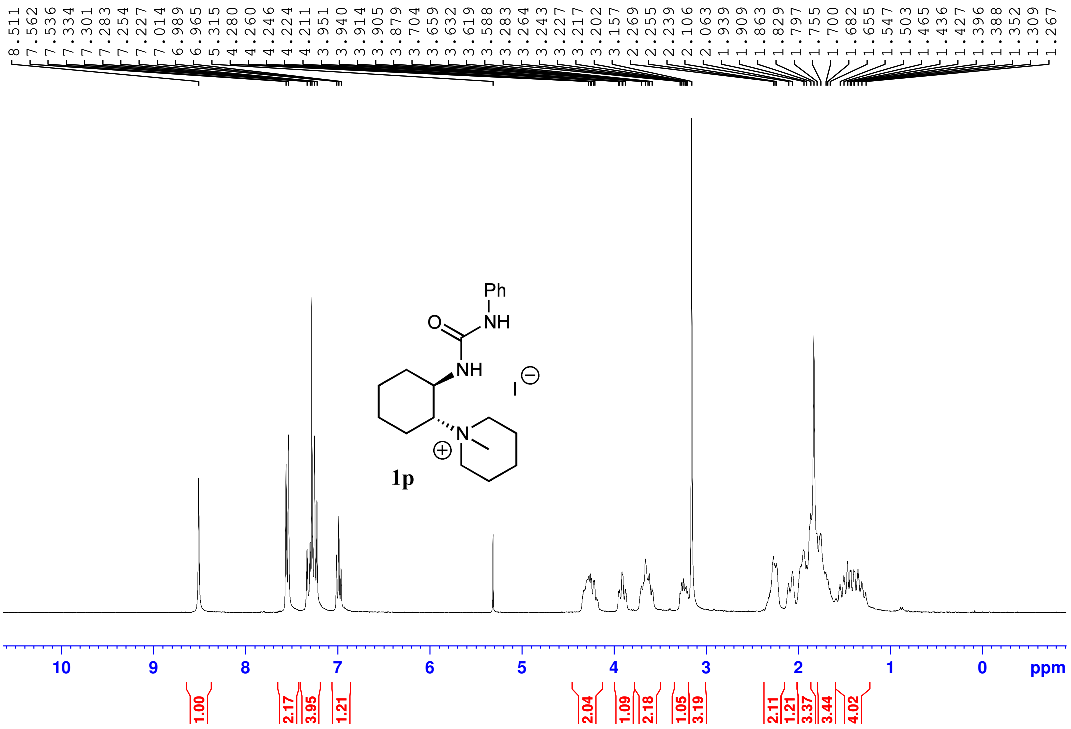
8.511 7.562 7.536 7.334 7.301 7.283 7.254 7.227 7.014 6.989 6.965 5.315 4.280 4.260 4.246 4.224 4.211 3.951 3.940 3.914 3.905 3.879 3.704 3.659 3.632 3.619 3.588 3.283 3.264 3.243 3.227 3.217 3.202 3.157 2.269 2.255 2.239 2.106 2.063 1.939 1.909 1.863 1.829 1.797 1.755 1.700 1.682 1.655 1.547 1.503 1.465 1.436 1.427 1.396 1.388 1.352 1.309 1.267
Ph
NH
O
NH
I
N
1p
10
9
8
7
6
5
4
3
2
1
0
ppm
1.00
2.17
3.95
1.21
2.04
1.09
2.18
1.05
3.19
2.11
1.21
3.37
3.44
4.02

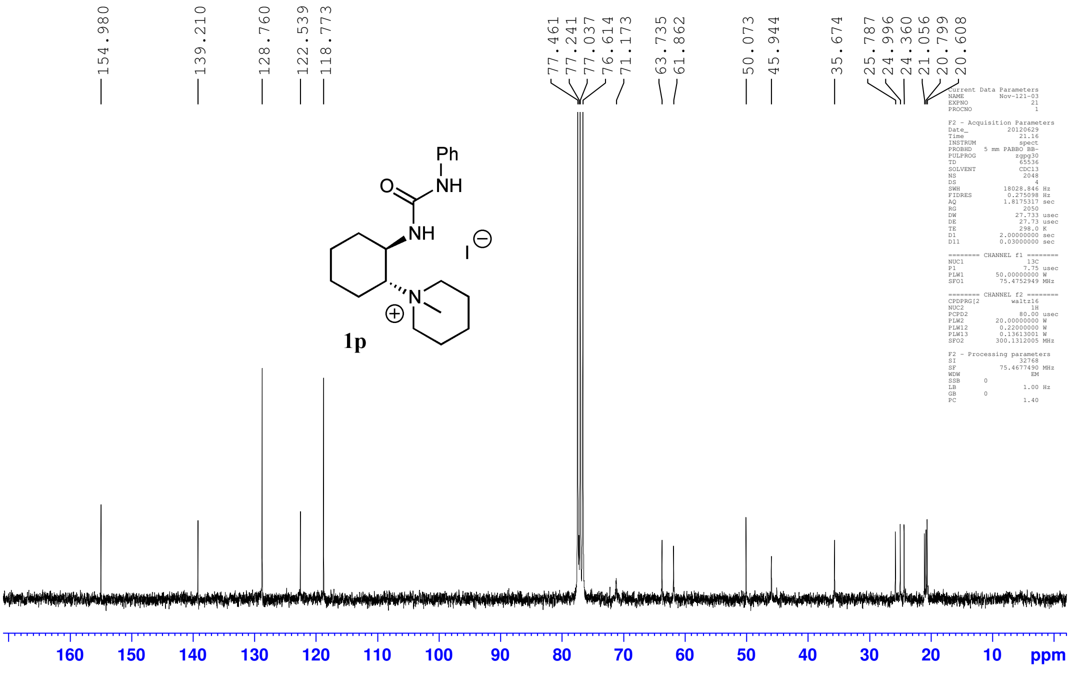
154.980
139.210
128.760
122.539
118.773
77.461 77.241 77.037 76.614 71.173
63.735 61.862
50.073
45.944
35.674
25.787 24.996 24.360 21.056 20.799 20.608
Ph
NH
O
NH
I
N
1p
160
150
140
130
120
110
100
90
80
70
60
50
40
30
20
10
ppm

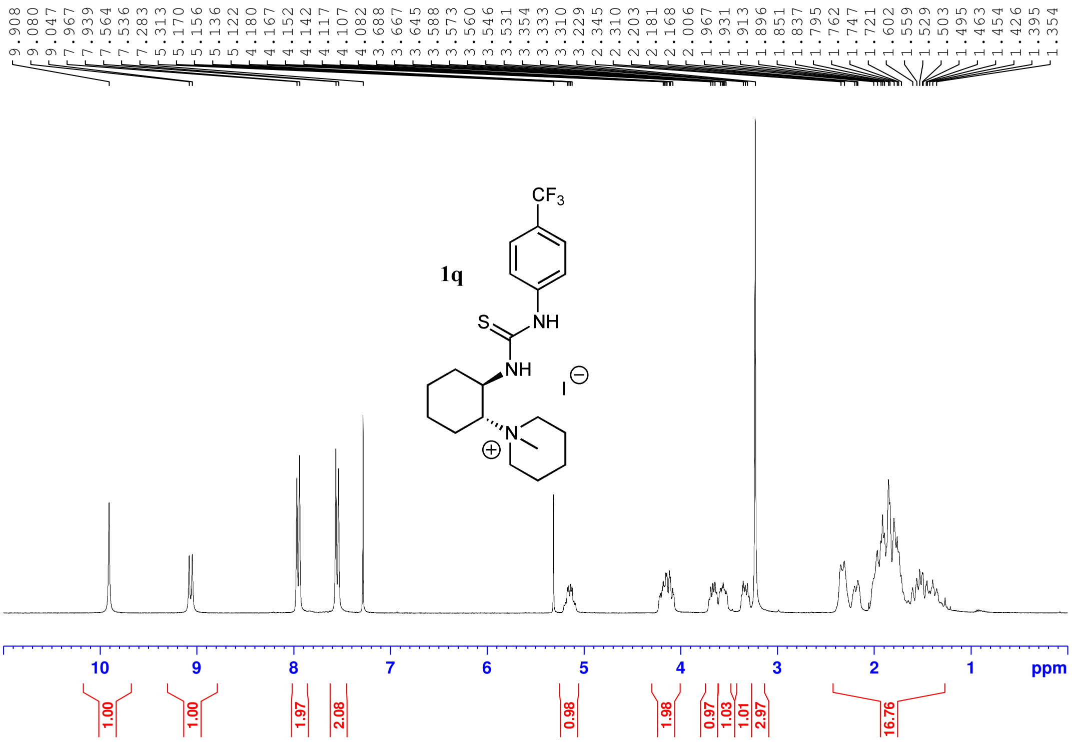
9.908 9.080 9.047 7.967 7.939 7.564 7.536 7.283 5.313 5.170 5.156 5.136 5.122 4.180 4.167 4.152 4.142 4.117 4.107 4.082 3.688 3.667 3.645 3.588 3.573 3.560 3.546 3.531 3.354 3.333 3.310 3.229 2.345 2.310 2.203 2.181 2.168 2.006 1.967 1.931 1.913 1.896 1.851 1.837 1.795 1.762 1.747 1.721 1.602 1.559 1.529 1.503 1.495 1.463 1.454 1.426 1.395 1.354
1q
CF3
S
NH
NH
I
N
ppm
1.00 1.00 1.97 2.08 0.98 1.98 0.97 1.03 1.01 2.97 16.76

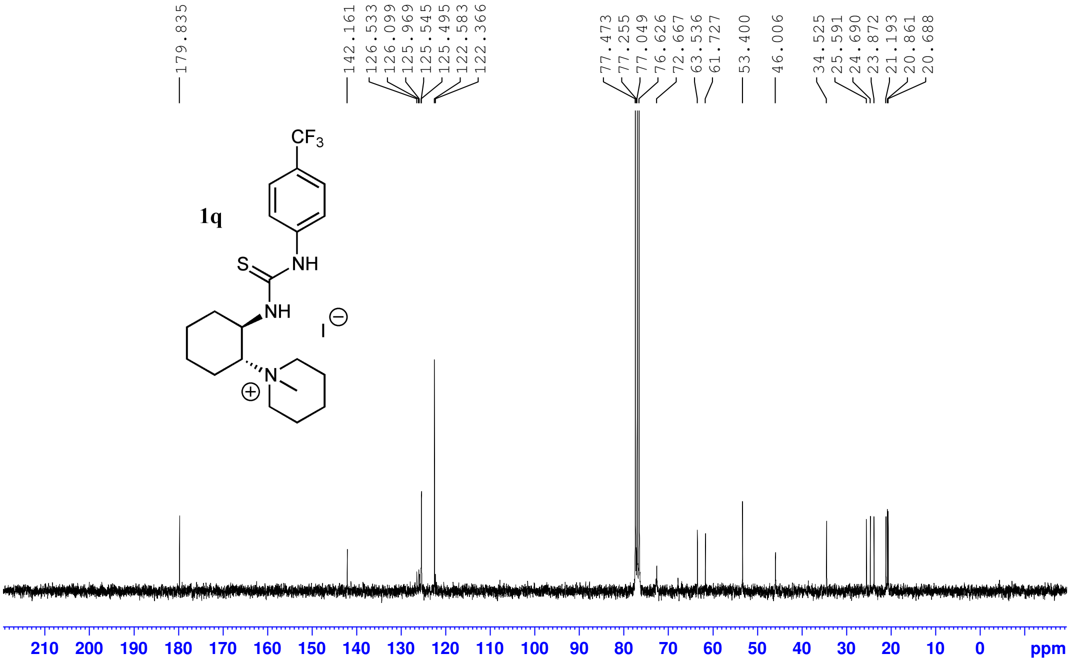
179.835
142.161 126.533 126.099 125.969 125.545 125.495 122.583 122.366
77.473 77.255 77.049 76.626 72.667 63.536 61.727 53.400 46.006
34.525 25.591 24.690 23.872 21.193 20.861 20.688
1q
CF3
S
NH
NH
I
N
ppm

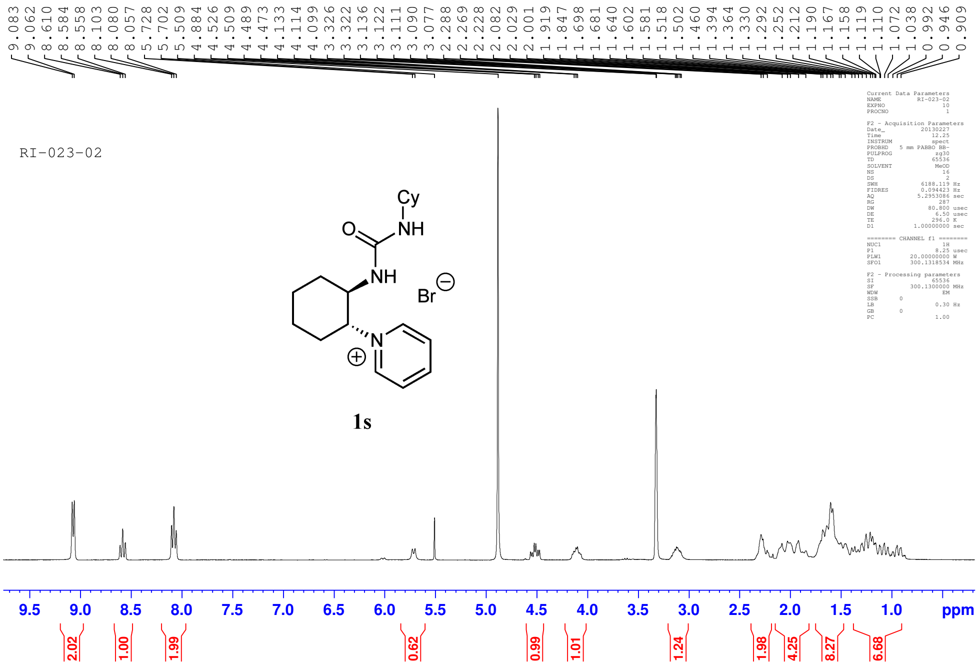
9.083 9.062 8.610 8.584 8.558 8.103 8.080 8.057 5.728 5.702 5.509 4.884 4.526 4.509 4.489 4.473 4.133 4.114 4.099 3.326 3.322 3.136 3.122 3.111 3.090 3.077 2.288 2.269 2.228 2.082 2.029 2.001 1.919 1.847 1.698 1.681 1.640 1.602 1.581 1.518 1.502 1.460 1.394 1.364 1.330 1.292 1.252 1.212 1.190 1.167 1.158 1.119 1.110 1.072 1.038 0.992 0.946 0.909
RI-023-02
Cy
NH
O
NH
Br
N
1s
9.5 9.0 8.5 8.0 7.5 7.0 6.5 6.0 5.5 5.0 4.5 4.0 3.5 3.0 2.5 2.0 1.5 1.0 ppm
2.02 1.00 1.99 0.62 0.99 1.01 1.24 1.98 4.25 8.27 6.68

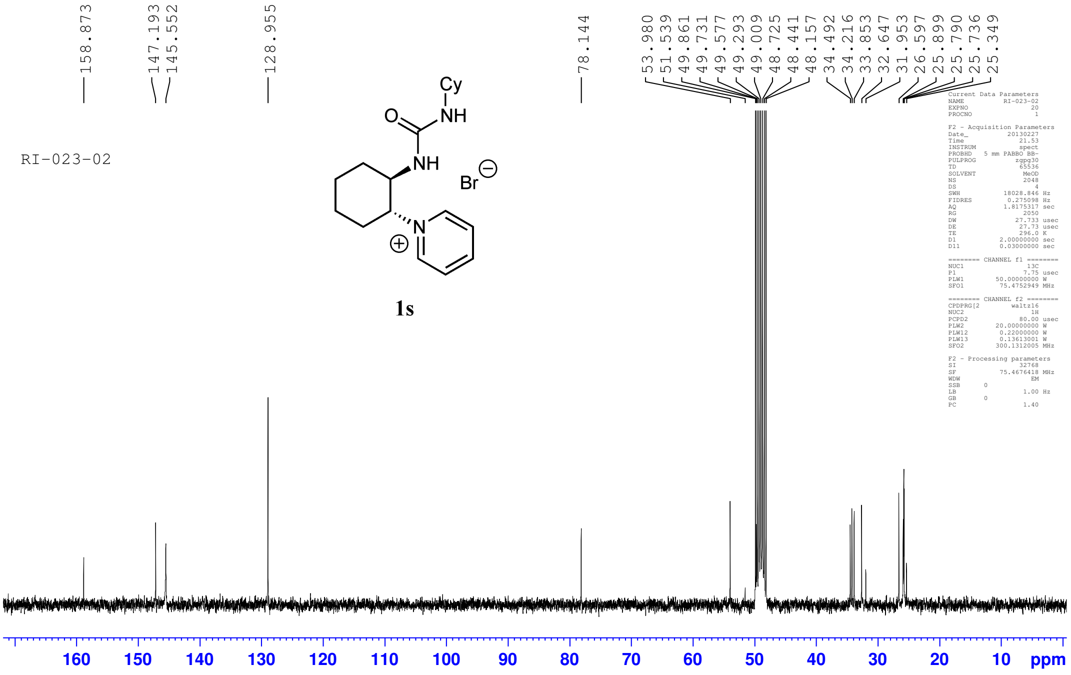
158.873
147.193 145.552
128.955
78.144
53.980 51.539 49.861 49.731 49.577 49.293 49.009 48.725 48.441 48.157
34.492 34.216 33.853 32.647 31.953 26.597 25.899 25.790 25.736 25.349
RI-023-02
Cy
NH
O
NH
Br
N
1s
160 150 140 130 120 110 100 90 80 70 60 50 40 30 20 10 ppm

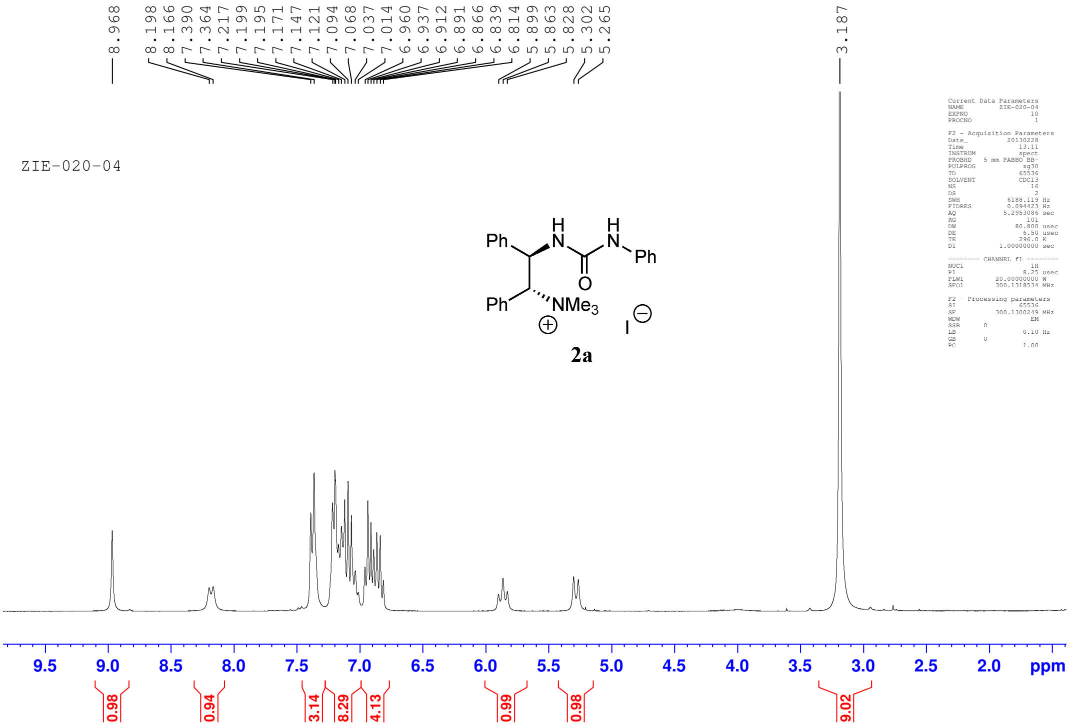
ZIE-020-04
8.968
8.198 8.166 7.390 7.364 7.217 7.199 7.195 7.171 7.147 7.121 7.094 7.068 7.037 7.014 6.960 6.937 6.912 6.891 6.866 6.839 6.814 5.899 5.863 5.828 5.302 5.265
3.187
Ph
H
N
H
N
Ph
O
Ph
NMe3
I
2a
9.5 9.0 8.5 8.0 7.5 7.0 6.5 6.0 5.5 5.0 4.5 4.0 3.5 3.0 2.5 2.0 ppm
0.98 0.94 3.14 8.29 4.13 0.99 0.98 9.02

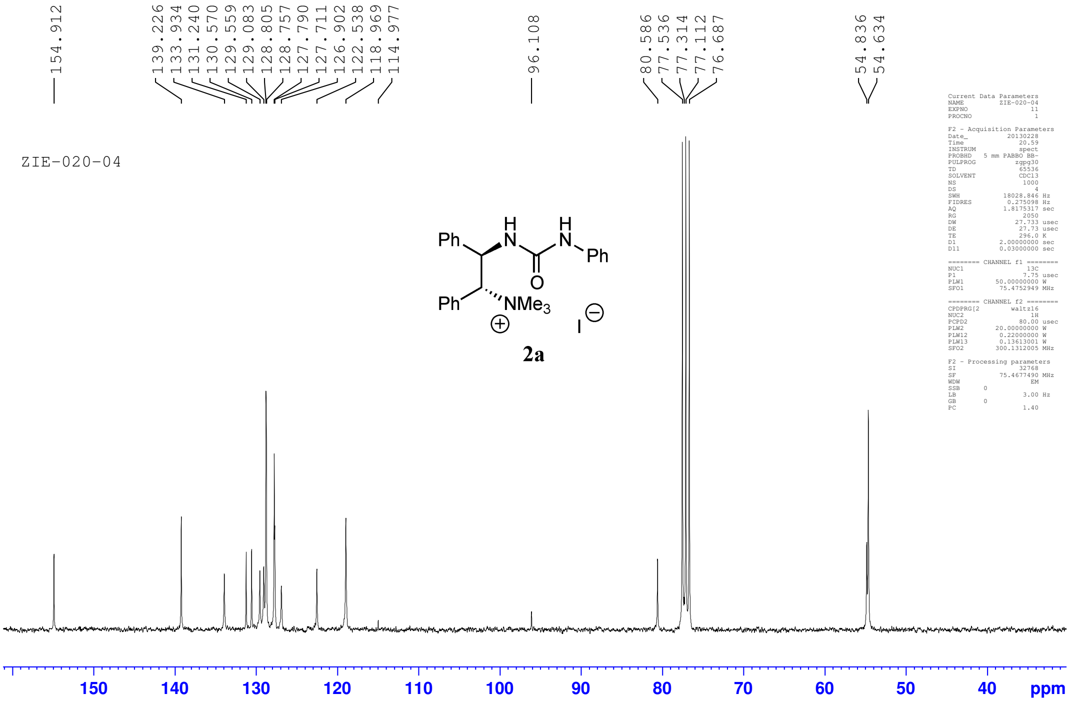
ZIE-020-04
154.912
139.226 133.934 131.240 130.570 129.559 129.083 128.805 128.757 127.790 127.711 126.902 122.538 118.969 114.977
96.108
80.586 77.536 77.314 77.112 76.687
54.836 54.634
Ph
H
N
H
N
Ph
O
Ph
NMe3
I
2a
150 140 130 120 110 100 90 80 70 60 50 40 ppm

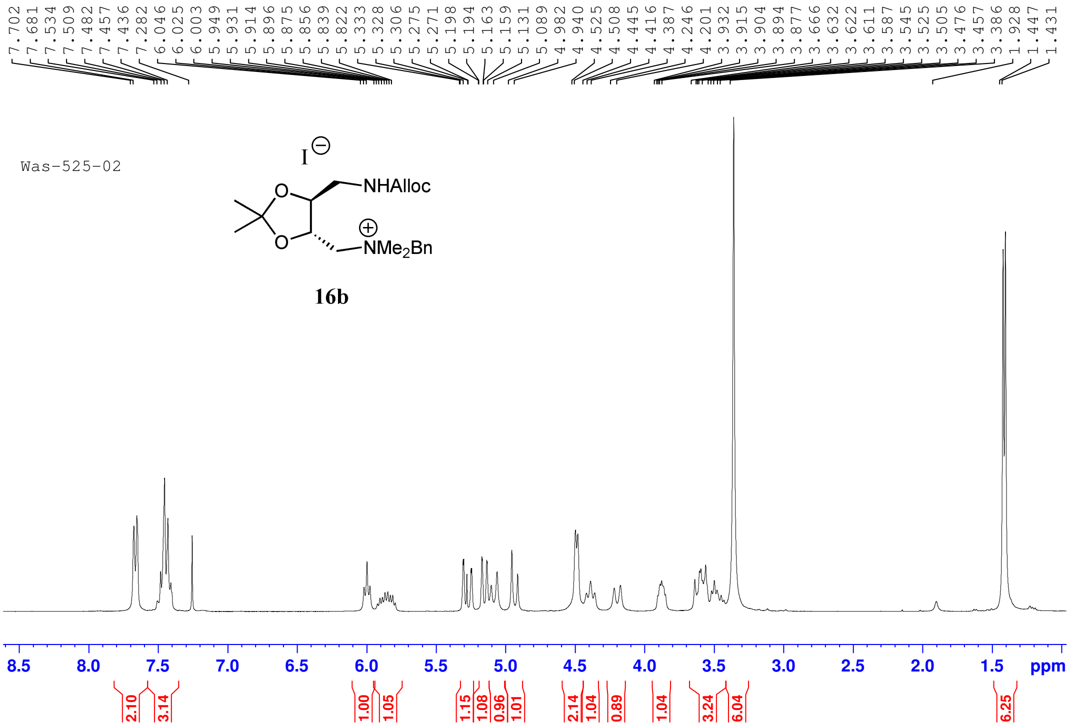
7.702 7.681 7.534 7.509 7.482 7.457 7.436 7.282 6.046 6.025 6.003 5.949 5.931 5.914 5.896 5.875 5.856 5.839 5.822 5.333 5.328 5.306 5.275 5.271 5.198 5.194 5.163 5.159 5.131 5.089 4.982 4.940 4.525 4.508 4.445 4.416 4.387 4.246 4.201 3.932 3.915 3.904 3.894 3.877 3.666 3.632 3.622 3.611 3.587 3.545 3.525 3.505 3.476 3.457 3.386 1.928 1.447 1.431
Was-525-02
I
NHAlloc
NMe2Bn
16b
8.5 8.0 7.5 7.0 6.5 6.0 5.5 5.0 4.5 4.0 3.5 3.0 2.5 2.0 1.5 ppm
2.10 3.14 1.00 1.05 1.15 1.08 0.96 1.01 2.14 1.04 0.89 1.04 3.24 6.04 6.25

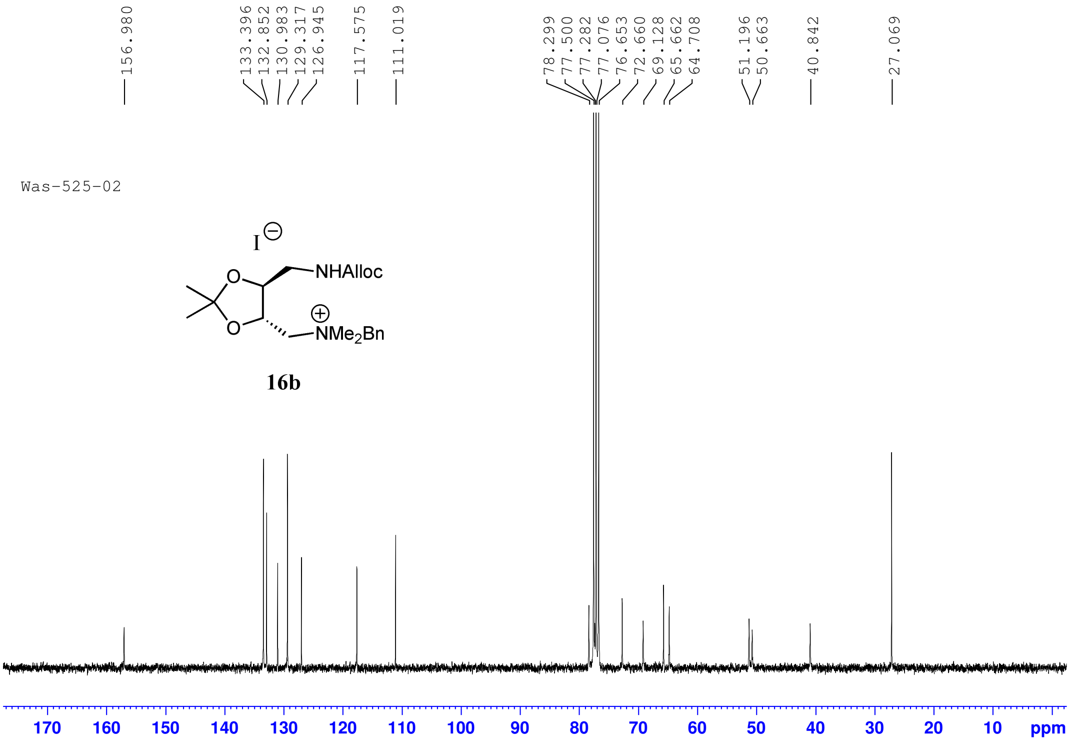
156.980
133.396 132.852 130.983 129.317 126.945
117.575
111.019
78.299 77.500 77.282 77.076 76.653 72.660 69.128 65.662 64.708
51.196 50.663
40.842
27.069
Was-525-02
I
NHAlloc
NMe2Bn
16b
170 160 150 140 130 120 110 100 90 80 70 60 50 40 30 20 10 ppm

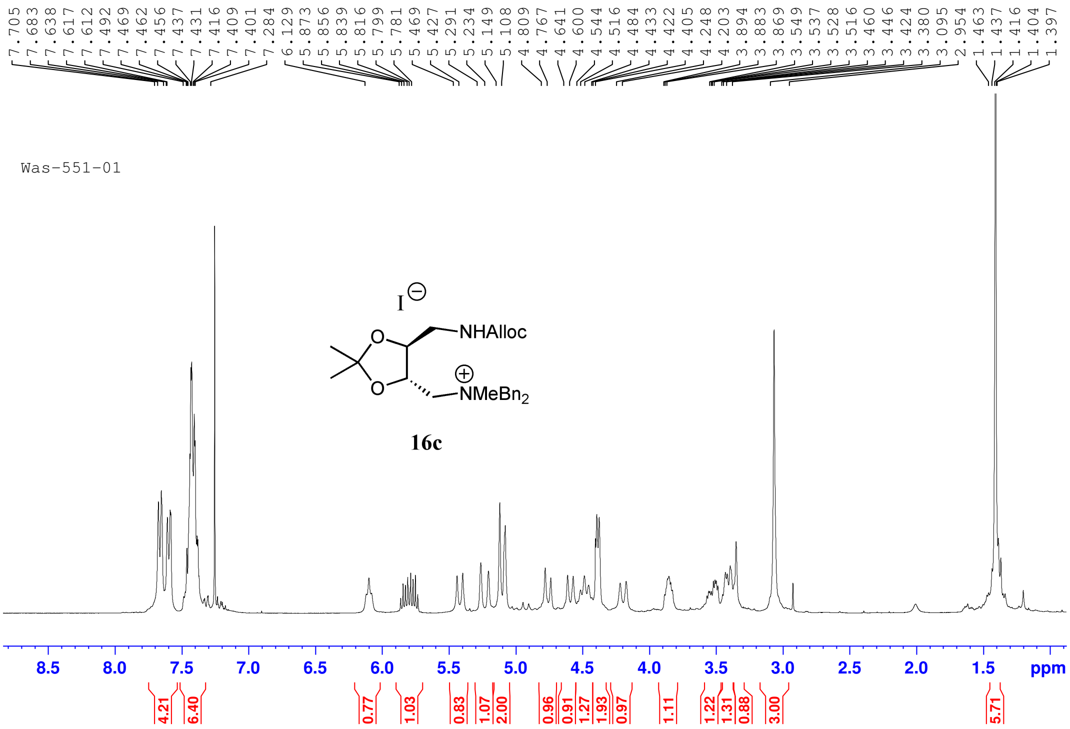
7.705 7.683 7.638 7.617 7.612 7.492 7.469 7.462 7.456 7.437 7.431 7.416 7.409 7.401 7.284 6.129 5.873 5.856 5.839 5.816 5.799 5.781 5.469 5.427 5.291 5.234 5.149 5.108 4.809 4.767 4.641 4.600 4.544 4.516 4.484 4.433 4.422 4.405 4.248 4.203 3.894 3.883 3.869 3.549 3.537 3.528 3.516 3.460 3.446 3.424 3.380 3.095 2.954 1.463 1.437 1.416 1.404 1.397
Was-551-01
I
NHAlloc
NMeBn2
16c
8.5 8.0 7.5 7.0 6.5 6.0 5.5 5.0 4.5 4.0 3.5 3.0 2.5 2.0 1.5 ppm
4.21 6.40 0.77 1.03 0.83 1.07 2.00 0.96 0.91 1.27 1.93 0.97 1.11 1.22 1.31 0.88 3.00 5.71

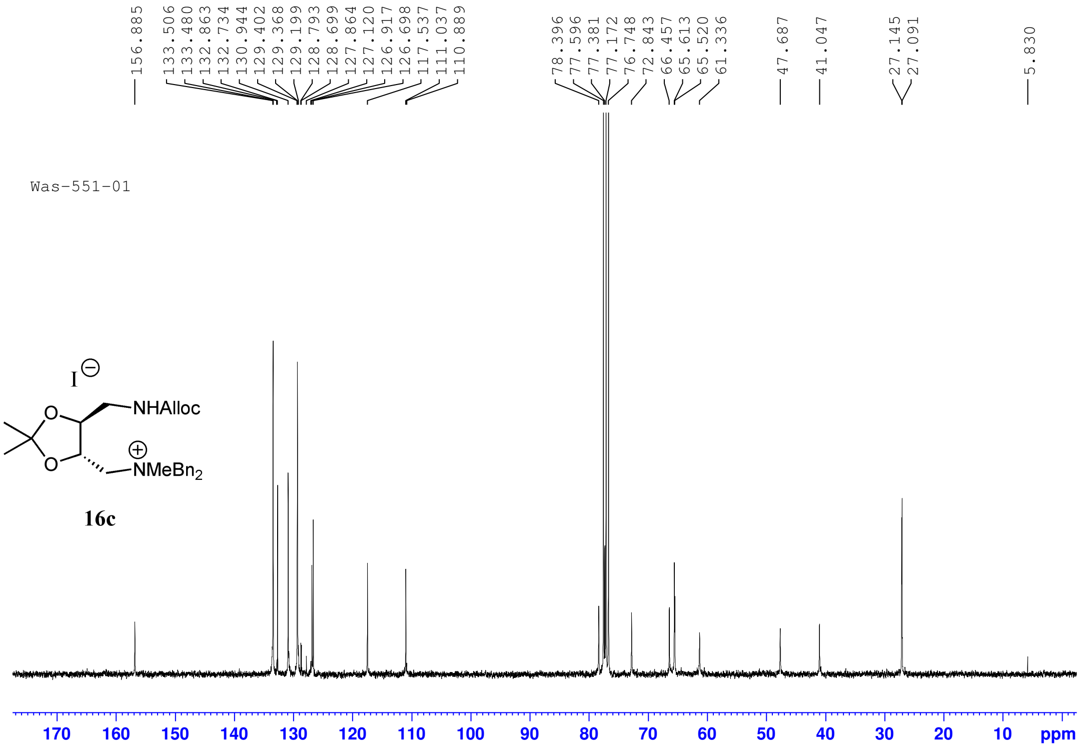
156.885
133.506 133.480 132.863 132.734 130.944 129.402 129.368 129.199 128.793 128.699 127.864 127.120 126.917 126.698 117.537 111.037 110.889
78.396 77.596 77.381 77.172 76.748 72.843 66.457 65.613 65.520 61.336
47.687
41.047
27.145 27.091
5.830
Was-551-01
I
NHAlloc
NMeBn2
16c
170 160 150 140 130 120 110 100 90 80 70 60 50 40 30 20 10 ppm

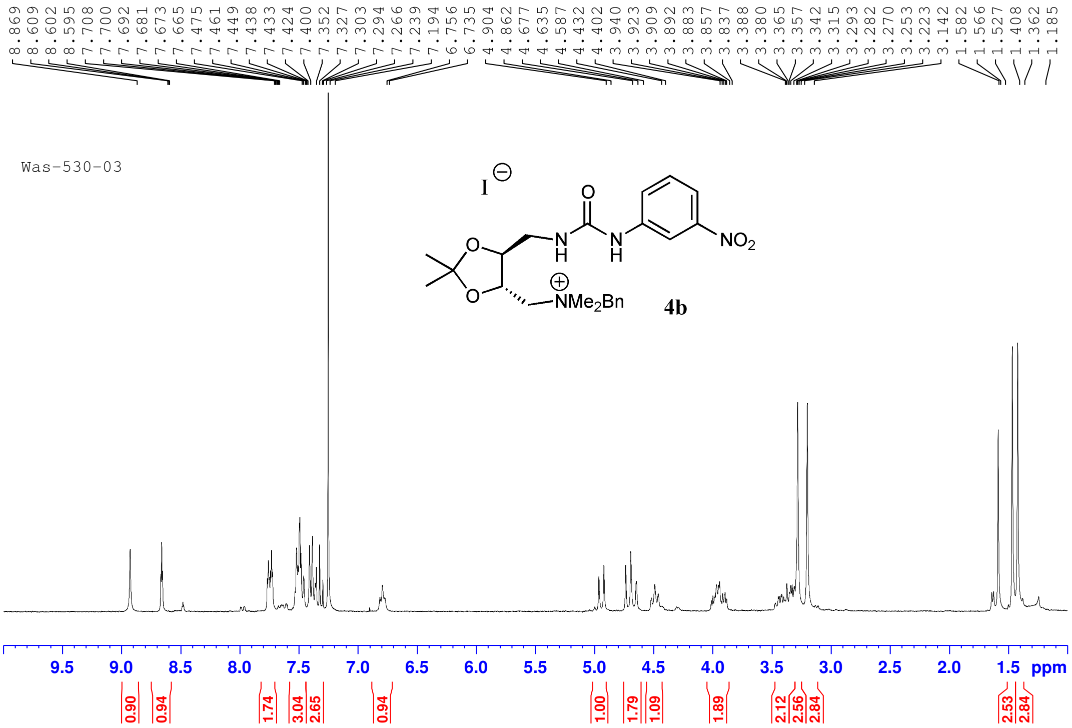
8.869 8.609 8.602 8.595 7.708 7.700 7.692 7.681 7.673 7.665 7.475 7.461 7.449 7.438 7.433 7.424 7.400 7.352 7.327 7.303 7.294 7.266 7.239 7.194 6.756 6.735 4.904 4.862 4.677 4.635 4.587 4.432 4.402 3.940 3.923 3.909 3.892 3.883 3.857 3.837 3.388 3.380 3.365 3.357 3.342 3.315 3.293 3.282 3.270 3.253 3.223 3.142 1.582 1.566 1.527 1.408 1.362 1.185
Was-530-03
I
O
N
H
N
H
NO2
O
O
NMe2Bn
4b
9.5 9.0 8.5 8.0 7.5 7.0 6.5 6.0 5.5 5.0 4.5 4.0 3.5 3.0 2.5 2.0 1.5 ppm
0.90 0.94 1.74 3.04 2.65 0.94 1.00 1.79 1.09 1.89 2.12 2.56 2.84 2.53 2.84

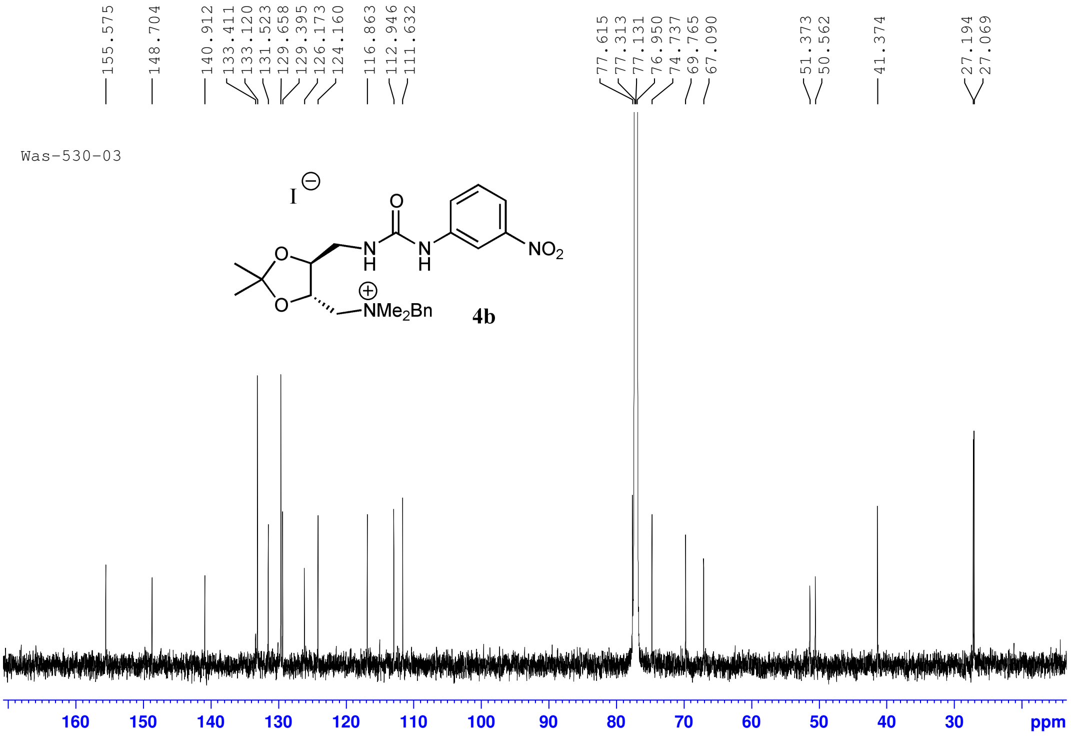
155.575 148.704 140.912 133.411 133.120 131.523 129.658 129.395 126.173 124.160 116.863 112.946 111.632 77.615 77.313 77.131 76.950 74.737 69.765 67.090 51.373 50.562 41.374 27.194 27.069
Was-530-03
I
O
N
H
N
H
NO2
O
O
NMe2Bn
4b
160 150 140 130 120 110 100 90 80 70 60 50 40 30 ppm

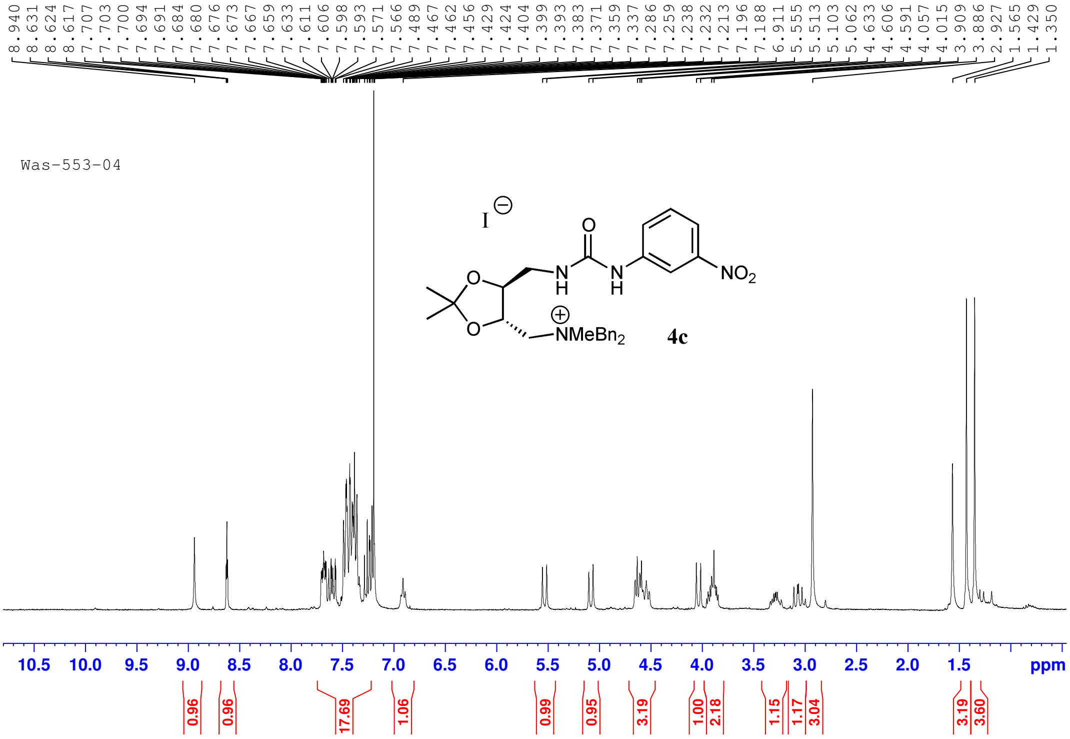
Was-553-04
4c
ppm

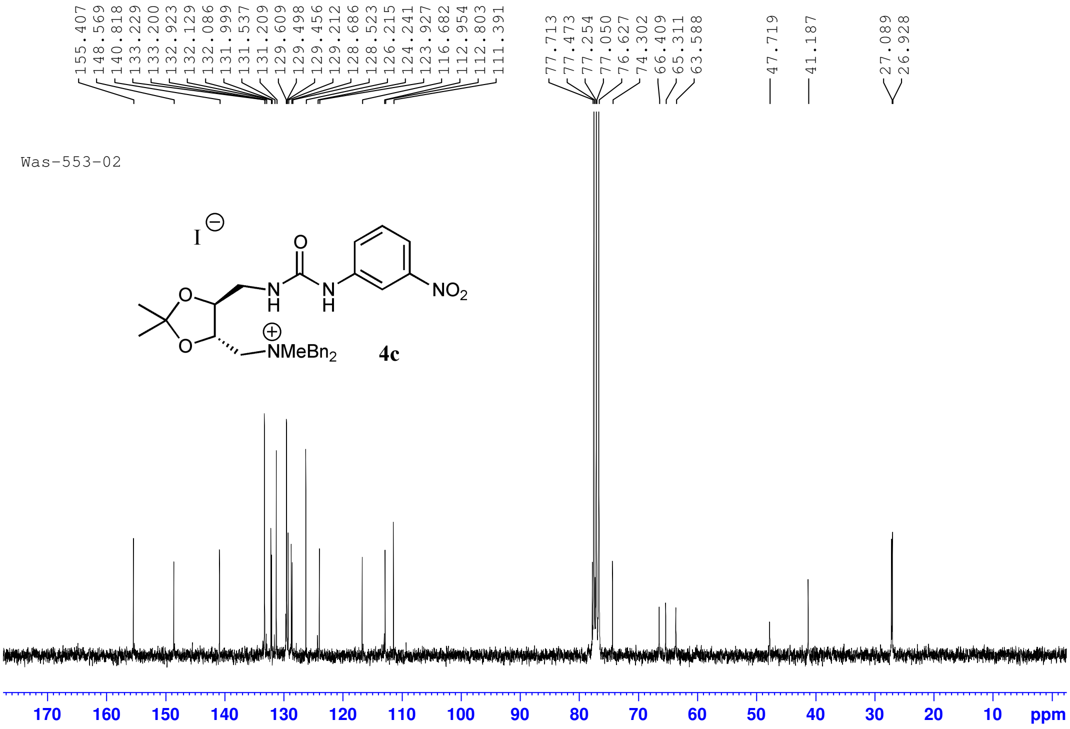
Was-553-02
4c
ppm

5. Copies of NMR Spectra of Selected Known and of the New Fluorination Products:

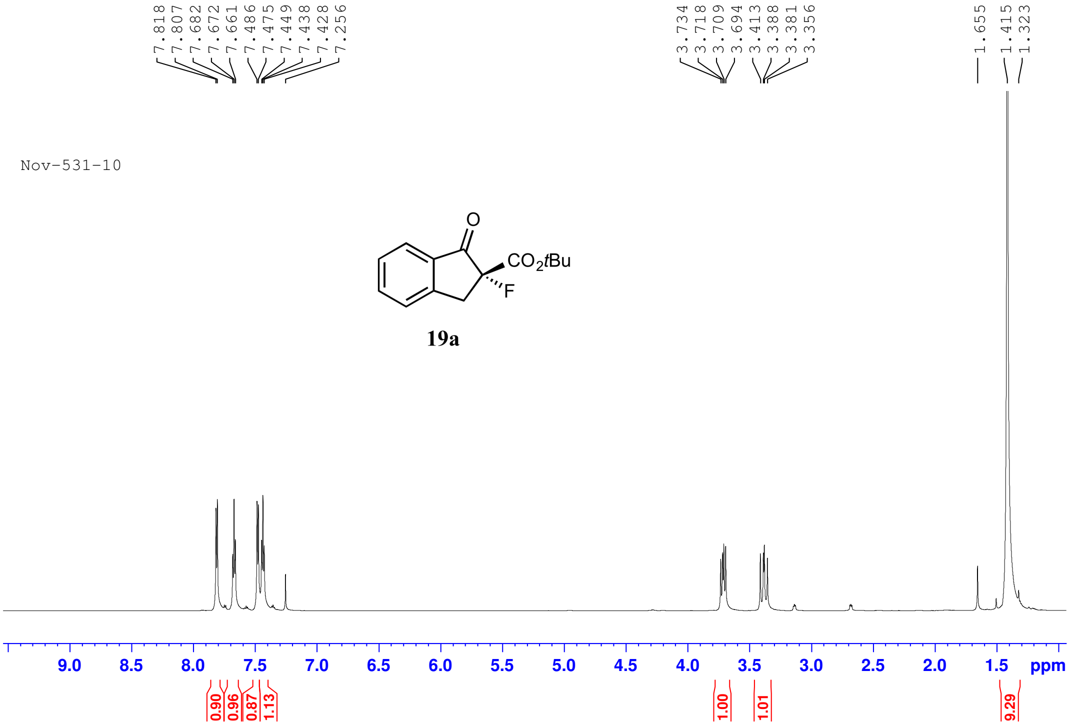

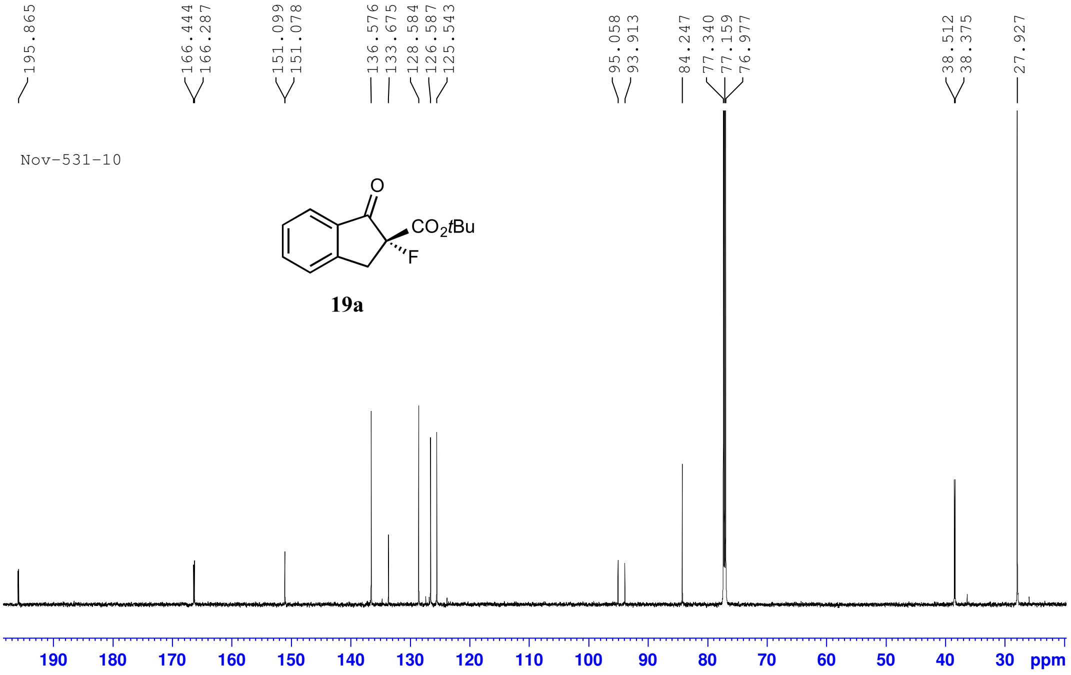

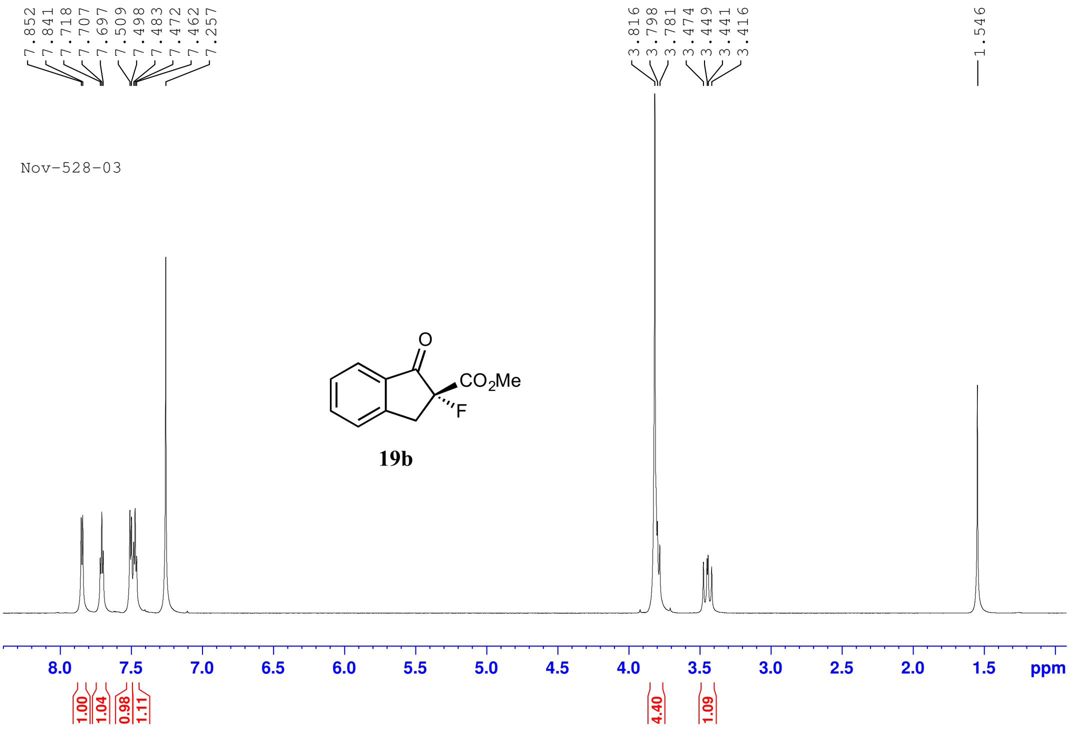
7.852
7.841
7.718
7.707
7.697
7.509
7.498
7.483
7.472
7.462
7.257
3.816
3.798
3.781
3.474
3.449
3.441
3.416
1.546
Nov-528-03
O
CO_2Me
F
19b
8.0 7.5 7.0 6.5 6.0 5.5 5.0 4.5 4.0 3.5 3.0 2.5 2.0 1.5 ppm
1.00
1.04
0.98
1.11
4.40
1.09

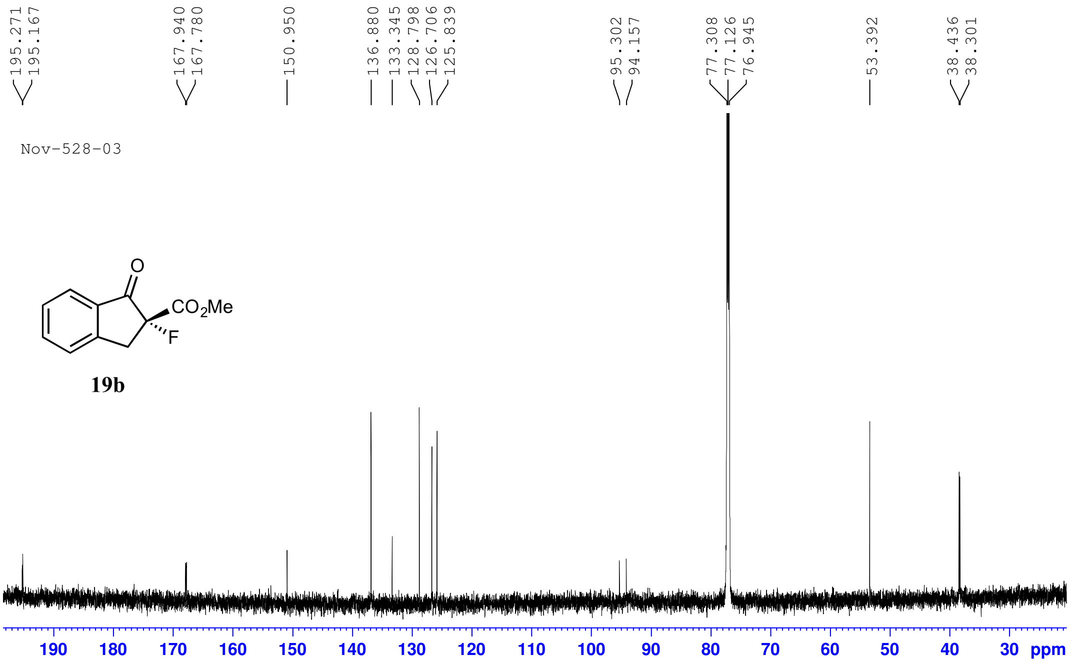
195.271
195.167
167.940
167.780
150.950
136.880
133.345
128.798
126.706
125.839
95.302
94.157
77.308
77.126
76.945
53.392
38.436
38.301
Nov-528-03
O
CO_2Me
F
19b
190 180 170 160 150 140 130 120 110 100 90 80 70 60 50 40 30 ppm

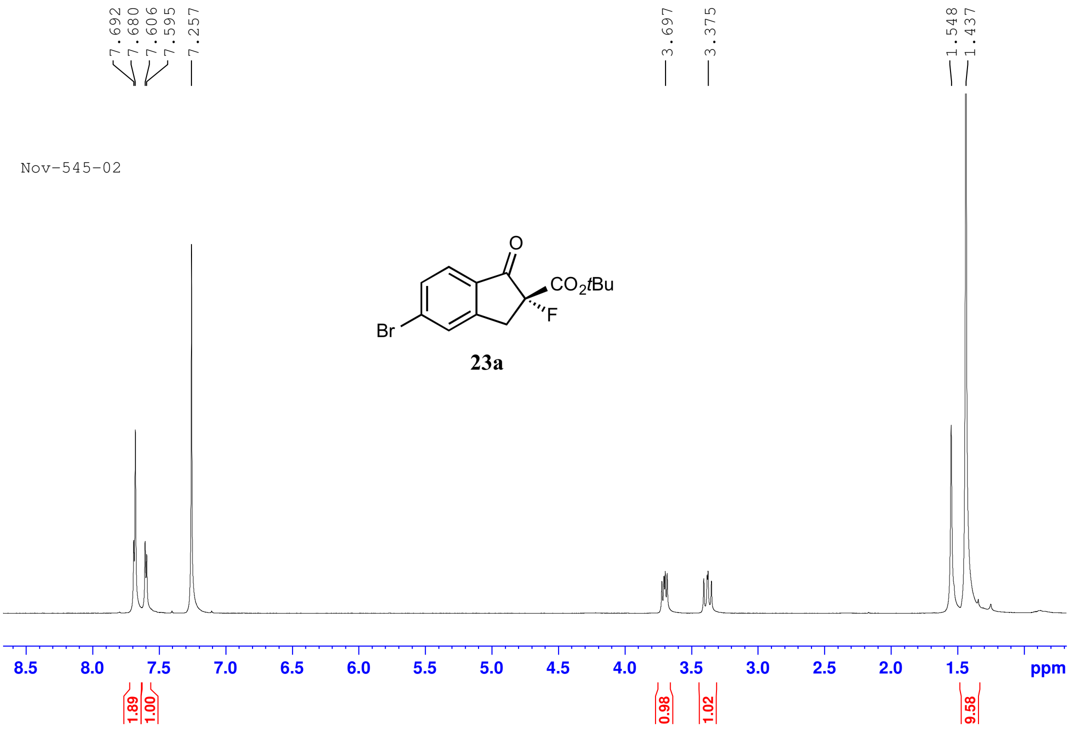
7.692
7.680
7.606
7.595
7.257
3.697
3.375
1.548
1.437
Nov-545-02
O
CO2tBu
F
Br
23a
8.5 8.0 7.5 7.0 6.5 6.0 5.5 5.0 4.5 4.0 3.5 3.0 2.5 2.0 1.5 ppm
1.89
1.00
0.98
1.02
9.58

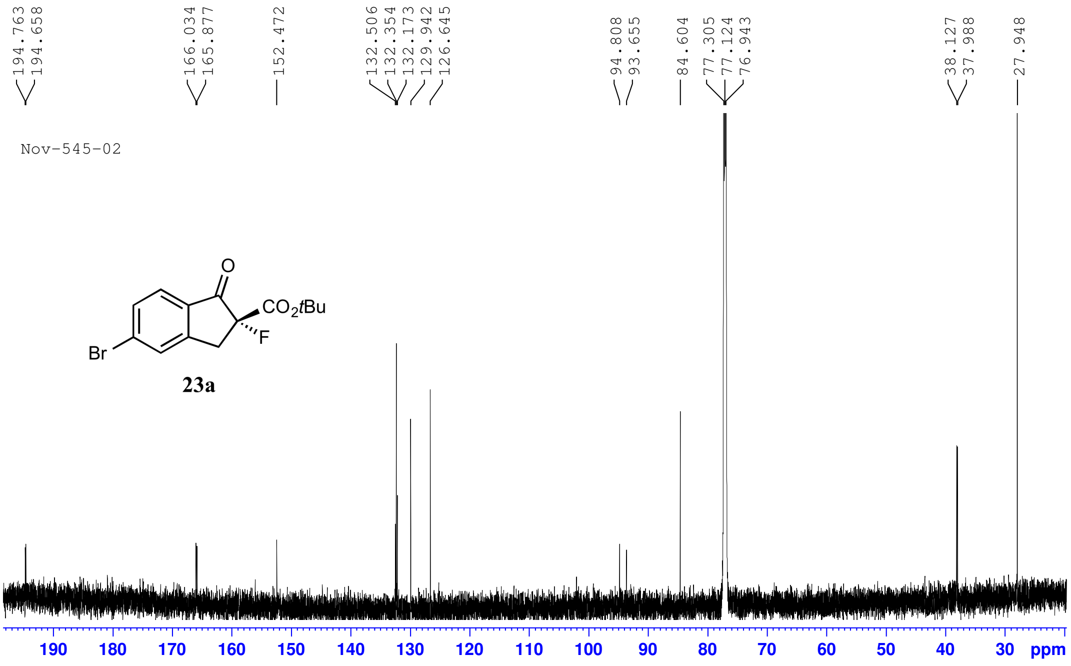
194.763
194.658
166.034
165.877
152.472
132.506
132.354
132.173
129.942
126.645
94.808
93.655
84.604
77.305
77.124
76.943
38.127
37.988
27.948
Nov-545-02
O
CO2tBu
F
Br
23a
190 180 170 160 150 140 130 120 110 100 90 80 70 60 50 40 30 ppm

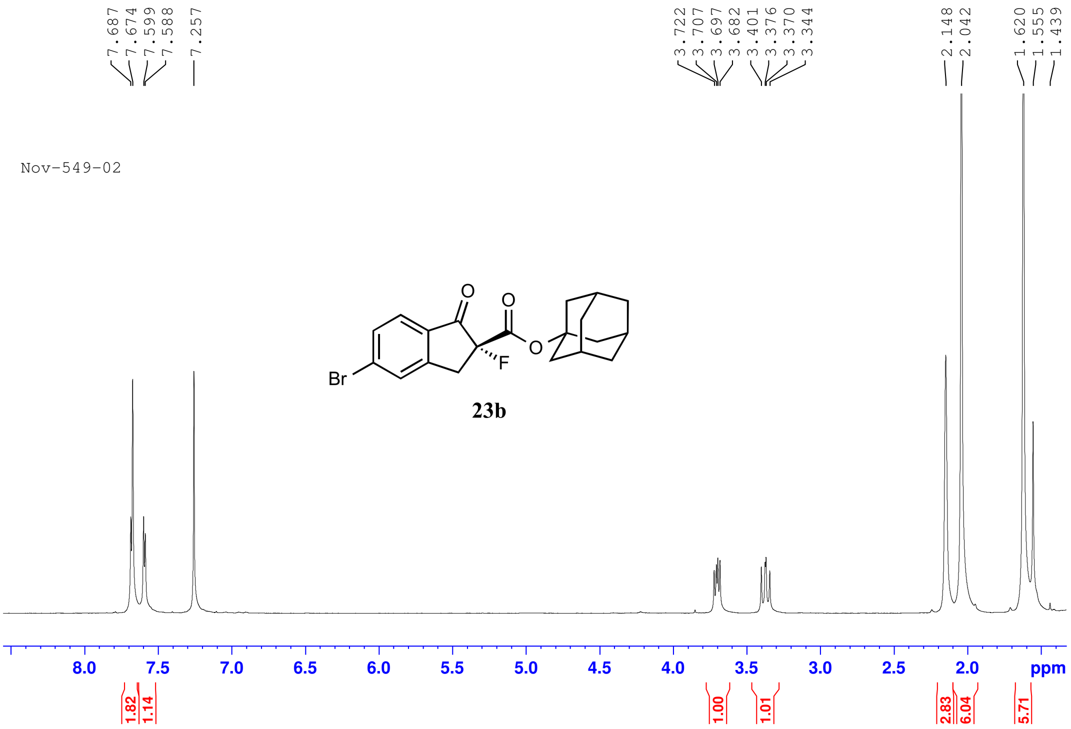
7.687
7.674
7.599
7.588
7.257
3.722
3.707
3.697
3.682
3.401
3.376
3.370
3.344
2.148
2.042
1.620
1.555
1.439
Nov-549-02
O
O
O
F
Br
23b
8.0 7.5 7.0 6.5 6.0 5.5 5.0 4.5 4.0 3.5 3.0 2.5 2.0 ppm
1.82
1.14
1.00
1.01
2.83
6.04
5.71

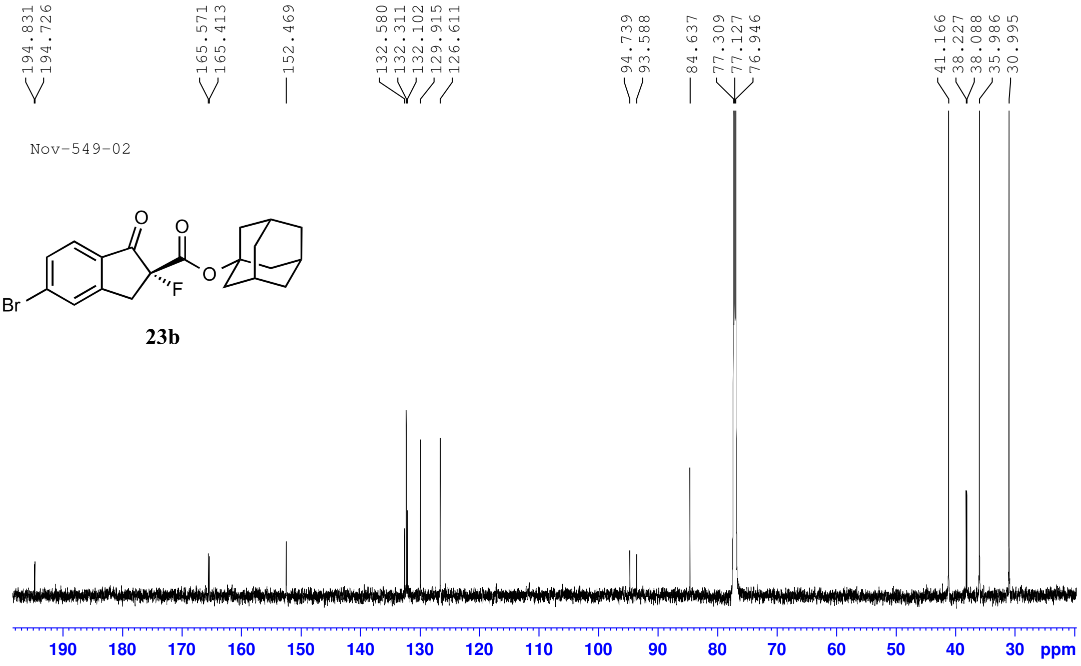
194.831
194.726
165.571
165.413
152.469
132.580
132.311
132.102
129.915
126.611
94.739
93.588
84.637
77.309
77.127
76.946
41.166
38.227
38.088
35.986
30.995
Nov-549-02
O
O
O
F
Br
23b
190 180 170 160 150 140 130 120 110 100 90 80 70 60 50 40 30 ppm

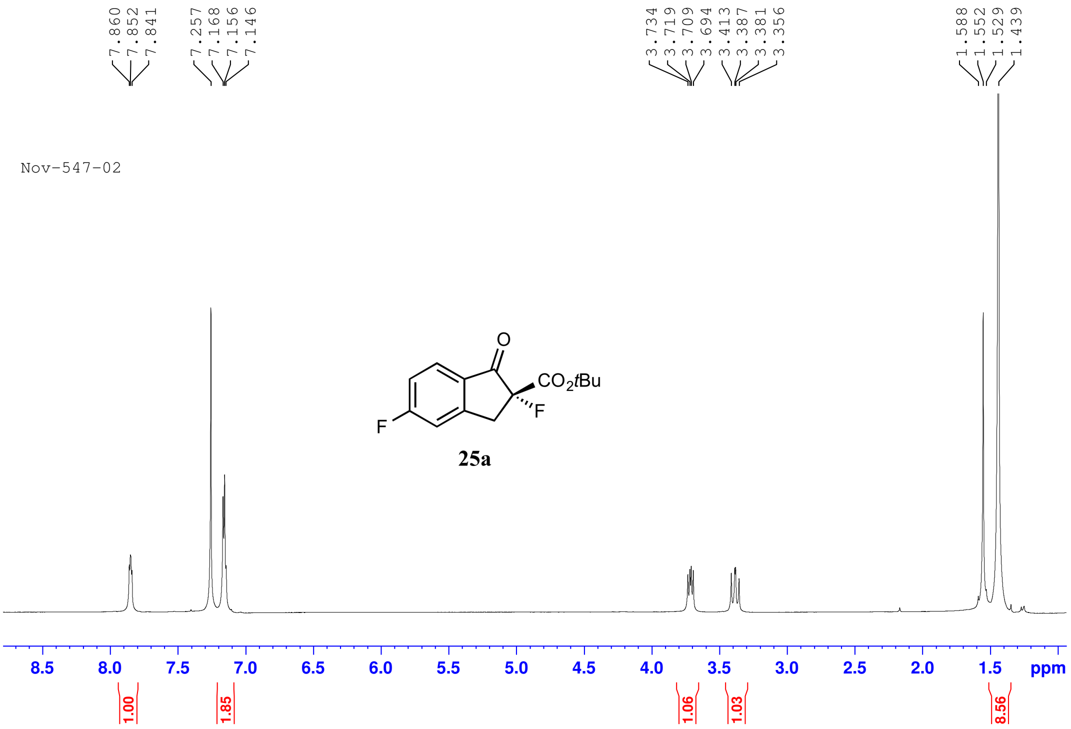

7.860
7.852
7.841
7.257
7.168
7.156
7.146
3.734
3.719
3.709
3.694
3.413
3.387
3.381
3.356
1.588
1.552
1.529
1.439
Nov-547-02
O
CO2tBu
F
F
25a
8.5 8.0 7.5 7.0 6.5 6.0 5.5 5.0 4.5 4.0 3.5 3.0 2.5 2.0 1.5 ppm
1.00
1.85
1.06
1.03
8.56

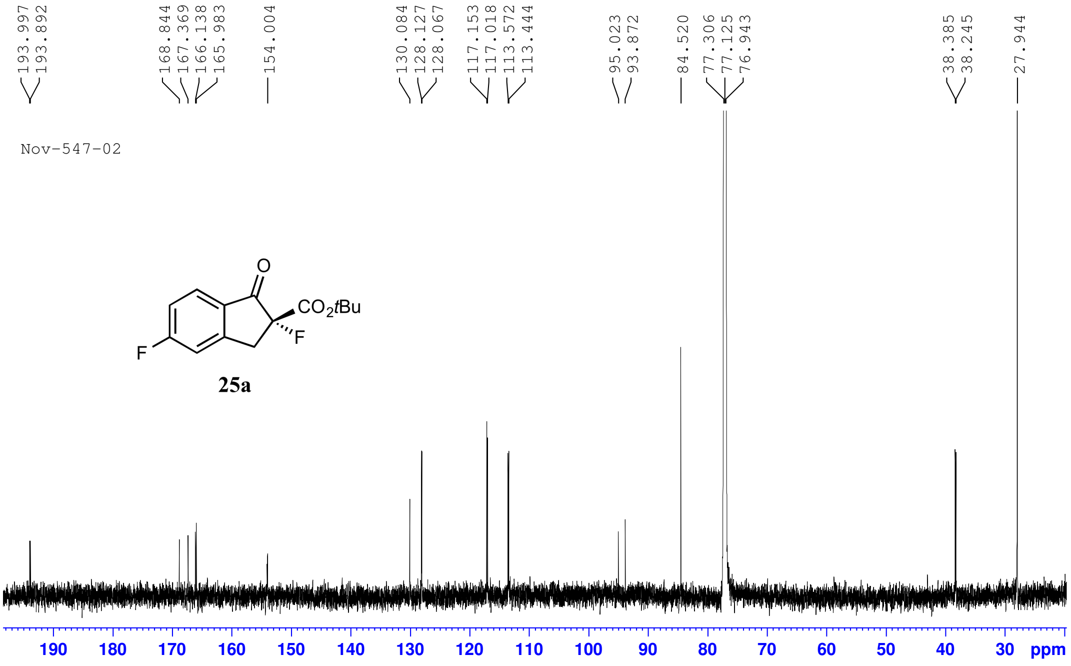

193.997
193.892
168.844
167.369
166.138
165.983
154.004
130.084
128.127
128.067
117.153
117.018
113.572
113.444
95.023
93.872
84.520
77.306
77.125
76.943
38.385
38.245
27.944
Nov-547-02
O
CO2tBu
F
F
25a
190 180 170 160 150 140 130 120 110 100 90 80 70 60 50 40 30 ppm

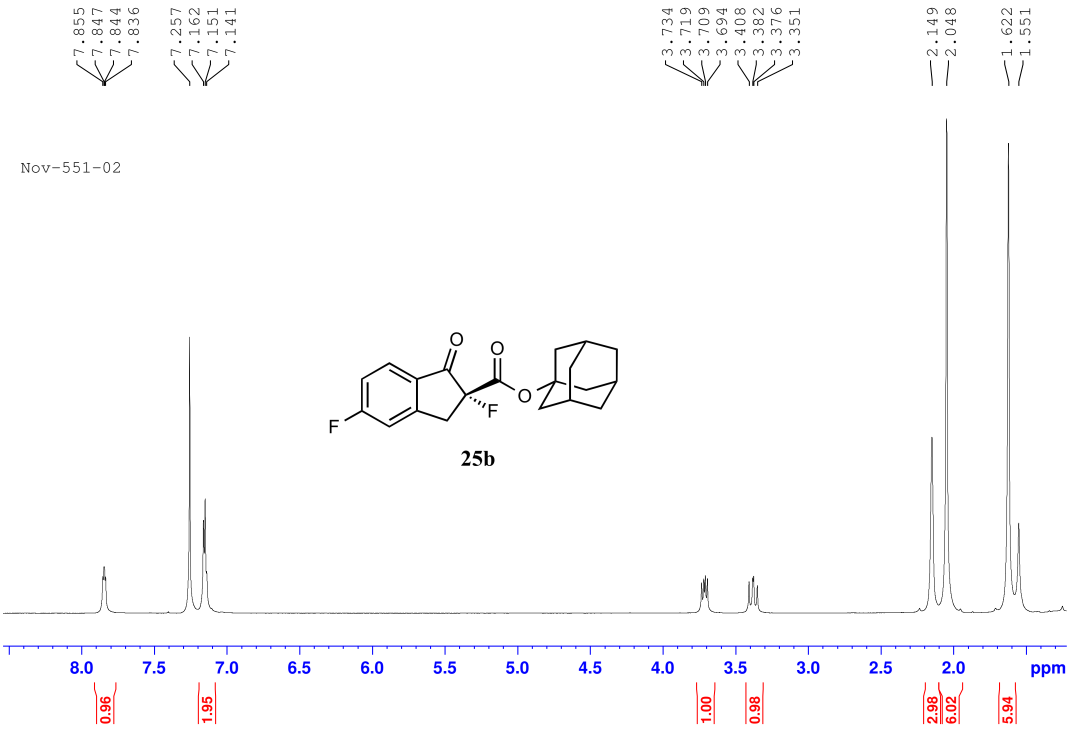
7.855
7.847
7.844
7.836
7.257
7.162
7.151
7.141
3.734
3.719
3.709
3.694
3.408
3.382
3.376
3.351
2.149
2.048
1.622
1.551
Nov-551-02
O
O
O
F
F
25b
8.0 7.5 7.0 6.5 6.0 5.5 5.0 4.5 4.0 3.5 3.0 2.5 2.0 ppm
0.96
1.95
1.00
0.98
2.98
6.02
5.94

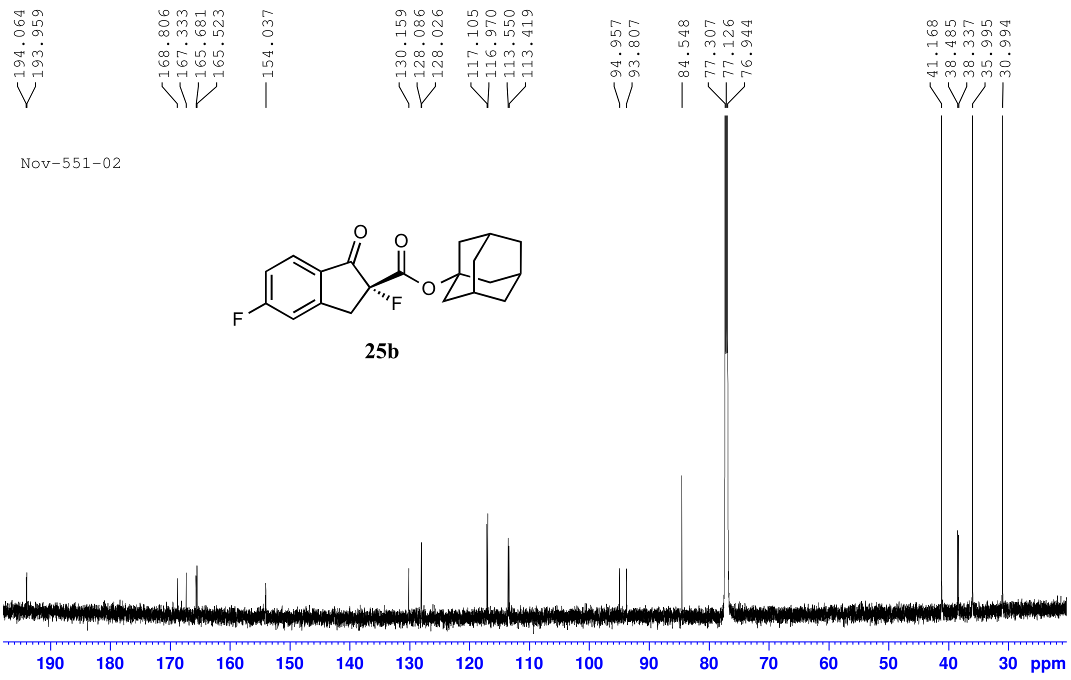
194.064
193.959
168.806
167.333
165.681
165.523
154.037
130.159
128.086
128.026
117.105
116.970
113.550
113.419
94.957
93.807
84.548
77.307
77.126
76.944
41.168
38.485
38.337
35.995
30.994
Nov-551-02
O
O
O
F
F
25b
190 180 170 160 150 140 130 120 110 100 90 80 70 60 50 40 30 ppm

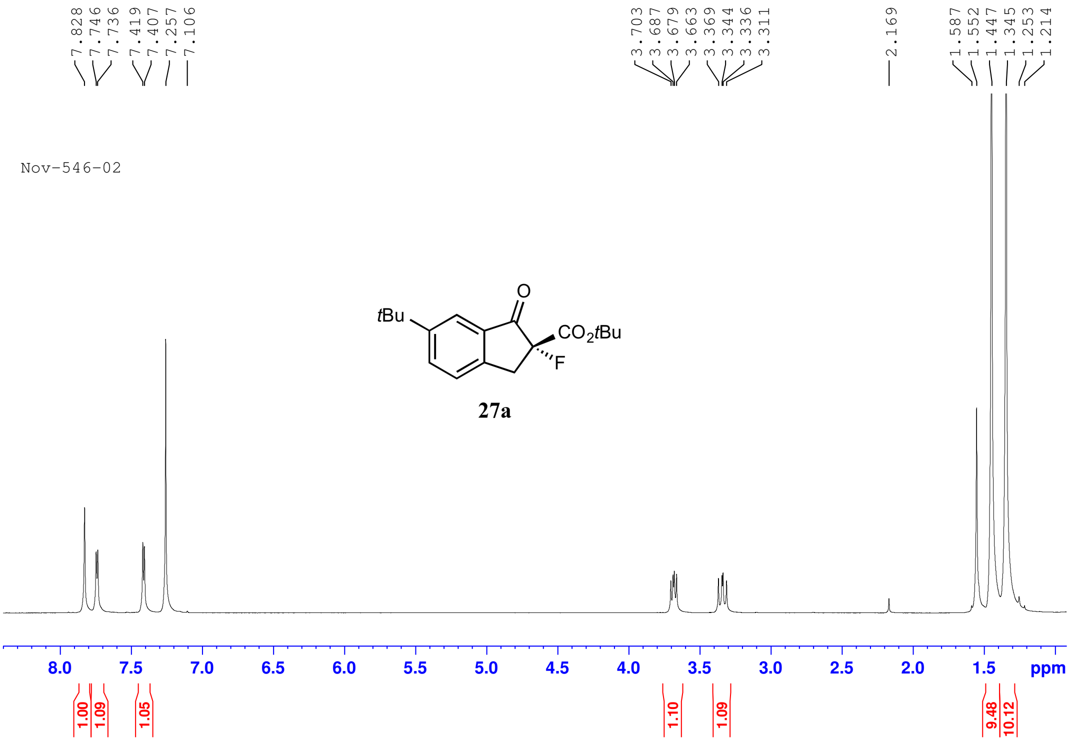
7.828
7.746
7.736
7.419
7.407
7.257
7.106
3.703
3.687
3.679
3.663
3.369
3.344
3.336
3.311
2.169
1.587
1.552
1.447
1.345
1.253
1.214
Nov-546-02
O
tBu
CO_2tBu
F
27a
8.0 7.5 7.0 6.5 6.0 5.5 5.0 4.5 4.0 3.5 3.0 2.5 2.0 1.5 ppm
1.00
1.09
1.05
1.10
1.09
9.48
10.12

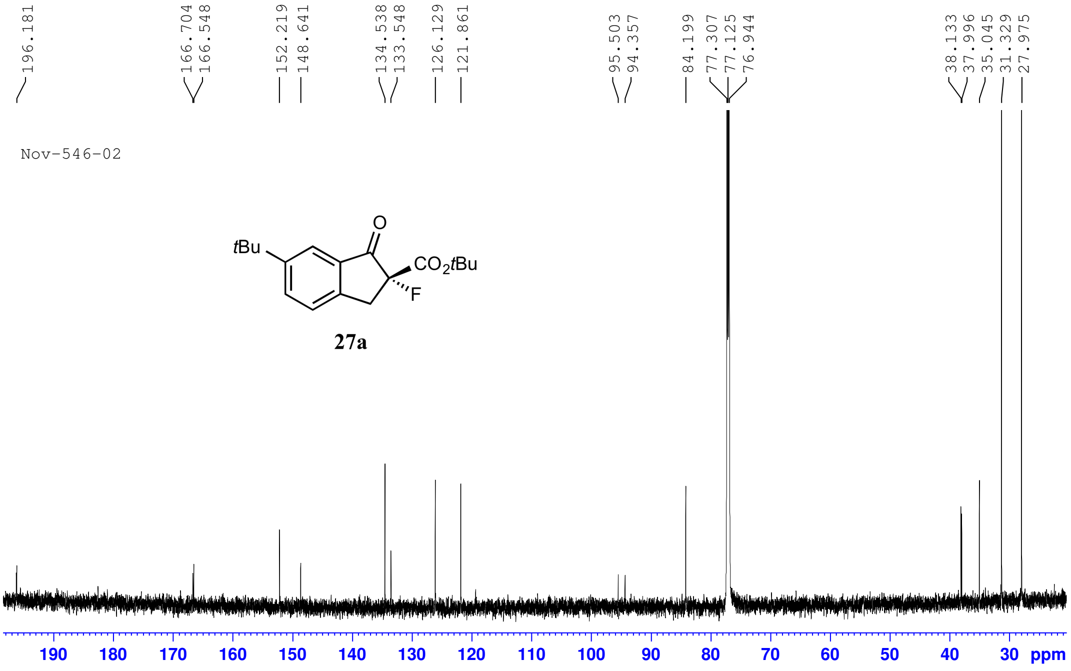
196.181
166.704
166.548
152.219
148.641
134.538
133.548
126.129
121.861
95.503
94.357
84.199
77.307
77.125
76.944
38.133
37.996
35.045
31.329
27.975
Nov-546-02
O
tBu
CO_2tBu
F
27a
190 180 170 160 150 140 130 120 110 100 90 80 70 60 50 40 30 ppm

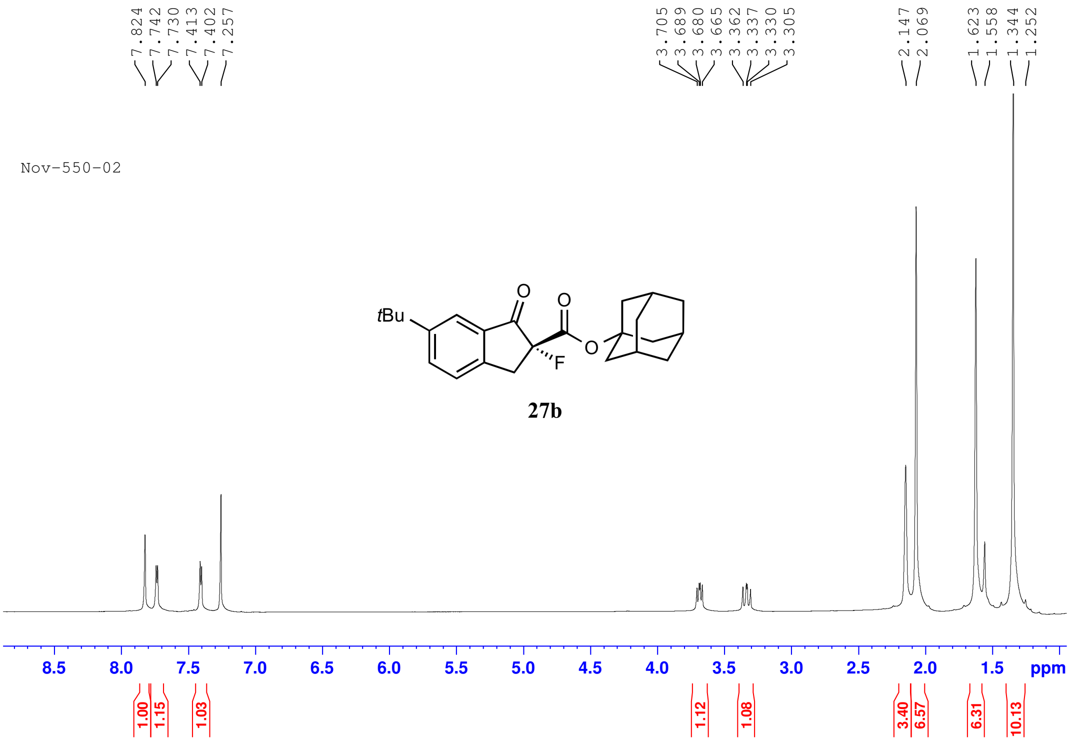
7.824
7.742
7.730
7.413
7.402
7.257
3.705
3.689
3.680
3.665
3.362
3.337
3.330
3.305
2.147
2.069
1.623
1.558
1.344
1.252
Nov-550-02
tBu
O
O
O
F
27b
8.5 8.0 7.5 7.0 6.5 6.0 5.5 5.0 4.5 4.0 3.5 3.0 2.5 2.0 1.5 ppm
1.00
1.15
1.03
1.12
1.08
3.40
6.57
6.31
10.13

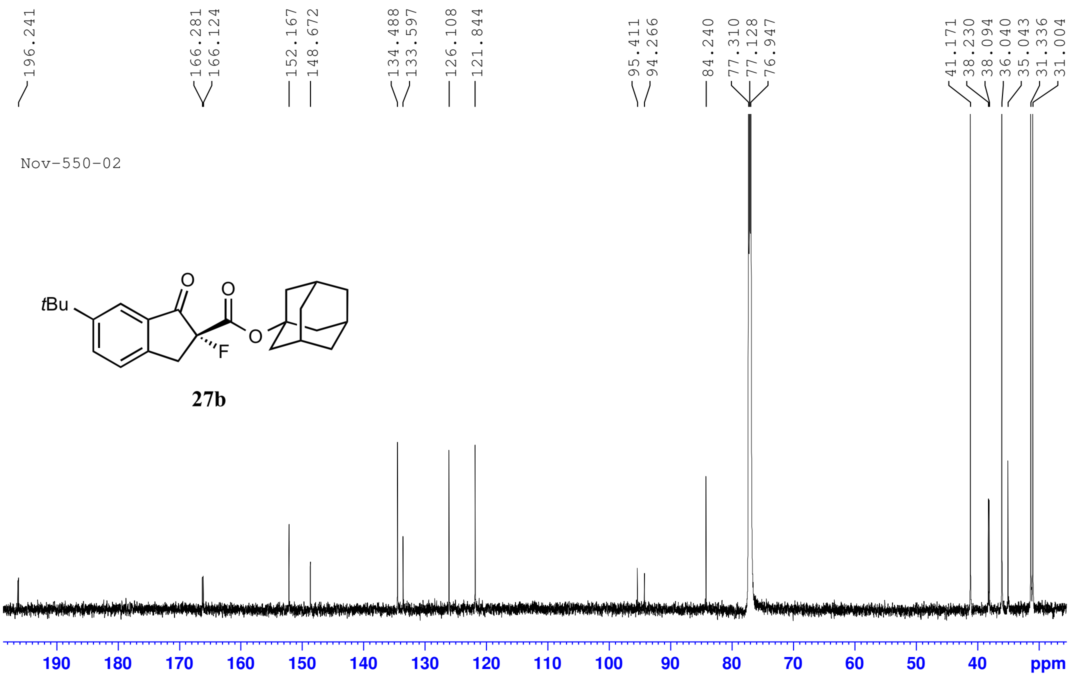
196.241
166.281
166.124
152.167
148.672
134.488
133.597
126.108
121.844
95.411
94.266
84.240
77.310
77.128
76.947
41.171
38.230
38.094
36.040
35.043
31.336
31.004
Nov-550-02
tBu
O
O
O
F
27b
190 180 170 160 150 140 130 120 110 100 90 80 70 60 50 40 ppm

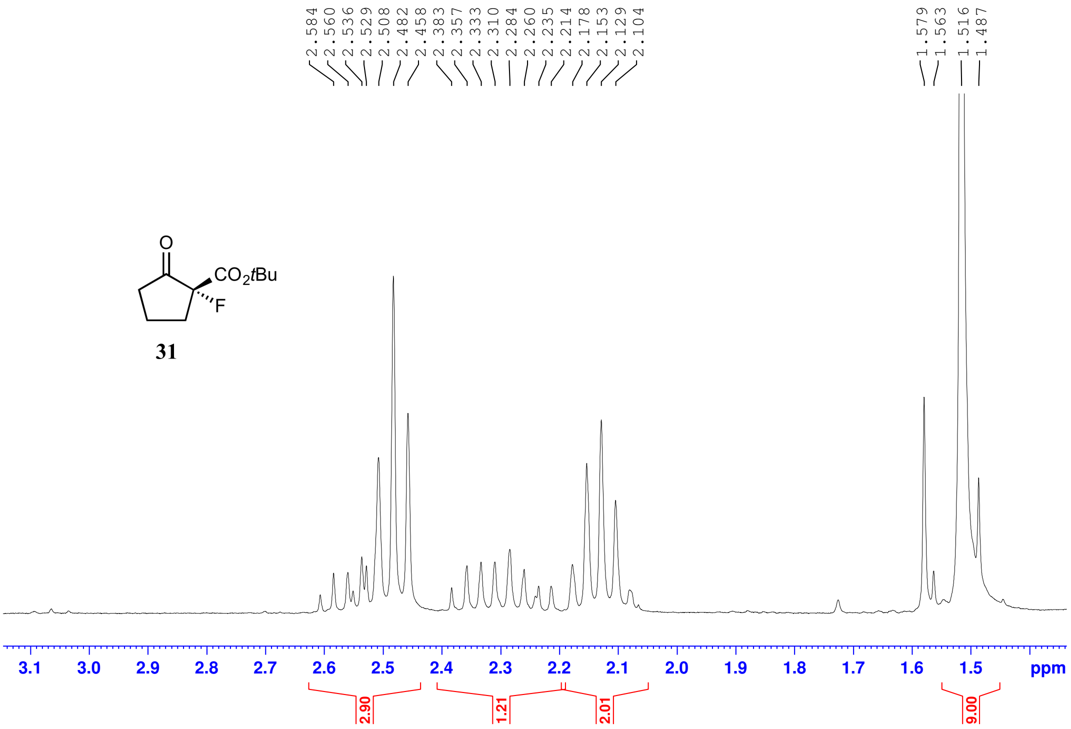
2.584
2.560
2.536
2.529
2.508
2.482
2.458
2.383
2.357
2.333
2.310
2.284
2.260
2.235
2.214
2.178
2.153
2.129
2.104
1.579
1.563
1.516
1.487
O
CO2tBu
F
31
3.1 3.0 2.9 2.8 2.7 2.6 2.5 2.4 2.3 2.2 2.1 2.0 1.9 1.8 1.7 1.6 1.5 ppm
2.90
1.21
2.01
9.00

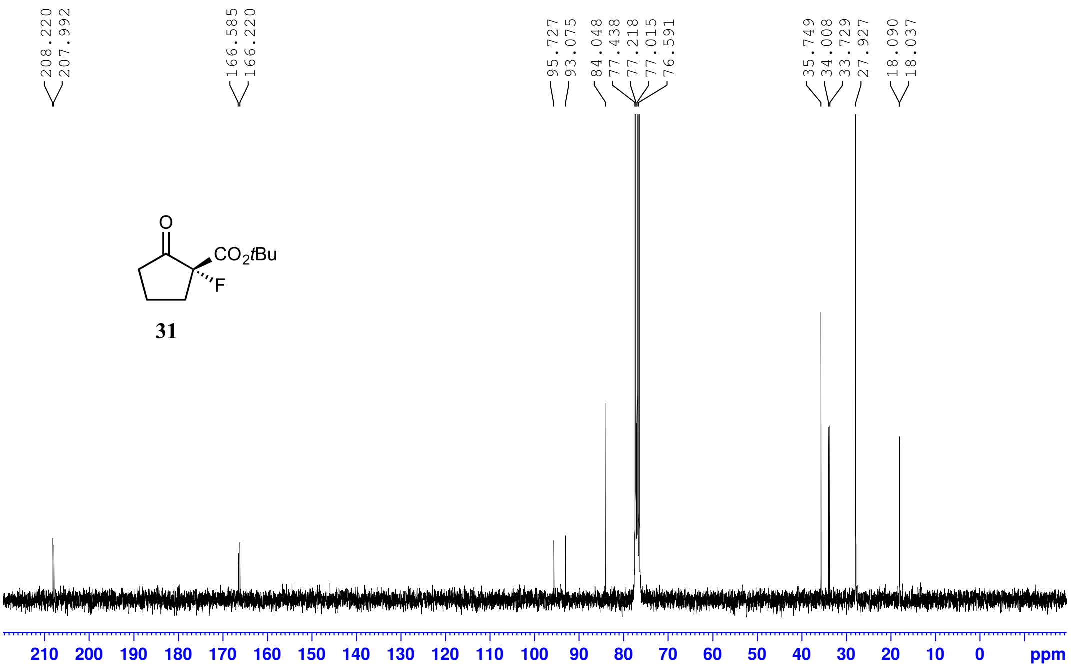
208.220
207.992
166.585
166.220
95.727
93.075
84.048
77.438
77.218
77.015
76.591
35.749
34.008
33.729
27.927
18.090
18.037
O
CO2tBu
F
31
210 200 190 180 170 160 150 140 130 120 110 100 90 80 70 60 50 40 30 20 10 0 ppm

6. HPLC-Chromatograms (Chiral Stationary Phase):

Operator:Admin Timebase:Summit_1 Sequence:WAS_20130728_NOV_ADH Page 1-1
21.8.2013 8:22 PM

3 Nov-531

Sample Name:	**Nov-531**	*Injection Volume:*	**10,0**
Vial Number:	**RA1**	*Channel:*	**UV_VIS_2**
Sample Type:	**unknown**	*Wavelength:*	**n.a.**
Control Program:	**AD_H_60Min_200_1_flow075**	*Bandwidth:*	**n.a.**
Quantif. Method:	**AD_H**	*Dilution Factor:*	**1,0000**
Recording Time:	**28.7.2013 21:05**	*Sample Weight:*	**1,0000**
Run Time (min):	**60,00**	*Sample Amount:*	**1,0000**

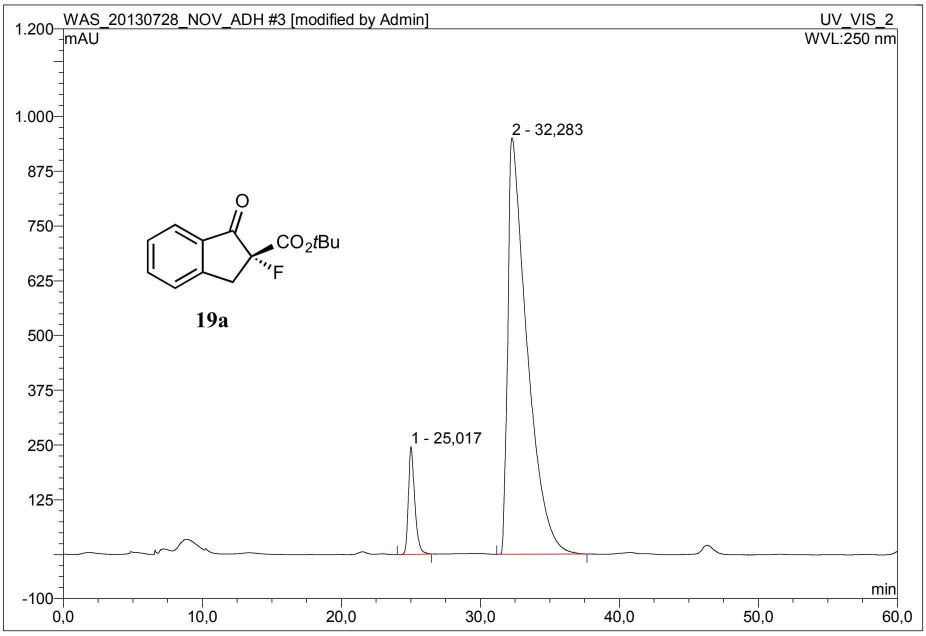

No.	Ret.Time min	Peak Name	Height mAU	Area mAU*min	Rel.Area %	Amount	Type
1	25,02	n.a.	245,853	129,777	7,82	n.a.	BMB*
2	32,28	n.a.	950,178	1529,542	92,18	n.a.	BMB*
**Total:**			1196,031	1659,319	100,00	0,000	

2 Nov-528

Sample Name:	**Nov-528**	*Injection Volume:*	**10,0**
Vial Number:	**RA3**	*Channel:*	**UV_VIS_2**
Sample Type:	**unknown**	*Wavelength:*	**n.a.**
Control Program:	**OD_H_90Min_95_5_flow075**	*Bandwidth:*	**n.a.**
Quantif. Method:	**OD_H**	*Temperature/Column:*	**10**
Recording Time:	**30.7.2013 18:52**	*Flow ml/min:*	**0,750**
Run Time (min):	**58,64**	*Sample Amount:*	**1,0000**

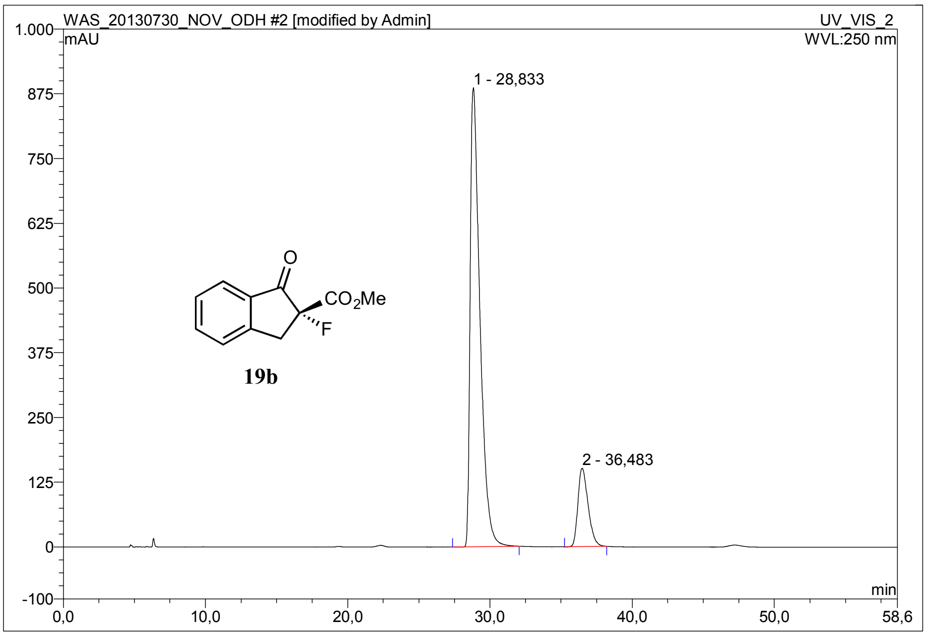

No.	Ret.Time min	Peak Name	Height mAU	Area mAU*min	Rel.Area %	Amount	Type
1	28,83	n.a.	886,701	704,635	84,87	n.a.	BMB*
2	36,48	n.a.	151,402	125,598	15,13	n.a.	BMB*
**Total:**			1038,103	830,232	100,00	0,000	

95 n-Hexan : 5 iso-Prop.

5 Nov-528-Krist1

Sample Name:	**Nov-528-Krist1**	*Injection Volume:*	**10,0**
Vial Number:	**RA6**	*Channel:*	**UV_VIS_2**
Sample Type:	**unknown**	*Wavelength:*	**n.a.**
Control Program:	**OD_H_90Min_95_5_flow075**	*Bandwidth:*	**n.a.**
Quantif. Method:	**OD_H**	*Temperature/Column:*	**10**
Recording Time:	**30.7.2013 21:54**	*Flow ml/min:*	**0,750**
Run Time (min):	**50,03**	*Sample Amount:*	**1,0000**

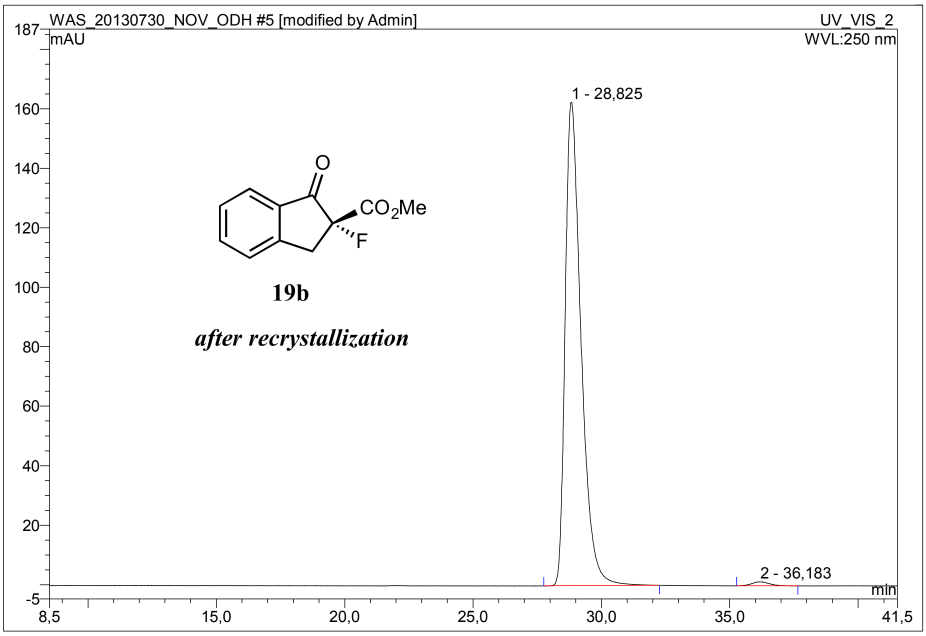

No.	Ret.Time min	Peak Name	Height mAU	Area mAU*min	Rel.Area %	Amount	Type
1	28,83	n.a.	162,579	116,457	99,11	n.a.	BMB*
2	36,18	n.a.	1,363	1,043	0,89	n.a.	BMB*
**Total:**			163,942	117,500	100,00	0,000	

95 n-Hexan : 5 iso-Prop.

3 Nov-533 verdünnt (90/10)

Sample Name:	**Nov-533 verdünnt (90/10)**	*Injection Volume:*	**10,0**
Vial Number:	**RC2**	*Channel:*	**UV_VIS_1**
Sample Type:	**unknown**	*Wavelength:*	**n.a.**
Control Program:	**AD_H_30Min_200_1_flow075**	*Bandwidth:*	**n.a.**
Quantif. Method:	**AD_H**	*Dilution Factor:*	**1,0000**
Recording Time:	**2.8.2013 14:35**	*Sample Weight:*	**1,0000**
Run Time (min):	**30,00**	*Sample Amount:*	**1,0000**

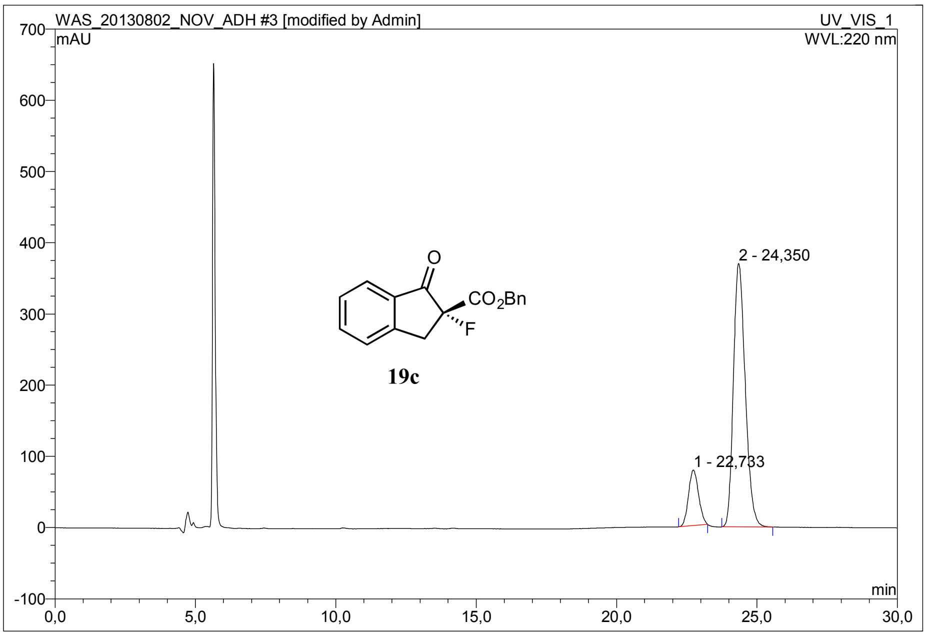

No.	Ret.Time min	Peak Name	Height mAU	Area mAU*min	Rel.Area %	Amount	Type
1	22,73	n.a.	77,795	32,222	15,13	n.a.	BMB*
2	24,35	n.a.	369,983	180,743	84,87	n.a.	BMB*
**Total:**			447,778	212,964	100,00	0,000	

2 Nov-552 gesäult (90:10)

Sample Name:	**Nov-552 gesäult (90:10)**	*Injection Volume:*	**10,0**
Vial Number:	**RC2**	*Channel:*	**UV_VIS_2**
Sample Type:	**unknown**	*Wavelength:*	**n.a.**
Control Program:	**AD_H_30Min_200_1_flow075**	*Bandwidth:*	**n.a.**
Quantif. Method:	**AD_H**	*Dilution Factor:*	**1,0000**
Recording Time:	**20.8.2013 16:07**	*Sample Weight:*	**1,0000**
Run Time (min):	**30,00**	*Sample Amount:*	**1,0000**

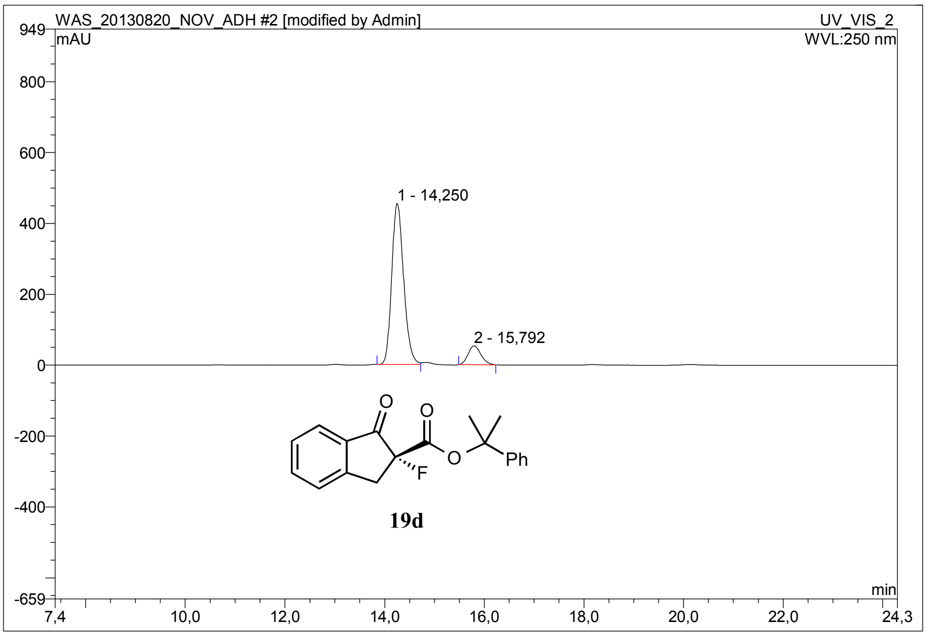

19d

No.	Ret.Time min	Peak Name	Height mAU	Area mAU*min	Rel.Area %	Amount	Type
1	14,25	n.a.	454,405	125,980	88,81	n.a.	BM *
2	15,79	n.a.	53,275	15,880	11,19	n.a.	BMB*
**Total:**			507,679	141,861	100,00	0,000	

3 NOV-532-16-26

Sample Name:	**NOV-532-16-26**	*Injection Volume:*	**10,0**
Vial Number:	**RA1**	*Channel:*	**UV_VIS_2**
Sample Type:	**unknown**	*Wavelength:*	**n.a.**
Control Program:	**OD_R_120Min_55_45_flow08**	*Bandwidth:*	**n.a.**
Quantif. Method:	**OD_R**	*Temperature/Column:*	**10**
Recording Time:	**7.8.2013 13:12**	*Flow ml/min:*	**0,800**
Run Time (min):	**120,00**	*Sample Amount:*	**1,0000**

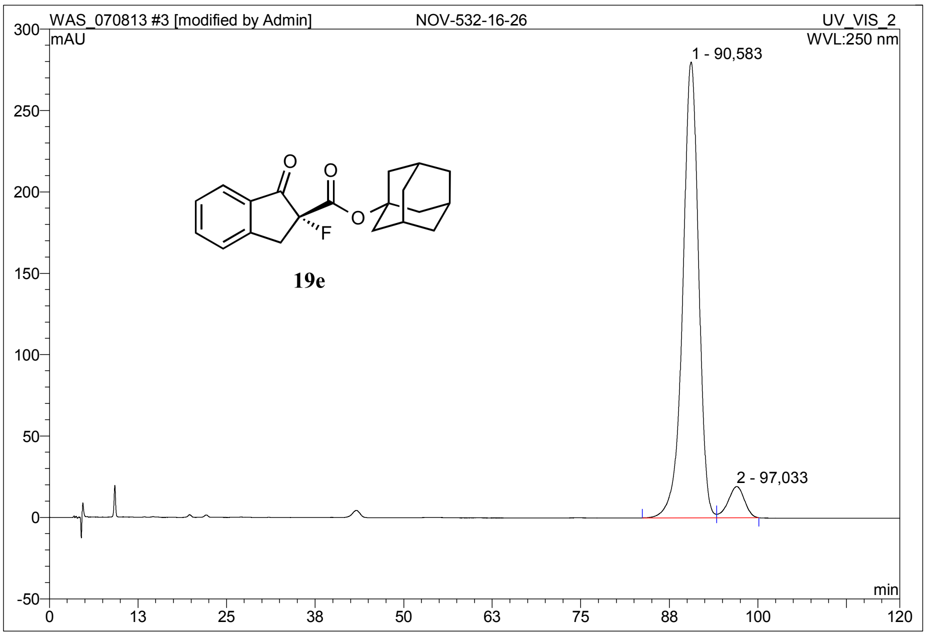

No.	Ret.Time min	Peak Name	Height mAU	Area mAU*min	Rel.Area %	Amount	Type
1	90,58	n.a.	279,976	725,099	93,40	n.a.	BM *
2	97,03	n.a.	19,186	51,208	6,60	n.a.	MB*
**Total:**			299,162	776,307	100,00	0,000	

H2O

1 Nov-545 (95:5)

Sample Name:	**Nov-545 (95:5)**	*Injection Volume:*	**10,0**
Vial Number:	**RB1**	*Channel:*	**UV_VIS_2**
Sample Type:	**unknown**	*Wavelength:*	**n.a.**
Control Program:	**OD_H_90Min_95_5_flow075**	*Bandwidth:*	**n.a.**
Quantif. Method:	**OD_H**	*Temperature/Column:*	**10**
Recording Time:	**18.8.2013 13:21**	*Flow ml/min:*	**0,750**
Run Time (min):	**17,07**	*Sample Amount:*	**1,0000**

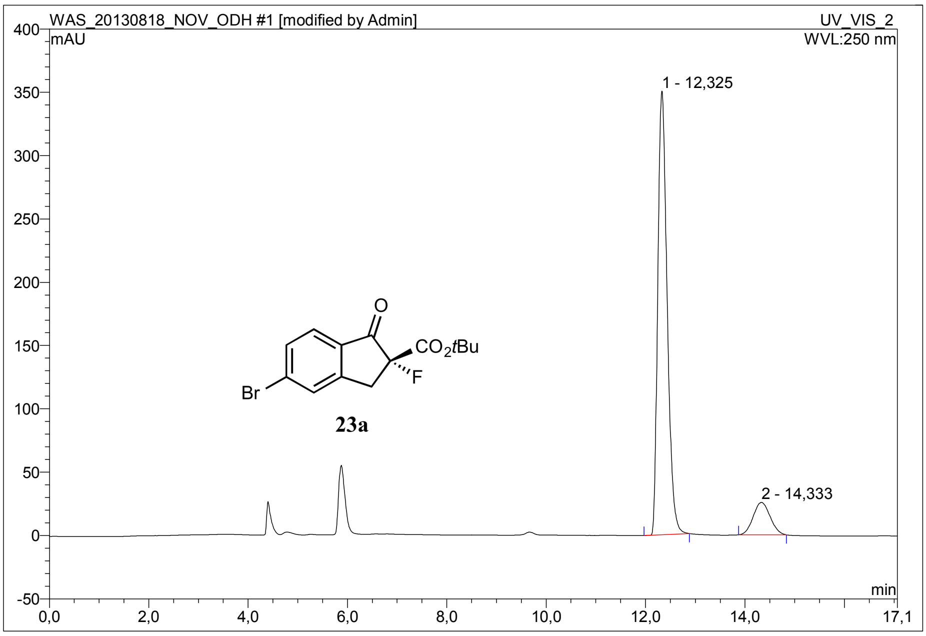

No.	Ret.Time min	Peak Name	Height mAU	Area mAU*min	Rel.Area %	Amount	Type
1	12,33	n.a.	350,358	76,125	88,30	n.a.	BMB*
2	14,33	n.a.	25,539	10,090	11,70	n.a.	BMB*
**Total:**			375,898	86,215	100,00	0,000	

95 n-Hexan : 5 iso-Prop.

4 Nov-549 (90:10)

Sample Name:	**Nov-549 (90:10)**	*Injection Volume:*	**10,0**
Vial Number:	**RC4**	*Channel:*	**UV_VIS_2**
Sample Type:	**unknown**	*Wavelength:*	**n.a.**
Control Program:	**AD_H_90Min_200_1_flow075**	*Bandwidth:*	**n.a.**
Quantif. Method:	**AD_H**	*Dilution Factor:*	**1,0000**
Recording Time:	**20.8.2013 18:13**	*Sample Weight:*	**1,0000**
Run Time (min):	**30,17**	*Sample Amount:*	**1,0000**

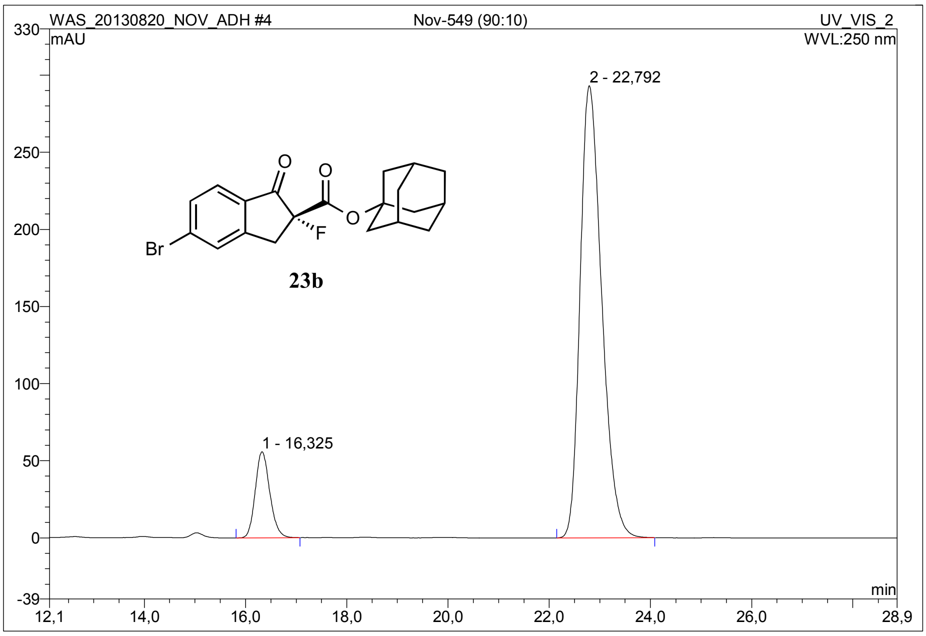

No.	Ret.Time min	Peak Name	Height mAU	Area mAU*min	Rel.Area %	Amount	Type
1	16,33	n.a.	55,840	18,559	11,37	n.a.	BMB
2	22,79	n.a.	293,118	144,638	88,63	n.a.	BMB
**Total:**			348,958	163,197	100,00	0,000	

6 Nov-549 Krist1 (90:10)

Sample Name:	**Nov-549 Krist1 (90:10)**	*Injection Volume:*	**10,0**
Vial Number:	**RC5**	*Channel:*	**UV_VIS_2**
Sample Type:	**unknown**	*Wavelength:*	**n.a.**
Control Program:	**AD_H_30Min_200_1_flow075**	*Bandwidth:*	**n.a.**
Quantif. Method:	**AD_H**	*Dilution Factor:*	**1,0000**
Recording Time:	**20.8.2013 19:39**	*Sample Weight:*	**1,0000**
Run Time (min):	**30,00**	*Sample Amount:*	**1,0000**

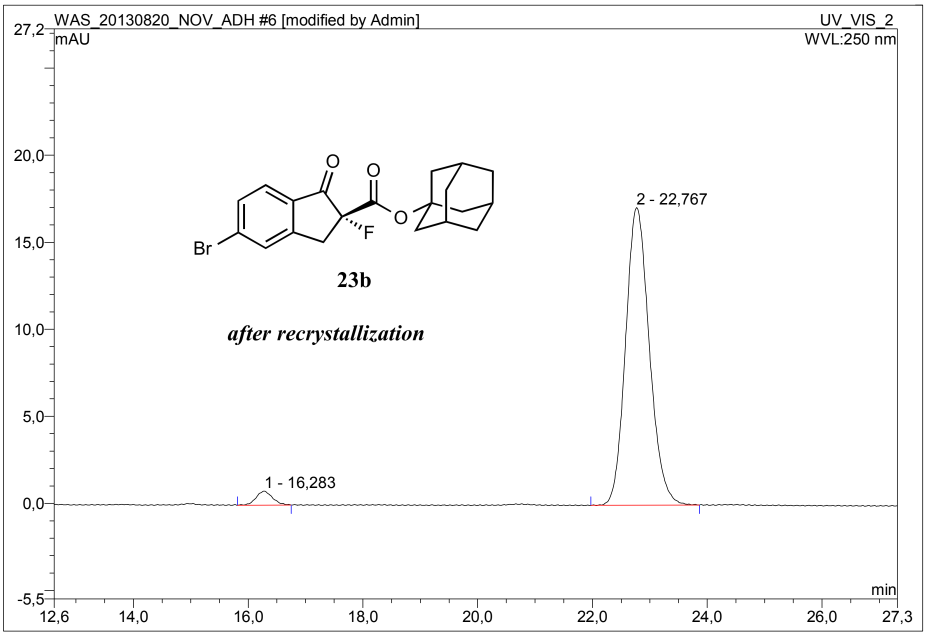

No.	Ret.Time min	Peak Name	Height mAU	Area mAU*min	Rel.Area %	Amount	Type
1	16,28	n.a.	0,809	0,269	3,18	n.a.	BMB*
2	22,77	n.a.	17,095	8,204	96,82	n.a.	BMB*
**Total:**			17,904	8,473	100,00	0,000	

1 Nov-547 (95:5)

Sample Name:	**Nov-547 (95:5)**	*Injection Volume:*	**10,0**
Vial Number:	**RB2**	*Channel:*	**UV_VIS_2**
Sample Type:	**unknown**	*Wavelength:*	**n.a.**
Control Program:	**AD_H_90Min_200_1_flow075**	*Bandwidth:*	**n.a.**
Quantif. Method:	**AD_H**	*Dilution Factor:*	**1,0000**
Recording Time:	**18.8.2013 19:15**	*Sample Weight:*	**1,0000**
Run Time (min):	**16,12**	*Sample Amount:*	**1,0000**

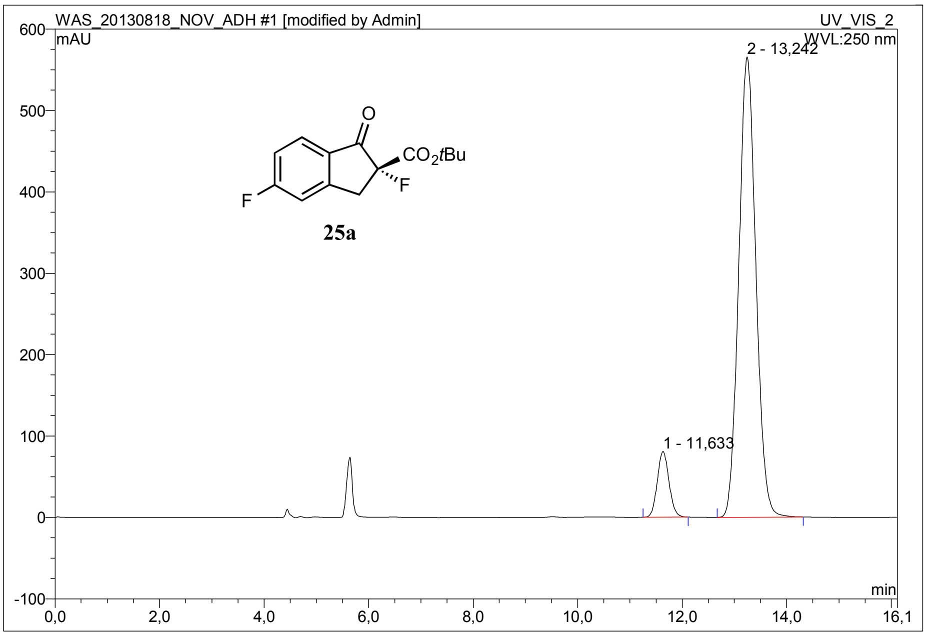

No.	Ret.Time min	Peak Name	Height mAU	Area mAU*min	Rel.Area %	Amount	Type
1	11,63	n.a.	80,834	20,966	8,92	n.a.	BMB*
2	13,24	n.a.	565,423	214,058	91,08	n.a.	BMB*
**Total:**			646,257	235,023	100,00	0,000	

3 Nov-551 (90:10)

Sample Name:	**Nov-551 (90:10)**	*Injection Volume:*	**10,0**
Vial Number:	**RC3**	*Channel:*	**UV_VIS_2**
Sample Type:	**unknown**	*Wavelength:*	**n.a.**
Control Program:	**AD_H_90Min_200_1_flow075**	*Bandwidth:*	**n.a.**
Quantif. Method:	**AD_H**	*Dilution Factor:*	**1,0000**
Recording Time:	**20.8.2013 17:01**	*Sample Weight:*	**1,0000**
Run Time (min):	**30,02**	*Sample Amount:*	**1,0000**

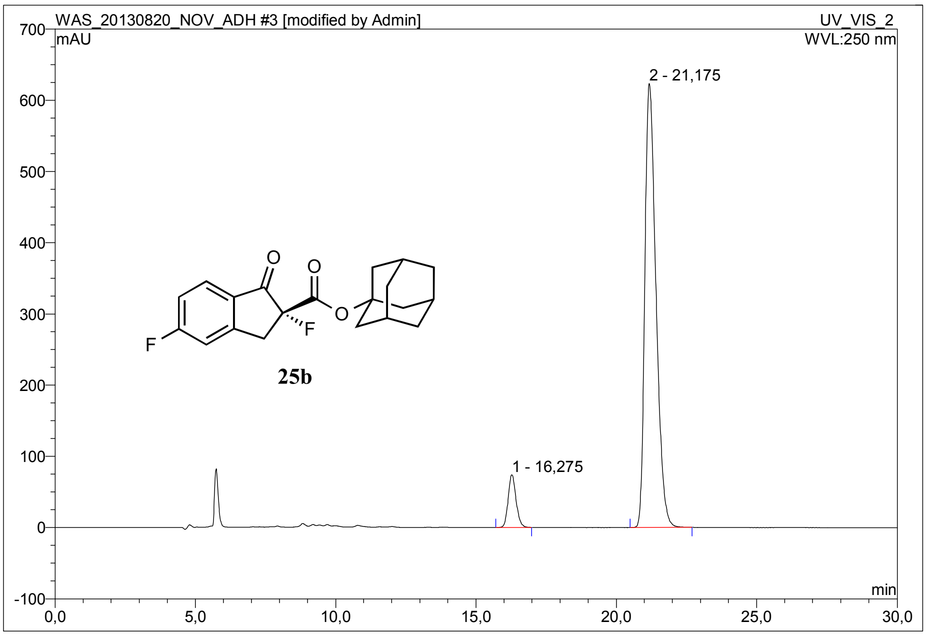

No.	Ret.Time min	Peak Name	Height mAU	Area mAU*min	Rel.Area %	Amount	Type
1	16,28	n.a.	73,770	24,140	7,78	n.a.	BMB
2	21,18	n.a.	623,326	286,155	92,22	n.a.	BMB
**Total:**			697,096	310,295	100,00	0,000	

2 Nov-546 (95:5)

Sample Name:	**Nov-546 (95:5)**	*Injection Volume:*	**10,0**
Vial Number:	**RB3**	*Channel:*	**UV_VIS_2**
Sample Type:	**unknown**	*Wavelength:*	**n.a.**
Control Program:	**AD_H_90Min_200_1_flow075**	*Bandwidth:*	**n.a.**
Quantif. Method:	**AD_H**	*Dilution Factor:*	**1,0000**
Recording Time:	**18.8.2013 19:35**	*Sample Weight:*	**1,0000**
Run Time (min):	**12,65**	*Sample Amount:*	**1,0000**

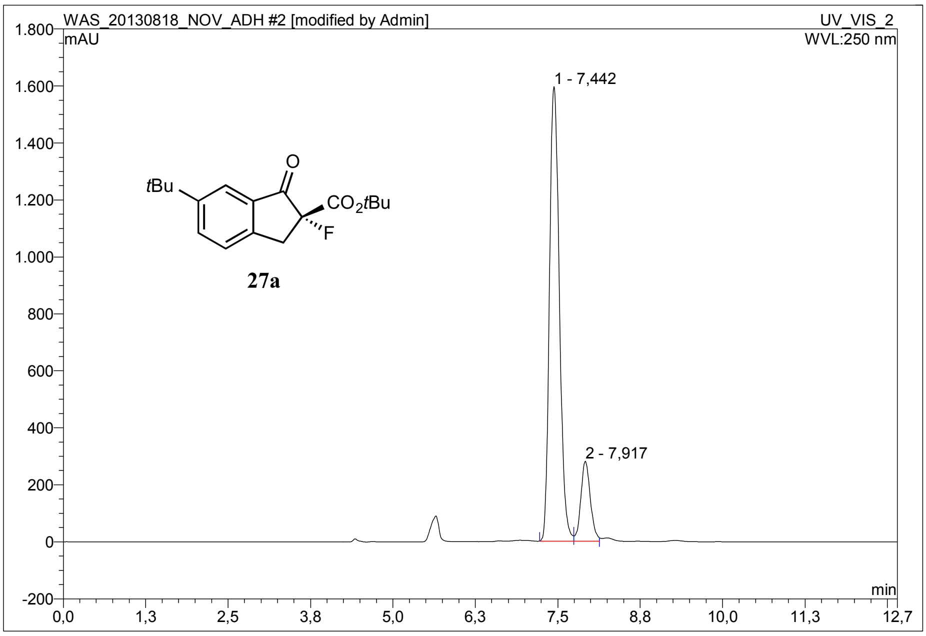

No.	Ret.Time min	Peak Name	Height mAU	Area mAU*min	Rel.Area %	Amount	Type
1	7,44	n.a.	1594,905	276,931	85,49	n.a.	BM *
2	7,92	n.a.	280,134	47,008	14,51	n.a.	M *
**Total:**			1875,039	323,939	100,00	0,000	

7 Nov-550 (90:10)

Sample Name:	**Nov-550 (90:10)**	*Injection Volume:*	**10,0**
Vial Number:	**RC6**	*Channel:*	**UV_VIS_2**
Sample Type:	**unknown**	*Wavelength:*	**n.a.**
Control Program:	**AD_H_90Min_200_1_flow075**	*Bandwidth:*	**n.a.**
Quantif. Method:	**AD_H**	*Dilution Factor:*	**1,0000**
Recording Time:	**20.8.2013 20:10**	*Sample Weight:*	**1,0000**
Run Time (min):	**15,00**	*Sample Amount:*	**1,0000**

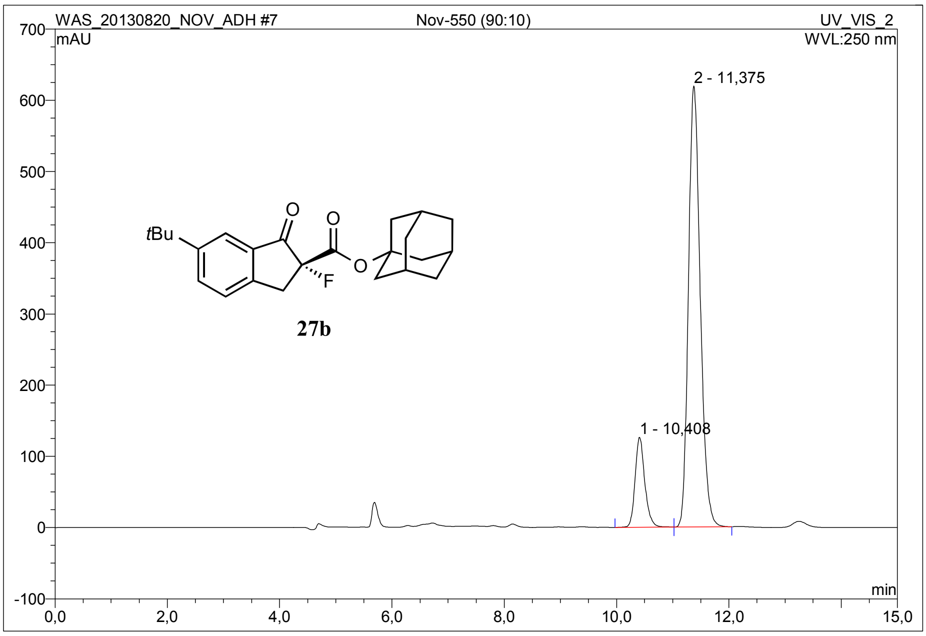

No.	Ret.Time min	Peak Name	Height mAU	Area mAU*min	Rel.Area %	Amount	Type
1	10,41	n.a.	126,352	25,351	14,39	n.a.	BMb
2	11,38	n.a.	619,120	150,840	85,61	n.a.	bMB
**Total:**			745,472	176,191	100,00	0,000	

1 Nov-535-19-22

Sample Name:	**Nov-535-19-22**	*Injection Volume:*	**10,0**
Vial Number:	**RA1**	*Channel:*	**UV_VIS_2**
Sample Type:	**unknown**	*Wavelength:*	**n.a.**
Control Program:	**OD_H_120Min_200_1_flow08**	*Bandwidth:*	**n.a.**
Quantif. Method:	**OD_H**	*Temperature/Column:*	**10**
Recording Time:	**8.8.2013 10:28**	*Flow ml/min:*	**0,800**
Run Time (min):	**35,89**	*Sample Amount:*	**1,0000**

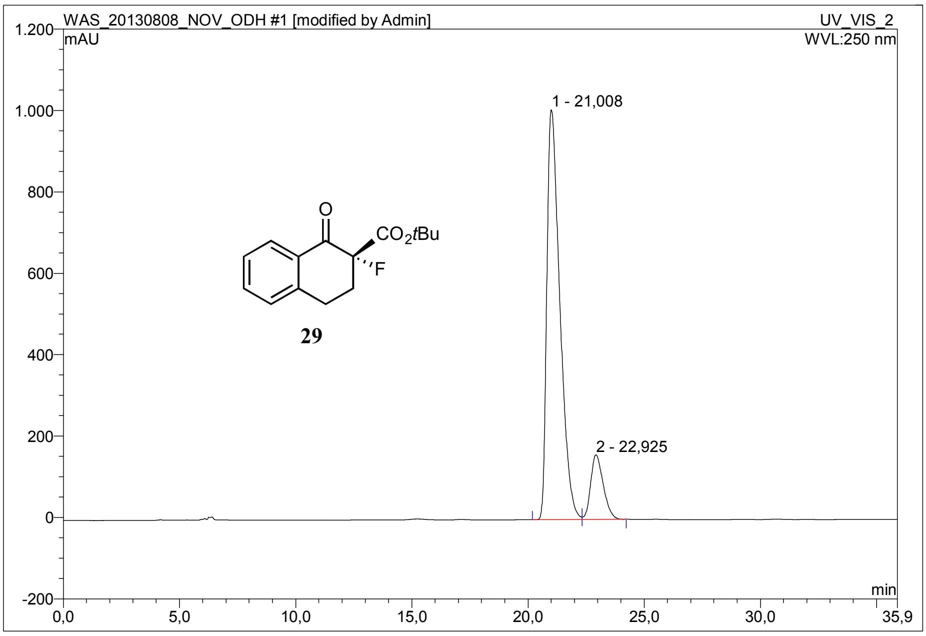

No.	Ret.Time min	Peak Name	Height mAU	Area mAU*min	Rel.Area %	Amount	Type
1	21,01	n.a.	1007,141	654,177	86,79	n.a.	BM *
2	22,93	n.a.	158,403	99,567	13,21	n.a.	MB*
**Total:**			1165,544	753,744	100,00	0,000	

200 n-Hexan : 1 iso-Prop.

2 Nov-548 konz (95:5)

Sample Name:	**Nov-548 konz (95:5)**	*Injection Volume:*	**10,0**
Vial Number:	**RB5**	*Channel:*	**UV_VIS_1**
Sample Type:	**unknown**	*Wavelength:*	**n.a.**
Control Program:	**AD_H_90Min_200_1_flow075**	*Bandwidth:*	**n.a.**
Quantif. Method:	**AD_H**	*Dilution Factor:*	**1,0000**
Recording Time:	**19.8.2013 10:24**	*Sample Weight:*	**1,0000**
Run Time (min):	**12,99**	*Sample Amount:*	**1,0000**

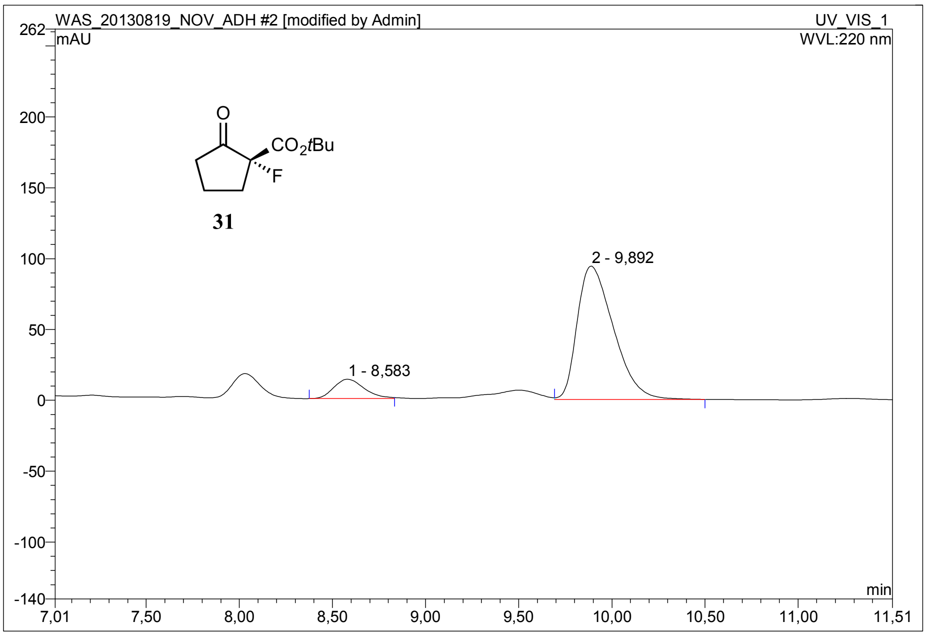

No.	Ret.Time min	Peak Name	Height mAU	Area mAU*min	Rel.Area %	Amount	Type
1	8,58	n.a.	13,666	2,594	10,89	n.a.	BM *
2	9,89	n.a.	94,126	21,232	89,11	n.a.	MB*
**Total:**			107,792	23,826	100,00	0,000	
